# Size-Transferable Prediction of Excited State Properties for Molecular Assemblies with a Machine Learning Exciton Model

**DOI:** 10.1021/acs.jpclett.4c03548

**Published:** 2025-03-03

**Authors:** Fangning Ren, Xu Chen, Fang Liu

**Affiliations:** † Department of Chemistry, Emory University, Atlanta, Georgia 30322, United States

## Abstract

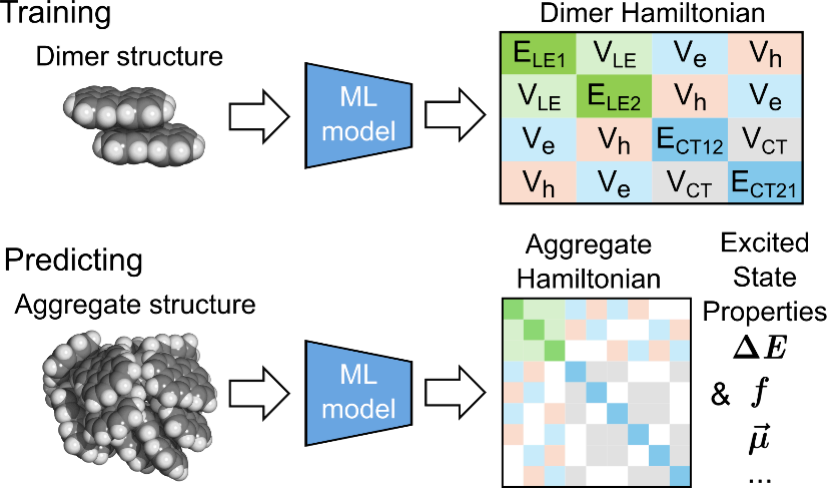

Computational modeling of the excited states of molecular aggregates faces significant computational challenges and size heterogeneity. Current machine learning (ML) models, typically trained on specific-sized aggregates, struggle with scalability. We found that the exciton model Hamiltonian of large aggregates can be decomposed into dimer pairs, allowing an ML model trained on dimers to reconstruct Hamiltonians for aggregates of any size. We also proposed a new method to address the phase-correction problem by introducing coupling terms’ approximations. Our model accurately predicted the excitation energies of the trimer and tetramer of perylene and tetracene and estimated S_1_ oscillator strengths of perylene aggregates. Leveraging our ML model, the optical gaps of nanosized perylene aggregates with up to 50 monomers are analyzed, qualitatively revealing the role of different couplings on their size dependency. Future work will explore transferability across different monomers to predict optical properties in heterogeneous assemblies.

Organic molecular assemblies have attracted the interest of chemists for over a decade due to their intriguing optical and photovoltaic activities. This includes the exciton dynamics of biological light-harvesting complexes,^1,2^ the charge transfer in organic solar cells^3,4^ and organic semiconductors,^5^ the photoluminescence of carbon dots,^6,7^ and the optical spectra of nanoaggregates formed during combustion^8^ or asphaltene aggregation.^9^ Among them, assemblies comprised of polycyclic aromatic hydrocarbons (PAHs) are the most widely studied ones as they can spontaneously assemble through intermolecular π - π stacking, while their optical and photovoltaic properties can be tuned not only by modifying the electronic structure of the monomer’s π - π conjugated system but also by adjusting the stacking arrangement between the monomers.^10^ This tunability provides a high degree of flexibility in designing photoactive systems for various applications.

Computational modeling of the excited state of PAH assemblies is vital for providing theoretical insights and designing guidance for photoactive systems. However, direct quantum mechanical (QM) calculations, represented by density-functional theory (DFT) and its time-dependent form (TDDFT), are computationally too expensive for large PAH nanoaggregates, and the resulting delocalized adiabatic states are not well-suited for analyzing their excitation characteristics. Methods for solid state materials, such as band gap theory, are not applicable to their disordered local structure.^6^ Moreover, all-electron QM calculations cannot directly provide localized diabatic states and couplings, the necessary parts for modeling the PAH assemblies’ intermolecular excitation energy^2,11^ and electron transfer^12−15^ processes based on the Marcus Theory.^16^

A computationally efficient alternative is the extended Frenkel exciton model,^17,18^ in which the aggregate’s excited states are treated as the linear combination of each monomer’s local excitation (LE) states and intermolecular charge transfer (CT) states. The energy and wave function of the aggregate’s adiabatic states can be obtained as the eigenvalues and the eigenvectors of the Frenkel Hamiltonian,
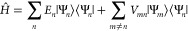
1

Here, the |Ψ_*n*_⟩ denotes a diabatic state basis function of an LE or CT state, *E*_*n*_ is the corresponding diabatic state energy, *V*_*mn*_ is the coupling between these two states, and the summation runs over all LE and CT states. The excited state characteristics can be derived from the eigenvectors of *Ĥ*. Various methods have been proposed to evaluate the matrix elements under the LE and CT state representation, which can be roughly classified into the eigenstate-based and fragment-based methods based on how the diabatic states are constructed. The eigenstate-based methods transform adiabatic excited states into diabatic states by maximizing a “localization function”, followed by constructing the excitonic Hamiltonian under such basis. Typical methods include the Boys localization,^19^ Fragment Charge Difference (FCD),^14^ Fragment Excitation Difference (FED),^20^ and the multistate FED-FCD approach (MS-FED-FCD).^12,21^ These methods require an excited state QM calculation of the whole system normally scales as *O*(*N*^3^) where *N* is the number of monomers. The fragment-based methods started from individual QM calculations on monomers to obtain localized diabatic states, followed by computing their energies and couplings to construct the Hamiltonian. The ab initio Frenkel Exciton model implemented in Q-Chem^22,23^ and TeraChem,^24,25^ together with the recent subsystem TDDFT-based MS-FED-FCD belongs to this class.^26^ They reduce the scaling to below *O*(*N*^2^) with an energy difference smaller than 0.1 eV from full-electron methods.^22−25^

Despite its success in small PAH aggregates,^27−29^ the fragment-based Frenkel exciton model still requires significant computing resources for modeling the excited states for nanosized aggregates. Since an aggregate’s observed properties (e.g., absorption/fluorescence spectrum) are the statistical average over multiple conformations,^10,30,31^ accurately modeling these properties necessitates adequate sampling of the conformational space. However, the conformational space expands exponentially with the number of monomers, making this task computationally challenging. In recent years, many studies have tried to overcome this obstacle via machine-learned (ML) exciton models,^32−41^ which predict the Frenkel Hamiltonian matrix elements for aggregates of a fixed size based on their conformation. The training data are reference Frenkel Hamiltonians for molecular dynamics (MD) sampled aggregate conformations of the fixed size, calculated by DFT or semiempirical methods. These ML exciton models have been applied to molecular spectrum prediction,^38^ excited state dynamics,^32,35,42^ and exciton and charge transfer simulation in organic semiconductors^43−45^ and light-harvesting complexes.^46^ However, these models often require expensive reference Hamiltonians for large aggregates and cannot be directly applied to molecular assemblies exhibiting size-heterogeneity, such as asphaltene aggregates^47^ and carbon dots.^6^ In contrast, some recent ML exciton models for biomolecules exhibit size-transferability,^35,38,46,48,49^ wherein a multichromophore system’s Frenkel Hamiltonian is predicted by a model trained on QM calculations of subsystems containing one or two chromophores. Nevertheless, these models do not yet incorporate CT states and related couplings, which are essential for accurately predicting the optical properties of PAH aggregates.^50−52^

To address these obstacles simultaneously, we introduced a size-transferable ML exciton model with LE-CT coupling. We train an ML model on a dimer data set to predict dimers’ Frenkel Hamiltonian elements, which are then used to construct the Frenkel Hamiltonian for an arbitrarily sized homogeneous aggregate (Figure 1). This approach leveraged the fact that for nonpolar PAH molecules when the monomer’s S_0_-S_1_ transition is mostly HOMO–LUMO transition, a homogeneous aggregate’s Frenkel Hamiltonian elements can all be directly obtained from or accurately approximated by its constituent dimer’s Frenkel Hamiltonian elements.^53^ Our method can predict the low-lying excited state energy of nanosized aggregates containing up to 50 monomers. This approach avoids the expensive QM training data generation for large aggregates and greatly enhances the transferability of the ML exciton models.

**Figure 1:**
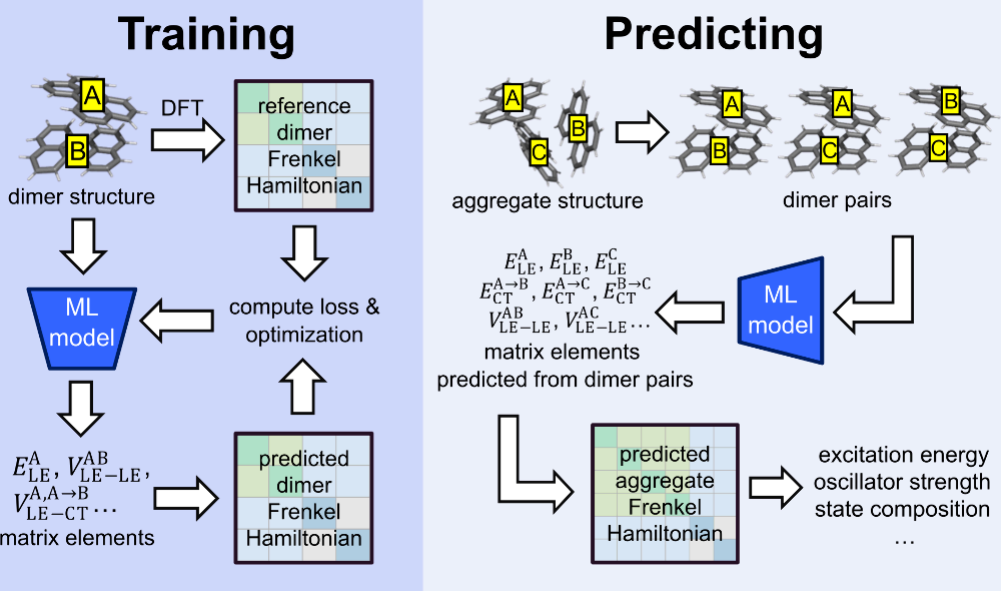
Workflow for the ML exciton model in this work. The left plot illustrates the training workflow in which the training set is generated with TeraChem’s ab initio Frenkel exciton model implementation,^42^ followed by ML model training and evaluation. The right panel describes the prediction process, where the given aggregate structure is split into dimer pairs, followed by utilizing the model to predict the corresponding terms. The excited state properties are obtained by solving the eigenvalue problem of the constructed Hamiltonian.

Our reference Frenkel Hamiltonian was obtained by the ab initio exciton model implementation of TeraChem.^24,25^ which supports multiple LE states per monomer and two CT states per dimer pair. For simplicity, we only consider the first LE state of each monomer. Taking a trimer as an example, the Frenkel Hamiltonian takes the form shown in Figure 2, where the diagonal terms are LE and CT state energies, and the off-diagonal terms are LE-LE, LE-CT, and CT-CT couplings. The LE state energy on monomer A can be written as
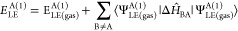
2

**Figure 2:**
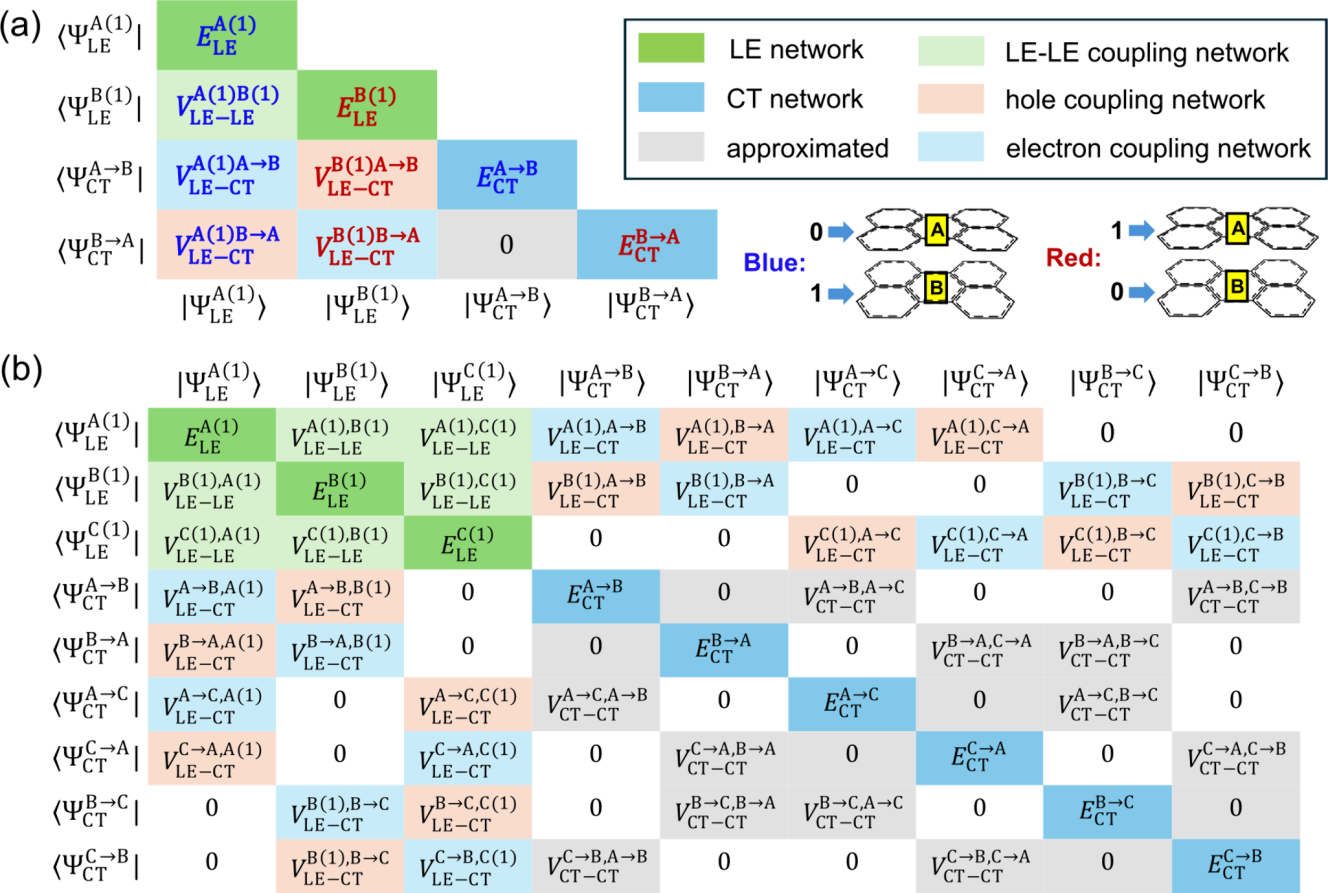
Scheme for predicting the Hamiltonian matrix using ML models. (a) Prediction of matrix elements from a dimer pair of monomers A and B. Elements sharing the background color are either predicted by the same neural network or approximated (gray background). For terms in blue font, atoms are assigned belonging labels (as defined in eq 6) of 0 for monomer A and 1 for monomer B. For terms in red font, these labels are reversed. (b) Hamiltonian matrix for a trimer consisting of monomers A, B, and C. Elements sharing the same background color corresponding to the legend in panel a are predicted by their respective networks, while gray-background elements are approximated. Zero-value elements with a gray background correspond to CT-CT couplings with negligible magnitude and are treated as approximately zero, whereas those with a white background are exactly zero.

Here, the term *E*_LE(gas)_^A(1)^ and Ψ_LE(gas)_^A(1)^ indicate the first excitation energy and excited state wave function of an isolated monomer A in the gas phase. Δ*Ĥ*_BA_ accounts for the impact of the atomic charges of another monomer B on A. Similarly, the CT state energy for A transferring an electron to B can be written as
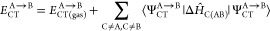
3

Here, the terms *E*_CT_^A→B(gas)^ and Ψ_CT_^A→B^ indicate the excitation energy and excited state wave function of monomer A in the gas phase, which are computed in a ΔSCF-like way to avoid the incorrect asymptote behavior of some DFT functionals.^24^ Δ*Ĥ*_C(AB)_ accounts for the impact of the atomic charges of another monomer C on the dimer pair AB. It is worth noting that if there are point charges representing the environment, such as polar solvents or protein backbones, their impact will also be added into Δ*Ĥ*_BA_ and Δ*Ĥ*_C(AB)_. All diagonal terms of timers and larger aggregates can be directly evaluated from constituent dimers’ Frenkel Hamiltonians elements. The evaluation of off-diagonal terms (detailed mathematical expressions available in the original article^24^) falls into three cases: some are directly evaluated from constituent dimers’ Hamiltonian terms, some can be approximated from dimers’ Hamiltonian terms, and others are negligible and set to 0. Taken together, the exciton model decomposes the intermolecular interaction within the entire aggregate into the interaction between dimers (or trimer if needed to account Δ*Ĥ*_C(AB)_). Furthermore, we found the impact of Δ*Ĥ*_C(AB)_ on low-lying states’ excitation energies for nonpolar systems like perylene and tetracene aggregates to be negligible (Figures S1 and S2). This allows us to predict larger aggregate Hamiltonian based on a dimer Hamilton data set that comprehends sufficiently sampled dimer conformations. The reference dimer Hamiltonian can also be constructed by other fragment-based approaches with multiple LE and CT states,^22,23,54^ but TeraChem’s GPU-accelerating feature greatly accelerates the data set generation.^55^ Hence, we first focus on building ML models to predict the TeraChem calculated dimer Hamiltonian and then extend our approach to larger aggregates, which will be discussed in later paragraphs.

We choose perylene, the backbone of the famous rylene dye family,^56^ as our test system and curate a diverse training set, PrDim, composed of 24000 perylene dimer conformers. The PrDim set comprises three subsets: the COM4A set of 8000 energetically favorable face-to-face stacking dimers (H-aggregates),^57^ the NST5A set of 8000 head-to-tail stacking (J-aggregate) or t-shape stacking dimers, and the SEP5A set of 8000 distantly separated dimer conformations to cover the asymptotically decaying couplings in large aggregates. The reference Frenkel Hamiltonians for COM4A and NST5A sets are generated with the ab initio exciton model, whereas that of SEP5A are generated with approximations to reduce the computational cost without sacrificing accuracy (see Computational Details). With the same procedure, another dimer data set called TtDim is generated for tetracene, the prototype for organic semiconductors, to demonstrate the transferability of our method to other PAH monomers.

We train ML models to predict the dimer Frenkel Hamiltonian matrix elements based on the dimer conformation, using a modified TorchANI architecture.^58^ The original TorchANI model takes the molecular coordinates {*R⃗*_*i*_} as the input, embeds the chemical environment of each atom *i* of chemical element *X* into an atomic environment vector (AEV), *G⃗*_*i*_^*X*^, passes each AEV through a respective element-specific neural network (NN) to obtain atomic energy *E*_*i*_^*X*^, and sums them up to output the molecule’s total energy, *E*_T_. The mapping from the input to the output can be summarized as
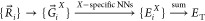
4

In contrast, the mapping for our modified TorchANI architecture is as follows:
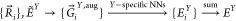
5

Here, the output *E*^*Y*^ is Hamiltonian matrix element of type *Y*; the additional input *Ẽ*^*Y*^ is a proper approximation of *E*^*Y*^ computed after aligning a reference monomer wave function to each of the monomers in the dimer (see Computational Details); *G⃗*_*i*_^*X*,aug^ is the augmented AEV for the *i*-th atom; and *E*_*i*_^*Y*^ is the atomic contribution to *E*^*Y*^. Our mapping is different from the original TorchANI mapping [eq 4] in two aspects. First, the NNs are specific to the type of matrix element to predict (*Y*) rather than the chemical element type (*X*). As shown in Figure 2, we train an “LE network” for predicting *E*_LE_^A(1)^ and *E*_LE_^B(1)^, a “CT network” for *E*_CT_^A→B^ and *E*_CT_^B→A^, a “LE-LE coupling network” model for *V*_LE-LE_^A(1)B(1)^, and two types of LE-CT coupling models, namely, the “hole coupling network” for *V*_LE-CT_^A(1)B→A^ and *V*_LE-CT_^B(1)A→B^, and the “electron coupling network” for *V*_LE-CT_^A(1)A→B^ and *V*_LE-CT_^B(1)B→A^.^59^ The CT-CT coupling between two reversed CT states, e.g., |Ψ_CT_^A→B^⟩ and |Ψ_CT_^B→A^⟩, has a magnitude smaller than 1.0 meV and is therefore approximated as zero.^31,53^ The remaining nonzero couplings in the trimer Hamiltonian can also be approximated, as we will discuss later. The second difference is the augmented AEV,
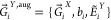
6where *G⃗*_*i*_^*X*^ is the original TorchANI AEV for the *i*-th atom; *b*_*i*_ is a Boolean “belonging label” to indicate whether the atom belongs to monomer A or B; and *Ẽ*_*i*_^*Y*^ is the atomic decomposition of the approximation *Ẽ*^*Y*^. The label *b*_*i*_ enables a single NN (e.g., the LE network) to predict two different matrix elements under the same category (e.g., *E*_LE_^A(1)^ and *E*_LE_^B(1)^) by reversing each atom’s label. For perylene and tetracene, we train each model separately. Additional details about model architecture are illustrated in Figure S3 and explained in Text S1.

The *Ẽ*_*i*_^*Y*^ term enhances the model’s data efficiency because of the strong correlation between the model output (*E*^*Y*^) and its approximation (*Ẽ*^*Y*^). As shown in Figure S4, computing *Ẽ*_*i*_^*Y*^ starts from calculating the reference wave functions for the neutral, cationic, and anionic states (Ψ_ref_, Ψ_ref_^+^, Ψ_ref_^–^) based on a DFT-optimized monomer geometry {*R⃗*_*i*_^ref^}, followed by fitting the atomic charge for Ψ_ref_^+^ and Ψ_ref_^–^ with the restrained electrostatic potential (RESP) approach^60^ (denoted as  respectively). The atomic transition charges {*q*_i_^ref,tr^} for the monomer’s S_0_ – S_1_ excitation are also fitted by the TrESP approach^61,62^ based on a TDDFT calculation. Given monomer A’s geometry {*R⃗*_*i*_^*A*^}, in a certain dimer AB, we first compute the translation vector and rotation matrix that optimally aligns {*R⃗*_*i*_^ref^}, with {*R⃗*_*i*_^*A*^}. Then, an approximate monomer wave function Ψ_A_^apx^ is constructed by moving the atomic orbital (AO) basis function centered at *R⃗*_*i*_^ref^ to *R⃗*_*i*_^*A*^, followed by adjusting molecular orbital (MO) coefficients within the same AO shell based on the rotation matrix. The {*q*_*i*_^ref,+^},{*q*_*i*_^ref,−^} and {*q*_i_^ref,tr^} are assigned to {*R⃗*_*i*_^*A*^} without changing their magnitude. Finally, we adjust the sign of HOMO and LUMO coefficients in Ψ_A_^apx^ to align with the wave function evaluated by TeraChem’s exciton model and modify {*q*_i_^ref,tr^}’s sign based on the transition dipole moments of the reference LE state. *Ẽ*^*Y*^ for CT energies *E*_CT_^A→B^(*E*_CT_^B→A^) is the Coulombic interaction between {*q*_i_^ref+^} on A (B) and {*q*_i_^ref,–^} on B (A), while that of *V*_LE-LE_^A(1)B(1)^ is the interaction between {*q*_i_^ref,tr^} placed on A and B. *Ẽ*^*Y*^ for hole (electron) couplings is the overlap integral between the HOMO (LUMO) of Ψ_A_^apx^ and Ψ_B_^apx^, denoted by *S*_HOMO_^AB^ (*S*_LUMO_^AB^). Given that PAHs’ frontier orbitals are predominantly composed of p-orbital characteristics,^63,64^ to speed up the calculation without losing much accuracy, *S*_HOMO_^AB^(*S*_LUMO_^AB^) are computed as the overlap between p-type basis functions multiplied by their HOMO (LUMO) coefficients. Finally, all *Ẽ*^*Y*^ are decomposed into *Ẽ*_*i*_^*Y*^ to augment the AEV (see Text S2 for details). The accuracy of our ML model in predicting individual terms in the dimer Hamiltonian is shown in Table 1. Our model can predict the coupling terms evaluated by TeraChem’s ab initio exciton model with excellent accuracy, with all *R*^2^ values over 0.99. The mean absolute error (MAE) is below 10 meV for all matrix elements except for the *E*_CT_^B→A^ terms (MAE = 10.29 meV). This slightly larger MAE may be attributed to the broader range of values it spans (with a standard deviation of over 0.6 eV), as the energy of CT states is more sensitive to the dimer configuration than LE states. The CT-CT couplings between the CT states, |Ψ_CT_^A→B^⟩ and |Ψ_CT_^B→A^⟩, are not evaluated as they are usually negligible compared to other couplings. Similar accuracy is also observed for tetracene aggregates (Table S1). It is worth noting that the ML model can also reproduce the phase of the off-diagonal terms of dimer Hamiltonians, because the approximations, including overlap integrals and atomic transition charges, uniquely determine the phase of the dimer wave function. This feature of our approach is promising in predicting the excited state properties for larger aggregates.

**Table 1:** Test Set Accuracy of the ML Model on Various Hamiltonian Terms of Perylene Dimers in the PrDim Set, Reported as the Mean Absolute Error (MAE) and Coefficient of Determination (*R*^2^) with Respect to Reference Ab Initio Exciton Model Results^a^

term	MAE (meV)	*R*^2^	reference value standard deviation (meV)
*E*_LE_^A(1)^	6.53	0.993	95.63
*E*_LE_^B(1)^	6.52	0.993	95.94
*E*_CT_^A→B^	9.95	1.000	628.65
*E*_CT_^B→A^	10.29	1.000	635.68
*V*_LE-LE_^A(1),B(1)^	3.11	0.994	66.46
*V*_LE-CT_^A(1),A→B^	5.04	0.992	94.36
*V*_LE-CT_^A(1)B→A^	5.57	0.995	125.27
*V*_LE-CT_^B(1)A→B^	5.82	0.994	124.02
*V*_LE-CT_^B→A^	4.97	0.992	92.57

a The standard deviation of the reference values for each term is provided in the last column for comparison.

However, when constructing the Hamiltonian of larger aggregates (trimer and above), one still needs to overcome the phase problem illustrated in Figure 3. The Frenkel exciton model utilizes the monomer ground state and excited state wave functions as the basis set, which can have arbitrary phases due to the nature of most QM software. Each phase combination of the monomer wave functions corresponds to a sign combination of the coupling terms, resulting in a series of different but unitary similar Hamiltonian matrices. For example, the three LE-LE coupling terms of a perylene trimer’s Frenkel Hamiltonian have four valid sign combinations based on the phase of the three monomer wave functions (Figure 3). However, the ML model trained on the dimer data sets does not know the correct sign combination of larger aggregates’ coupling terms and may yield unphysical Hamiltonian matrices not unitarily similar to the valid Hamiltonian. For instance, a perylene trimer’s LE-LE coupling terms may have 8 sign combinations if each term’s sign is randomly assigned. However, 4 out of the 8 combinations are unphysical, not corresponding to any possible combinations of the monomer wave function phases. Such a phase problem was mentioned in previous studies about excited state ML^65^ and several solutions have been proposed. This includes (1) aligning the wave function’s phase or CI coefficient between neighboring MD snapshots^66,67^ and (2) correcting the phase based on the molecule’s geometry.^48^ However, approach (1) cannot be applied to randomly sampled dimer conformations, while approach (2) may be unavailable in highly symmetric planar PAH systems. Another alternative, the phase-free training approach,^36,68^ allows the model to learn the phase combination for a given system’s Hamiltonian without prior phase correction, but such learned combination cannot be transferred to larger aggregates. Here, we find a solution for planar PAH aggregates of any size. When evaluating the Frenkel Hamiltonian of large aggregates, we align Ψ_ref_ to each monomer in the aggregates. Then, we computed the approximations *Ẽ*^Y^ between each dimer pair by our approach described in previous paragraphs, but the sign of MO coefficients and TrESP charges are not modified. These approximations allow the model to infer the signs of the coupling terms and yield a physically valid Hamiltonian matrix and thus predict the correct eigenvalues (excitation energies) and eigenvectors (aggregate wave function).

**Figure 3:**
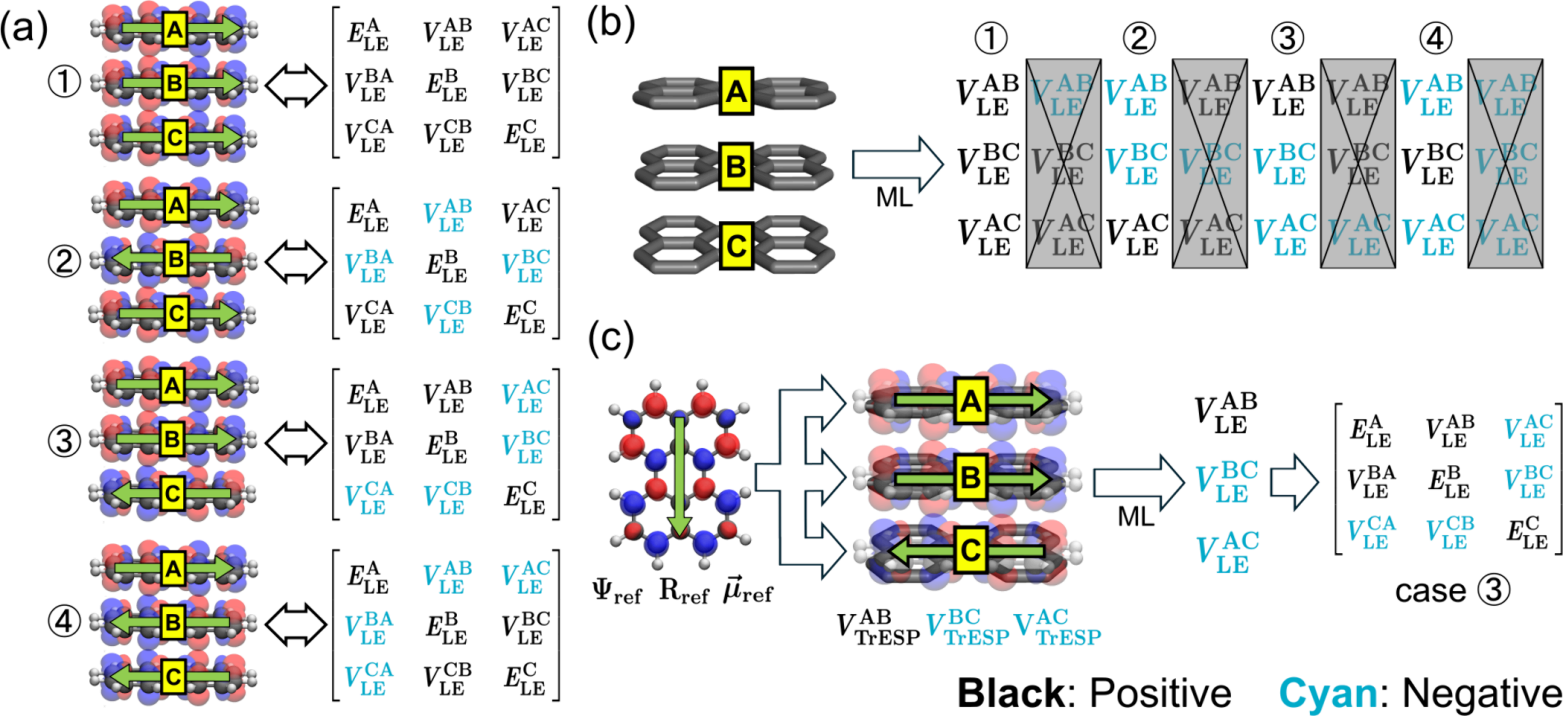
Illustration of our approach to solving the phase problem of ML predicted Frenkel Hamiltonians, using a perylene trimer as an example. Only the first monomer LE states are considered. Negative matrix elements are colored cyan, while positive elements are colored black. (a) Different phase combinations of the three monomer wave functions lead to distinct but unitarily similar Frenkel Hamiltonians with varying signs in the coupling terms. (b) Using only the trimer coordinates as input, the ML model predicts 8 possible sign combinations of the three LE-LE coupling terms. Four of these combinations, which are unphysical and do not correspond to any phase configuration of the monomer wave functions, are crossed out in the figure. (c) To ensure correct sign combinations of the LE-LE coupling terms, a reference monomer wave function is aligned with each monomer via translation and rotation. One valid sign combination is then determined through the TrESP charge interaction among the reference wave functions. All isosurfaces represent the S_0_-S_1_ transition density of 0.002 au.

Another challenge for extending our ML approach to a larger aggregate is the remaining nonzero CT-CT coupling between two CT states sharing the same monomer. As shown in Figure 2, for a trimer composed of monomers A, B, and C, *V*_CT-CT_^A→B,A→C^ is a nonzero coupling between the CT states |Ψ_CT_^A→B^⟩ and |Ψ_CT_^B→A^⟩. However, these terms are not included in the dimer Hamiltonian, so the ML models trained on dimers cannot directly evaluate their values. Fortunately, based on TeraChem’s implementation of the exciton model,^24^ such CT-CT couplings can be accurately approximated when the monomer S_0_-S_1_ transition is mostly a HOMO–LUMO transition. Perylene and tetracene happen to satisfy this condition (CI coefficient >0.95). In these cases, the CT-CT coupling (e.g., *V*_LE-CT_^A→B,A→C^) can be approximated by multiplying a Hartree–Fock (HF) exchange partition dependent factor with the corresponding LE-CT coupling (e.g., *V*_LE-CT_^B(1),B→C^), which are readily available from the ML models trained by the dimers (see Text S3 for detailed proof). We conduct test calculations on 100 perylene and tetracene trimers and confirmed the accuracy with MAE < 3 meV (Figures S5 and S6). More discussions about the factor between LE-CT and CT-CT couplings are available in Text S4, Figure S7, and Table S2 in our Supporting Information.

To test the transferability of our model trained on PrDim, we curate three out-of-sample (OOS) test sets of perylene dimer, trimer, and tetramers, namely oPrDim, oPrTri, oPrTet for perylene, and oTtDim, oTtTri, and oTtTet for tetracene (see Computational Details). Figure 4 shows the prediction error of the excited state energy of the OOS data sets, each of which contains 500 conformations. When the adiabatic state index is lower than the number of monomers in the aggregate, the ML-exciton model achieves satisfying accuracy with an MAE of around 15 meV when compared to the ab initio exciton model, and around 30 meV when compared to all-electron TDDFT. However, the errors significantly increase for higher states. This is because higher adiabatic states have more contributions from higher monomer LE states, but the predicted Hamiltonian only includes the first LE state of every monomer. The larger discrepancy between our ML exciton model’s and TDDFT results is attributed to the difference between the exciton model and TDDFT rather than the ML model’s performance. Specifically, it stems from two primary effects: the positive systematic error caused by limited monomer LE states (configuration delocalization) and the neglect of polarization effects on the monomer’s ground and excited state wave function (orbital delocalization). These effects were previously referred to as “orbital and configuration delocalization” by Gao et al.^29^ Adding more monomer LE states reduces the systematic error in the S_1_ energy of the exciton model but cannot eliminate the error caused by orbital delocalization effects (Figure S8). Nevertheless, the discrepancy in excited state energy between the ML-predicted Hamiltonian and all-electron TDDFT is within 0.05 eV, demonstrating the model’s ability to predict the excited state properties of aggregates.

**Figure 4:**
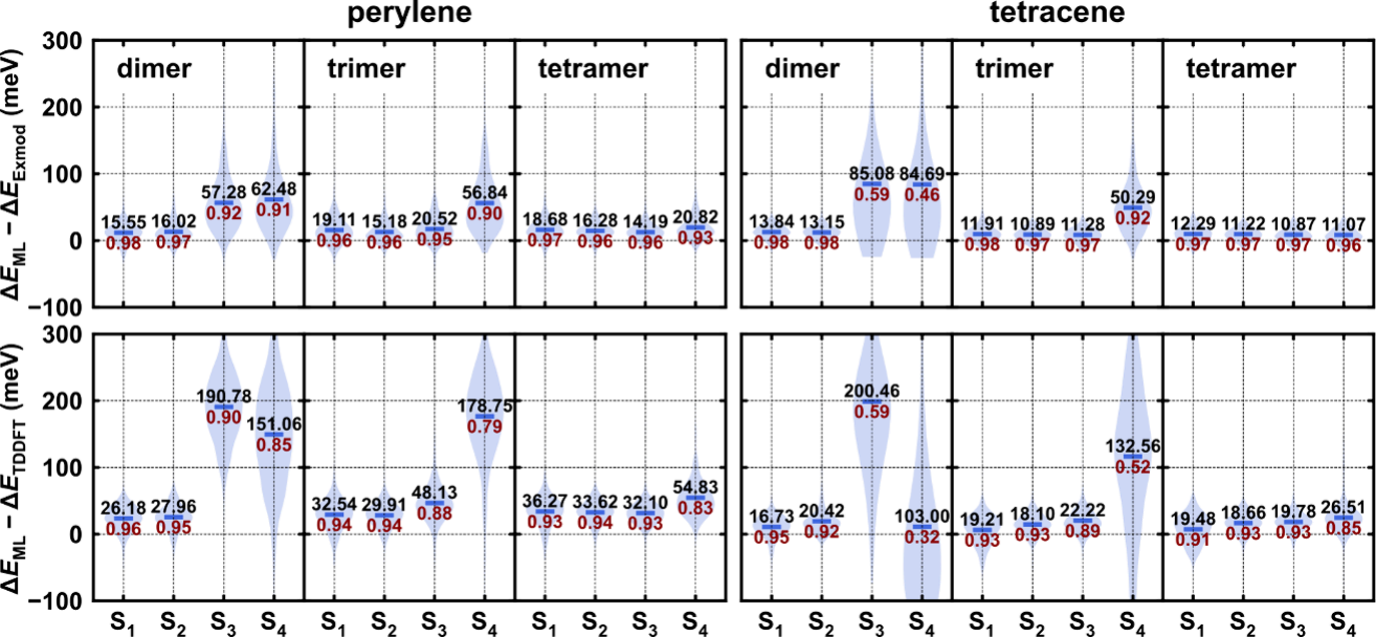
Out-of-sample test error of the ML exciton model’s prediction of the first four excited state energies of aggregates of size 2–4, compared with the ab initio exciton model and all-electron TDDFT. Corresponding mean absolute errors (MAE) are shown in black text above the error bar, while *R*^2^ values are shown in red text below the error bars. The perylene’s dimer, trimer, and tetramer results (left) are for the out-of-sample (OOS) test sets oPrDim, oPrTri, and oPrTet, while the tetracene’s dimer, trimer, and tetramer results (right) are for the oTtDim, oTtTri set, and oTtTet. Each OOS set contains 500 conformations.

To demonstrate the balance between the accuracy and efficiency of our model, we summarized the timing for predicting the OOS data sets and compared them with TeraChem’s exciton model and all-electron TDDFT (Table 2). The ML Hamiltonian is over 3 orders of magnitude faster than QM methods. The most time-consuming step for constructing the ML Hamiltonian is calculating the overlap integrals, a necessary approximation for reproducing the phase of LE-CT couplings. Some fast and analytic methods have been proposed to evaluate couplings, such as the TrESP approach^61,62^ for LE-LE couplings and analytic overlap method (AOM) for LE-CT and CT-CT couplings.^63,69,70^ They have been used to construct Frenkel Hamiltonians for optical properties prediction or nonadiabatic dynamics simulation.^15,71,72^ However, in our case, the diagonal elements (LE and CT state energies) still need to be predicted by the ML model. As our model can yield diagonal and off-diagonal elements simultaneously, the incorporation of TrESP and AOM has negligible impact on computational efficiency (see Text S5 for proof) while increasing the MAE for all OOS data sets for around 5 meV (Figure S9 and Table S3). Therefore, the ML model strikes a balance between accuracy and efficiency.

**Table 2:** Timings for the ML-Exciton Model for Different OOS Data Sets^a^

data set	number of monomers	TrESP and RESP charge interaction time (s)	overlap integral time (s)	NN propagation time (s)	total time (s)	ab initio exciton model (h)	all-electron TDDFT (h)
oPrDim	2	0.05	6.41	0.79	7.25	31.88	23.59
oPrTri	3	0.11	9.11	1.46	10.68	49.55	45.68
oPrTet	4	0.24	14.01	2.80	17.05	77.02	78.29
oTtDim	2	0.04	5.92	0.92	6.88	21.26	15.25
oTtTri	3	0.11	7.96	1.52	9.59	40.45	31.98
oTtTet	4	0.23	11.83	2.88	14.91	60.30	56.71

a Each OOS data set contains 500 aggregate structures. The ML timings are measured with an Intel(R) Xeon(R) Silver 4210R CPU and one Nvidia RTX A4000 GPU, while the timing for QM calculations is the total GPU hours used with the same hardware set.

In addition to excitation energy, one can derive the approximated excited state wave function from the eigenvector of the ML-predicted Frenkel Hamiltonian to evaluate other excited state properties. Here, we demonstrate the prediction of the S_1_ oscillator strength of different aggregates based on our ML exciton model. Since the model is not directly trained to evaluate monomer or dimer oscillator strengths, we employ the following approach: We first align the reference monomer coordinate and its S_1_ state transition dipole moment (TDM) with each monomer in the aggregate, and then sum up the aligned TDM according to the LE state coefficients solved by the ML-predicted model Hamiltonian (see Figure S10) to approximate the total TDM (*μ⃗*_ML_). The ML-predicted oscillator strengths (*f*_ML_) can be computed by^73^
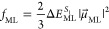
7

Here, Δ*E*_ML_^S_1_^ is the predicted S_1_ excitation energy and *μ⃗*_ML_ is the S_1_ TDM computed by the above procedure. The *f*_ML_ exhibits a very strong linear correlation (*R*^2^ > 0.96) with the *f*_Exmod_ obtained from the ab initio exciton model (Figure 5), even though the model is not trained to predict the fluctuation of the monomer oscillator strength caused by conformation change. The *f*_ML_ value is generally slightly smaller than *f*_Exmod_, because the DFT optimized reference monomer wave function has a slightly smaller S_1_ TDM (6.94 D) than the average monomer TDM of MD sampled conformers (7.61 D) (Figure S11). The correlation between *f*_ML_ and the TDDFT calculated oscillator strength, *f*_TDDFT_, is weaker, with an *R*^2^ of ca. 0.90 (Figure 5). This is likely due to the ML model’s neglect of orbital delocalization, the same reason for the discrepancy in energy prediction between the ab initio exciton model and TDDFT. However, unlike the energy prediction, configuration delocalization does not seem to be the main reason for the discrepancy here, because adding more LE states does not improve the correlation between *f*_Exmod_ and *f*_TDDFT_ (Figure S12). In summary, the model can estimate the oscillator strength of aggregates of any size based on their conformation, despite not being explicitly trained for this task.

**Figure 5:**
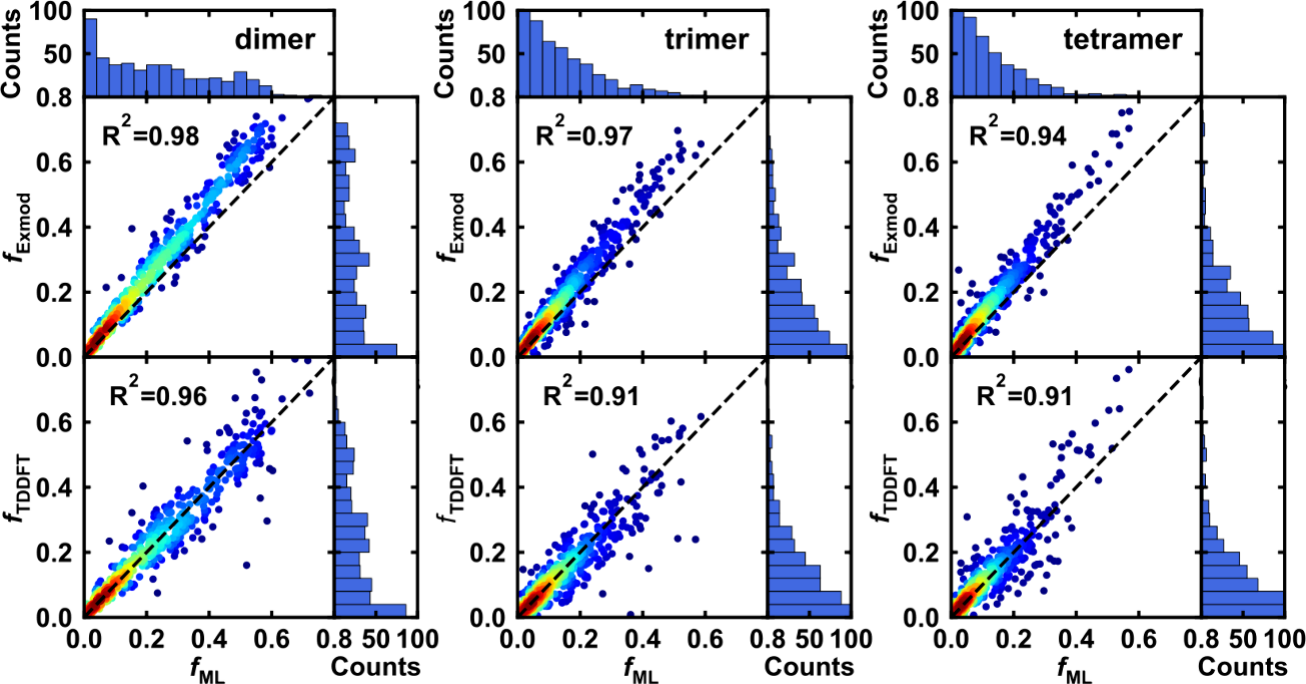
Parity plot and marginal distribution of the S_1_ oscillator strength (unitless) predicted by our ML model (*f*_ML_) compared with the exciton model (*f*_Exmod_) and all-electron TDDFT (*f*_TDDFT_) results. The dimer, trimer, and tetramer results are for the out-of-sample test sets oPrDim, oPrTri, and oPrTet.

Finally, we demonstrate the capability of our ML exciton model in investigating large, nanosized aggregates. We focus on predicting the optical gap of nanosized PAH aggregates, which is of interest to combustion chemists due to their quantum dot-like behaviors.^8,57,74,75^ Although the quantum confinement-like optical gap size dependency was observed in the experiment,^75^ excited state theoretical studies of PAH aggregates are limited by significant computational overhead, making it difficult to achieve statistically reliable results from sufficient conformations. Consequently, previous works only studied the size dependency of the HOMO–LUMO gap (electronic gap) of homogeneous PAH aggregates and roughly attributed the size dependency to intermolecular pi-pi interactions.^57,75^ With the machine-learned Frenkel Hamiltonian, we can overcome the computational obstacles for nanosized aggregates and further explore the mechanism of the quantum confinement behavior.

For perylene aggregates containing 1 to 50 monomers, we collect 500 conformations of each size from 5 ns-long gas-phase MD trajectories (see Text S6 for the detailed procedure). Then, we use the trained ML model to predict the full Frenkel Hamiltonian and optical gap (S_1_ excitation energy) for each conformer. Finally, we calculate the optical gap’s mean and standard deviation over all conformations at each size to observe how the optical gap varies with the number of monomers. Additionally, we build partial Frenkel Hamiltonians with the LE-LE couplings only or no couplings at all, and diagonalize to obtain optical gaps. A comparison of these partial Frenkel Hamiltonians’ results with our full Frenkel Hamiltonian results demonstrates the impact of different couplings (Figure 6).

**Figure 6:**
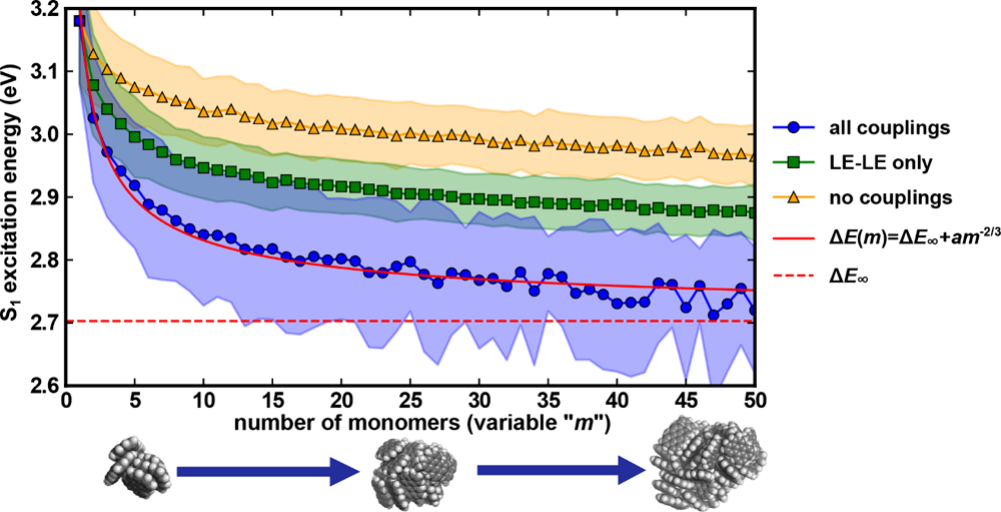
Evaluation of the optical gap of perylene aggregates using ML exciton models with varying coupling types. Results are shown for models considering all types of couplings (blue), only LE-LE couplings (green), and no couplings (yellow). Each data point represents the average optical gap calculated over 500 conformers, with one standard deviation depicted by the shaded area of the corresponding color. The red solid line represents the fitting of Δ*E*_∞_ + *am*^–2/3^ with Δ*E*_∞_ drawn as a red dashed horizontal line.

According to the ML full Frenkel Hamiltonian’s result, the optical gap decreases by 0.48 eV due to aggregation from the isolated perylene monomer to an infinite-sized aggregate (Figure 6). This result agrees well with the 0.40 eV red shift of the experimental first absorption peak from perylene monomer^76^ to α-perylene crystal,^77^ demonstrating our model’s capability to capture the size-dependent trend of aggregates’ optical gap. However, our model overestimates the optical gaps for both the monomer (3.18 eV) and the infinite-sized aggregate (2.70 eV) compared to the respective experimental values of 2.98 eV^76^ and 2.58 eV.^77^ This overestimation is not due to the accuracy of the ML model, but is likely caused by the errors of the TDDFT method and the approximate density functional used for generating the training data. More specifically, this discrepancy is caused by the systematic error of +0.21 eV of TD-wB97X-D3 in computing the monomer LE state energies,^78−80^ as manually subtracting 0.21 eV from the LE state energies in the Hamiltonian improves the agreement with experiment (2.97 eV for monomer and 2.55 eV for infinite-sized aggregate) (Figure S13). However, this is not theoretically rigorous as the CT energies and coupling elements may also require DFT functional-specific corrections, which have only been explored in individual studies under different theoretical frameworks.^81^ Therefore, our discussion focuses on the uncorrected results.

To test the aggregate’s quantum confinement effect, we follow the approach of Wang *et al*.^75,82^ and a few other groups^57,83^ and examine the following relation between the optical band gap, Δ*E*, and the number of monomers in the aggregate, *m*:
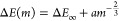
8

Here, Δ*E*_∞_ is the optical gap for an infinite-sized aggregate, and *a*=Δ*E*_∞_-Δ*E*(1). Following Eq. 8, a fitted curve with Δ*E*_∞_ = 2.70 eV and *a* = 0.48 eV is plotted in Figure 6. The results calculated by our full ML-exciton model agree well with the fitted curve, with a small MAE of 0.015 eV. This trend indicates the quantum confinement effect on the optical gap of perylene aggregates and is consistent with previous computational studies of the HOMO–LUMO gap of homogeneous PAH aggregates.^57,83,84^ To validate our ML model’s accuracy on larger aggregates, we randomly selected 5 conformers for aggregates of size *m* = 5, 10, 15, 20, 25, 30, 35, 40, and 45, and computed reference results with the ab initio exciton model and all-electron TDDFT for each conformer. Our ML exciton model’s performance on larger aggregates is similar to that observed by dimers, trimers, and tetramers (Figure S14), demonstrating the reliability of the results obtained by the machine-learned Hamiltonian.

We further examine the contributions from different couplings by comparing the results of full Frenkel Hamiltonians with that of partial Hamiltonians only considering the LE-LE couplings and no couplings at all. Surprisingly, even when all couplings are neglected, the aggregate S_1_ energy still decreases, mainly due to statistical principles (Text S7, Figures S15 and S16). The long-range coupling between LE-LE states lowers the optical gap by around 0.1 eV, but introducing CT states leads to an even more significant reduction of over 0.15 eV. Furthermore, the CT states significantly increase the optical gap’s standard deviation by over 0.05 eV. This is because the LE-CT couplings have the most significant impact on the S_1_ energy of PAH aggregates and are also very sensitive to intermolecular conformations, as revealed by previous computational studies of asphaltene dimers.^50^ In addition, we also examine the configuration coefficients of LE and CT states solved from the predicted Hamiltonian and found an increase of CT component in the aggregate S_1_ state (Figure S17), demonstrating the significant influence of LE-CT state coupling on the aggregate’s lowest excited states.

Currently, our studies are confined to the homogeneous aggregates whose monomers’ S_0_-S_1_ transition is mostly HOMO–LUMO transition. Because the strong correlation between LE-CT couplings and CT-CT couplings only holds for pure HOMO–LUMO excitations^63^ (Text S3 for explanation). Such limitations hinder our model from predicting the CT-CT couplings from LE-CT couplings involving impure LE states under TeraChem’s exciton model framework. Another limitation is the error caused by configuration and orbital delocalization.^29^ The limited monomer LE states in the ML Hamiltonians is one of the major factors of this discrepancy, but it cannot be fully eliminated for ML models trained against fragment-based approaches. This may be addressed by predicting the Hamiltonian generated from eigenstate-based approaches such as MS-FED-FCD. Their Hamiltonian matrices are unitarily transformed from the diagonal matrices of the system’s adiabatic state energies, so their eigenvalues match the all-electron TDDFT. However, the off-diagonal couplings will rely on the chemical environment around the corresponding dimer so additional environmental embedding is needed. Additionally, as the excited state gradient can also be evaluated utilizing NN’s autodifferentiation, it would be possible to conduct ML-based excited state dynamics for large PAH assemblies if the above limitations have been addressed.

In summary, we presented a novel approach combining machine learning techniques with the Frenkel exciton model to enable optical property prediction for different-sized molecular assemblies. Our model was rigorously evaluated on perylene and tetracene aggregates, demonstrating its effective transferability to larger aggregates. By applying this model, we investigated the optical gap of nanosized molecular aggregates and explained their quantum-dot-like optical gap size dependency. Future research will focus on including more monomer LE states and surrounding environments, together with extending the transferability of the excited state potential energy surface (PES) to different PAH monomers, facilitating the prediction of heteroaggregates.

## Computational Details

*Data Sets and Calculations*. For perylene aggregate data set generation, we first generated a 50 ns classical molecular dynamic (MD) simulation trajectory of an amorphous condensed perylene system containing 400 monomers with Amber^85^ and AmberTools,^86^ with detailed MD simulation and conformation sampling procedure given in Text S8. With the MD trajectory, we curated the COM4A and NST5A subset of the dimer set PrDim. The two subsets were extracted from the trajectory based on different criteria: COM4A selected dimers with all atoms in monomer A to be within 4 Å of monomer B’s center of mass (COM),^57^ and NST5A selected the dimers with the shortest pairwise distance between the two monomers to be less than 5 Å. COM4A and NST5A were further refined to contain 8000 dimers each by sampling with the furthest point sampling (FPS) algorithm.

The SEP5A subset for PrDim was generated by randomly adding an extra 5–8 Å to the COMs separation of NST5A’s dimer structures. Corresponding Frenkel Hamiltonians are built with approximations by reusing the NST5A set’s exciton model results due to their identical monomer structures. Specifically, the diagonal LE state energies are approximated as E_LE(gas)_^A(1)^ for the corresponding entries in the NST5A set, as the Δ*Ĥ*_BA_ terms in text Eq. 2 are negligible for distantly separated dimers (Figure S3). The diagonal CT state energies were calculated based on
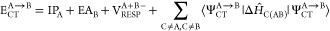
9where IP_A_ is monomer A’s ionized potential, EA_B_ is monomer B’s electron affinity, and V_RESP_^A+B–^ is the intermolecular Coulombic interaction calculated by the RESP charges on cationic A and anionic B. All terms in Eq. 9 are readily available in TeraChem’s output files in the NST5A set. The LE-LE couplings are estimated by the TrESP approach^61^ using Multiwfn^87^ based on the electrostatic potentials of each monomer’s S_1_ transition density, which was obtained from the computed monomer wave function in NST5A. The LE-CT couplings are set to 0 as they are in the order of 10^–5^ meV for separated dimers.^51^ The three subset forms the PrDim training set with 24000 dimer structures. Our approximations Hamiltonian terms of SEP5A were proven to be very accurate (*R*^2^ > 0.99) compared to ab initio exciton model calculations and significantly saved computation time (Figures S18 and S19). Using the same procedure, we curated the tetracene dimer training set TtDim containing 24,000 dimers.

To test the out-of-sample performance of the ML model on larger aggregates, we separately performed MD simulations to extract the out-of-sample test sets, oPrDim (500 dimers), oPrTri (500 trimers), and oPrTet (500 tetramers) for perylene, and oTtDim (500 dimers), oTtTri (500 trimers), and oTtTet (500 tetramers) for tetracene. Details about the curation of out-of-sample sets are available in Text S9.

All DFT, ab initio exciton model, and full electron TDDFT calculations in this work were performed with the GPU-accelerated QM package TeraChem^55^ at the wB97X-D3^88^/6-31G* level.^89,90^ The range-separated hybrid (RSH) DFT functional, wB97X-D3, was used because of its correct 1/r asymptotic behavior for CT states, making the exciton model result comparable to the full-electron TDDFT.^24^ To curate training data for ML, reference Frenkel Hamiltonians were generated for both PrDim and TtDim set, where the COM4A and NST5A subsets were calculated by the ab initio exciton model with 3 LE states per monomer and 2 CT states per monomer pair. The SEP5A subset, however, was generated with approximations by reusing NST5A results and only includes 1 LE state per monomer. The ab initio exciton model calculations and all-electron TDDFT were performed for the out-of-sample sets (oPrDim, oPrTri, oPrTet, oTtDim, oTtTri, and oTtTet) under the same theory level.

The optimized reference geometry and reference wave function of both perylene and tetracene, together with the cation and anion atomic RESP charges, are obtained with the same theory level and TeraChem’s default parameters. Their S_0_-S_1_ TrESP charges are fitted with the same approach for the SEP5A set.

*ML Model Training*. A modified TorchANI architecture was used to predict the dimer Frenkel Hamiltonian matrix elements, where each type of matrix element *E*^*Y*^ are evaluated by corresponding NN with their augmentations *Ẽ*^*Y*^. As TorchANI is an atomic NN, *Ẽ*^*Y*^ was decomposed to each atom’s contribution, *Ẽ*_*i*_^*Y*^, to augment that atom’s AEV. All ML models were trained with dimer data sets only, i.e., PrDim for the perylene models and TtDim for the tetracene models. The PrDim (or TtDim) set was split with a random 80% train/20% test split, where the training set was used for training the ML models with fixed hyperparameters. All models were built and trained with PyTorch,^91^ with the AEV computed by the “AEVComputer” function in the TorchANI package (hyperparameters listed in Table S4).^58^ Each model was trained for 500 epochs with batch optimization using a batch size of 64. The Adam optimizer^92^ was used with an exponential learning rate scheduler that decreases the learning rate from 1.0e-3 to 1.0e-6. The training loss is computed as the summation of the Mean squared error (MSE) for all individual matrix elements.

## Data Availability

Generated data sets for both perylene and tetracene, trained ML models, codes for training the ML models, codes for predicting the Hamiltonian of large aggregates, and the code for assessing the Hamiltonian with the couplings evaluated by analytical methods are available at https://doi.org/10.6084/m9.figshare.28012238.v1.

## Supporting Information Available

Impact of the second term in eq 3 on the perylene and tetracene trimer’s excited state energy, description and illustration of the architecture of our model, scheme of computing the approximations for different matrix terms, method for decomposing the approximations into atomic contributions, accuracy of the model on tetracene dimers’ Hamiltonian terms, proof of the relationship between CT-CT coupling and LE-CT couplings, accuracy of approximating CT-CT coupling by LE-CT couplings for both perylene and tetracene trimers, further investigation and discussion of the factor between CT-CT couplings and LE-CT couplings, impact of the LE state number on the exciton model’s accuracy, assessment of the efficiency and accuracy when evaluating the coupling elements by analytical methods, scheme for evaluating the perylene oscillator strength, distribution of MD sampled perylene monomer S_0_-S_1_ TDM with the reference monomer TDM, impact of LE state numbers on oscillator strength discrepancy, accuracy of the model on larger perylene aggregates, procedure of MD simulation and conformation sampling for gas-phase perylene nanoaggregates of up to 50 monomers, explanation of the trend of the optical gap without any coupling, LE and CT state components of the S_1_ state of different aggregates, detailed generation procedure of the PrDim and TtDim data set, approximation quality of different Hamiltonian terms for the SEP5A subset in PrDim and TtDim, detailed generation procedure of the OOS data sets, and hyperparameters of torchani.AEVComputer (PDF)
Transparent Peer Review report available (PDF)
