# Supplementary material for: Size-Transferable Prediction of Excited State Properties for Molecular Assemblies with a Machine Learning Exciton Model
Source: J Phys Chem Lett. 2025 Mar 3;16(10):2541–52. doi: 10.1021/acs.jpclett.4c03548 (PMC11912531; doi:10.1021/acs.jpclett.4c03548)
Supplement: Supplementary file 1 — jz4c03548_si_001.pdf [file jz4c03548_si_001.pdf]

Supporting Information for
Size-transferable prediction of excited state properties for molecular assemblies with machine-learned exciton model

Fangning Ren, Xu Chen, Fang Liu*

Department of Chemistry, Emory University, Atlanta, Georgia, 30322

Table of Contents

Figure S1. Impact of the second term in Eq. (3) on perylene trimer's excited state energy. 2

Figure S2. Impact of the second term in Eq. (3) on tetracene trimer's excited state energy. 3

Figure S3. The detailed architecture and workflow of our model. 4

Text S1. The model architecture. 4

Figure S4. Schematics for computing approximations. 6

Text S2. Methods for decomposing the approximations into atomic contribution. 6

Table S1. The accuracy of the ML exciton model on different Hamiltonian terms of tetracene dimers. 7

Text S3. The relationship between CT-CT coupling and LE-CT coupling. 8

Figure S5. LE-CT couplings versus CT-CT couplings of perylene trimers. 9

Figure S6. LE-CT couplings versus CT-CT couplings of tetracene trimers. 10

Text S4. Investigation of the correlation between LE-CT and CT-CT couplings. 10

Figure S7. $\boldsymbol{c_e}$ and $\boldsymbol{c_h}$ with different $\boldsymbol{c_{HF}}$ and $\boldsymbol{\omega}$. 11

Table S2. Correlation between LE-CT and CT-CT couplings for various DFT functionals and HF. Entries with "--" 11

Figure S8. The impact of LE state numbers on the exciton model's accuracy. 12

Text S5. Comparison of the ML-Hamiltonian with TrESP and AOM. 12

Figure S9. The OOS test set error of the ML exciton model with couplings evaluated by analytical methods. 13

Table S3. Timings for the ML-exciton model for different OOS datasets with coupling evaluated by TrESP and overlap integrals. 14

Figure S10. The workflow for evaluating the perylene oscillator strength. 14

Figure S11. Distribution of MD sampled perylene monomer S_0-S_1 transition dipole moment (TDM) magnitude with the reference TDM magnitude. 15

Figure S12. Impact of LE state numbers on oscillator strength discrepancy. 15

Text S6. The procedure of MD simulation and conformation sampling for gas-phase perylene nanoaggregates. 15

* Electronic mail: fang.liu@emory.edu

Figure S13. Aggregate optical gap with empirically adjusted LE energy. ... 16
Figure S14. Accuracy of the model on larger perylene aggregates. ... 17
Text S7. Explanation of the trend of the optical gap without any coupling. ... 17
Figure S15. Perylene monomer's S_0-S_1 excitation energy distribution. ... 18
Figure S16. Simulated and model-predicted optical gaps when ignoring all couplings. ... 19
Figure S17. LE and CT state components of different aggregate's S_1 state. ... 19
Text S8. Detailed procedure of geniting the COM4A and NST5A subsets in the PrDim and TtDim datasets. ... 19
Figure S18. The approximation quality for the tested 100 separate perylene dimers. ... 20
Figure S19. The approximation quality for the tested 100 separated tetracene dimers. ... 21
Text S9. Detailed generation procedure for the out-of-sample datasets. ... 21
Table S4. The hyperparameters of torchani.AEVComputer. ... 21

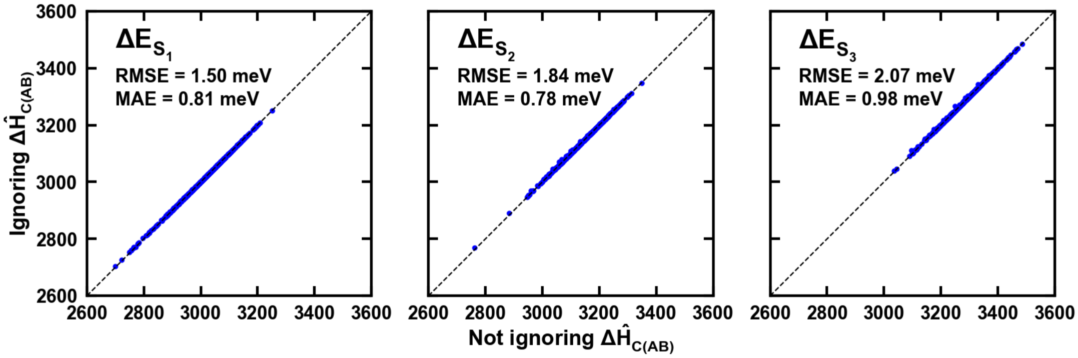

Figure S1. Impact of the second term in Eq. (3) on perylene trimer's excited state energy. All units are in meV. All exciton model calculations considered 5 LE states per monomer. For each CT state involving monomers A and B, extract dimer AB from the trimer and compute the dimer CT energy, then replace the aggregate CT energy with the dimer CT energy.

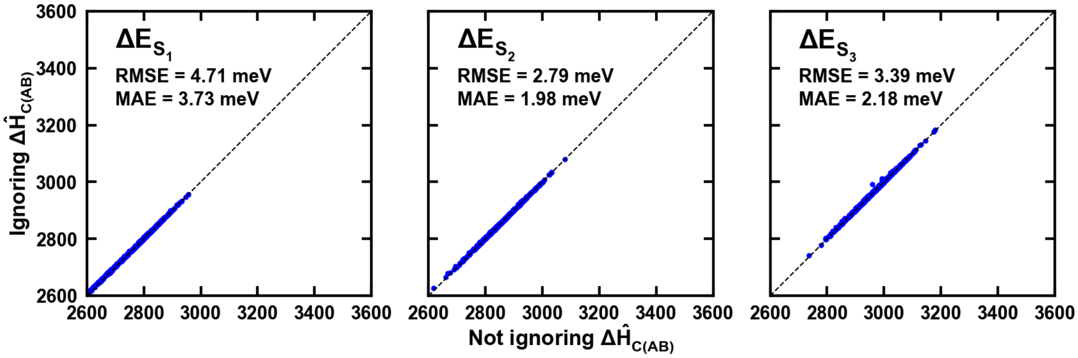

Figure S2. Impact of the second term in Eq. (3) on tetracene trimer's excited state energy. All units are in meV. All exciton model calculations considered 5 LE states per monomer. For each CT state involving monomers A and B, extract dimer AB from the trimer, compute the dimer CT energy, and then replace the aggregate CT energy with the dimer CT energy.

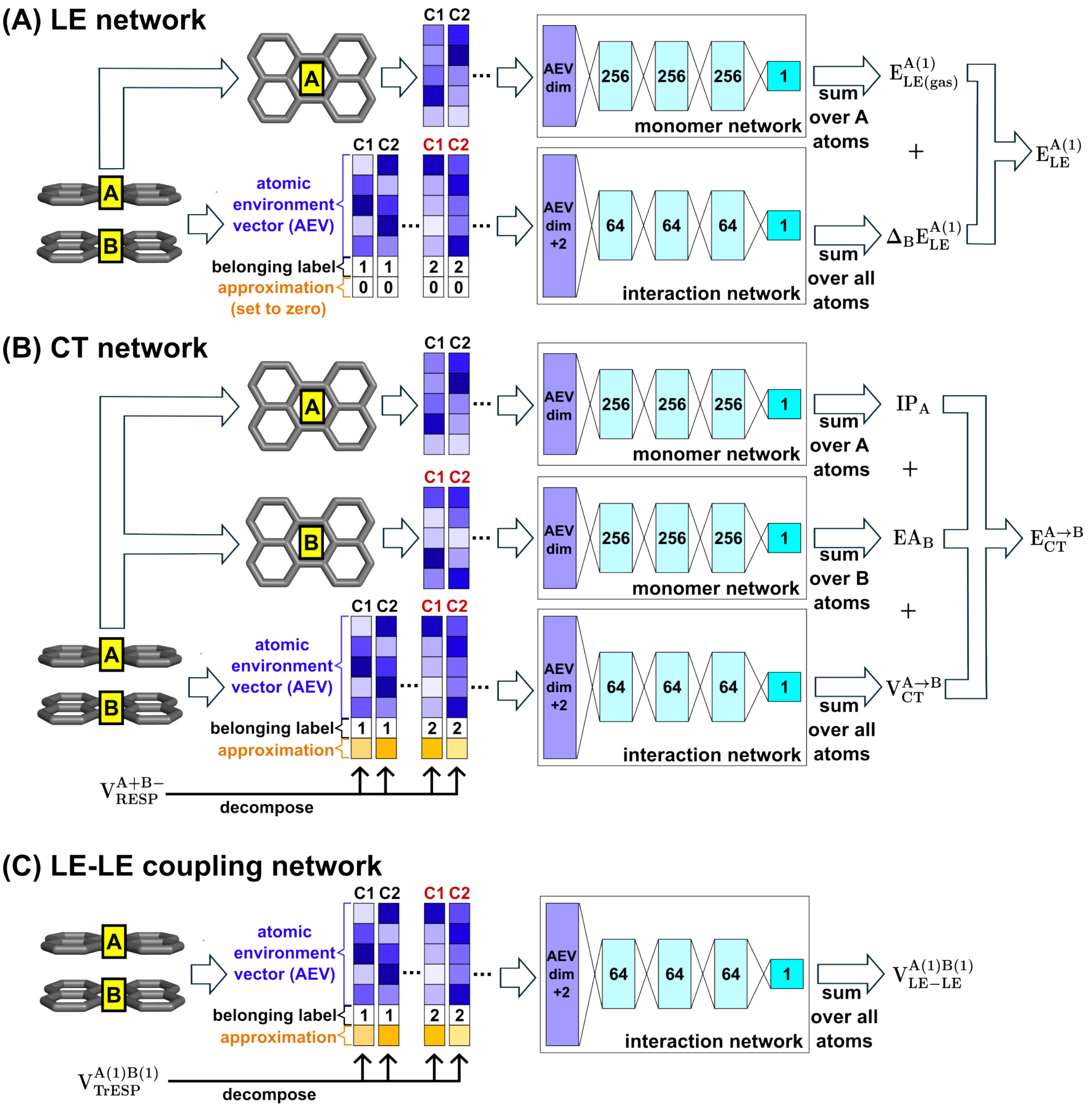

Figure S3. The detailed architecture and workflow of our model. (A) LE model ; (B) CT model ; (C) LE-LE coupling model. The hole coupling model and electron coupling model share the same architecture as the LE-LE coupling model but do not use S^{AB}_{HOMO} and S^{AB}_{LUMO} instead of $V^{A(1)B(1)}_{\mathrm{TrESP}}$ as approximation respectively.

Text S1. The model architecture.

We trained 5 different models to predict the different Hamiltonian terms. Namely, the LE network, the CT network, the LE-LE coupling network, the hole coupling network, and the electron coupling network. Each network may involve a single or multiple neuron networks atomic environment vectors (AEV) as their input, which are computed by the Python function "torchani.AEVComputer" from the TorchANI package.

a) **The LE network**. The LE network is responsible for predicting $E_{LE}^{A(1)}$ involves 2 different networks: a monomer network and an interaction network.

   i) *The monomer network.* This NN will output $E_{LE(gas)}^{A(1)}$. It is a full-connected neuron network (NN) with three hidden layers with 256 neurons each. Between each layer, the Continuously Differentiable Exponential Linear Units (CELU) activation function is used to ensure differentiability. Only AEVs for atoms on A are computed when ignoring the atom on the other monomer and are not concatenated with approximations or belonging labels before forward propagation.

   ii) *The interaction network*. This NN will output $\Delta_B E_{LE(gas)}^{A(1)} = \left\langle \Psi_{LE(gas)}^{A(1)} \middle| \Delta\hat{H}_{BA} \middle| \Psi_{LE(gas)}^{A(1)} \right\rangle$ shares the same number of hidden layers and activation function with the monomer network, but has 64 neurons in each hidden layer to prevent overfitting. AEVs for the interaction network consider all atoms in the dimers and are concatenated with belonging labels and approximations. In the LE network, these approximations are set to zero.

   iii) *Result aggregation*. The final $E_{LE}^{A(1)}$ are computed according to Eq. (1). When predicting dimers, only $\Delta_B E_{LE(gas)}^{A(1)}$ will be added to account for the impact of monomer B; when predicting larger aggregates with monomer A, B, C…, then each dimer pair involving monomer A would be passed through the interaction network to compute their contribution on A, and they were added to $E_{LE(gas)}^{A(1)}$ to obtain the final result.

b) **The CT network**. The CT network is responsible for predicting CT state energies such as $E_{CT}^{A\to B}$ involves 3 different networks: two monomer networks output the monomeric ionization potential (I.P.) and electron affinity (E.A.), and an interaction network predicts the interaction between the monomers.

   i) The monomer network for I.P. This NN takes the AEV of monomer A as input and outputs its ionization potential (IP_A). The AEV here also ignores the other monomer B, and this NN shares the identical architecture with the LE network's monomer network.

   ii) The monomer network for E.A. This NN takes the AEV of monomer B as input and outputs its electron affinity (EA_B). The AEV here also ignores the other monomer A, and this NN shares the identical architecture with the LE network's monomer network.

   iii) The interaction network. This NN takes the AEV of all atoms in the dimer as the input and predicts the interaction energy between cationic A and anionic B ($V_{CT}^{A\to B}$). The belonging label is used, while the Coulombic interaction with reference monomer atomic RESP charges (V_{RESP}^{A+B-}) were calculated and decomposed into atomic contributions and concatenated with the AEV.

   iv) *Result aggregation*. The final $E_{CT}^{A\to B}$ is computed by Eq. (3) but ignores the last term $\left\langle \Psi_{CT}^{A\to B} \middle| \Delta\hat{H}_{C(AB)} \middle| \Psi_{CT}^{A\to B} \right\rangle$ for larger aggregates.

c) **The LE-LE coupling network, the hole coupling network, and the electron coupling network.** These networks have only one neuron network that shares an identical architecture with the interaction network in the LE and CT networks. The only difference

is the approximation it used. For the LE-LE coupling network, the $V_{\text{TrESP}}^{\text{A(1)B(1)}}$ is used, while use $S_{\text{HOMO}}^{\text{AB}}$ and $S_{\text{LUMO}}^{\text{AB}}$ for hole coupling and electron coupling respectively.

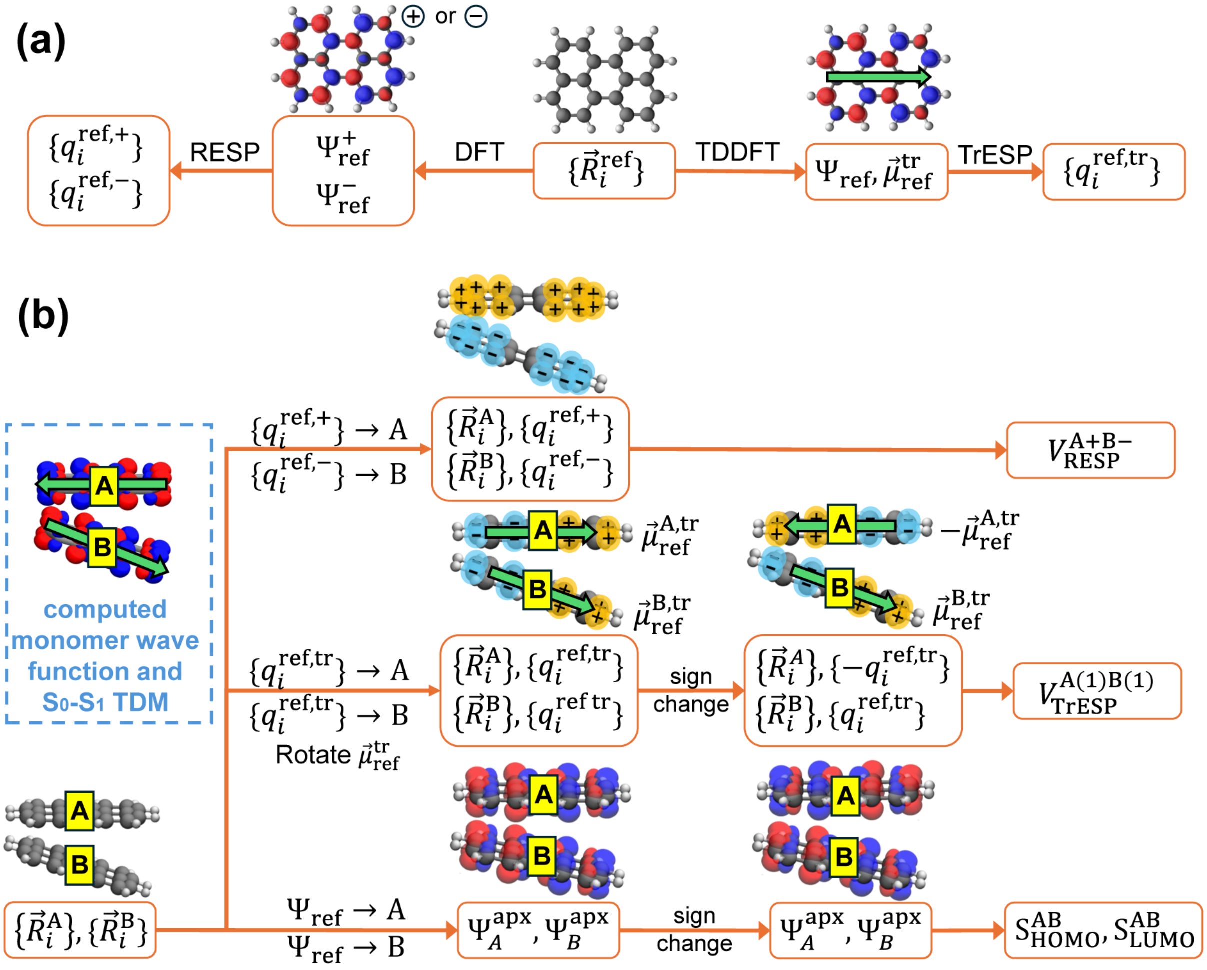

Figure S4. Schematics for computing approximations. (a) Generation of the reference monomer wave functions and corresponding RESP and TrESP atomic charges. These processes have been executed only once; (b) The process of constructing all approximations for a dimer entry with monomer wave functions computed by TeraChem's exciton model.

Text S2. Methods for decomposing the approximations into atomic contribution.

1) **The Coulombic interaction between cationic A and anionic B ($V_{\text{RESP}}^{\text{A+B−}}$).** $V_{\text{RESP}}^{\text{A+B−}}$ can be expressed as:

$$V_{\text{RESP}}^{A+B-} = \sum_i \sum_j q_i^{A+} q_j^{B-} / r_{ij} \quad (S1)$$

Here q_i^{A+} is the fitted RESP charge of the ith atom on cationic monomer A and q_j^{B-} is the fitted RESP charge of the jth atom on anionic monomer B. All RESP atomic charges are fitted at the reference geometry and then assigned to the monomers in a dimer entry with

Multiwfn. Then, we computed the contribution of the ith atom on monomer A to the total V_{RESP}^{A+B-} as:

$$V_{\text{RESP},i}^{A+B-} = \sum_j q_i^{A+} q_j^{B-} / r_{ij} \qquad (S2)$$

The contribution of the jth atom on monomer B as:

$$V_{\text{RESP},j}^{A+B-} = \sum_i q_i^{A+} q_j^{B-} / r_{ij} \qquad (S3)$$

2) **The Coulombic interaction between TrESP charges on A and B** ($\text{V}_{\text{TrESP}}^{\text{A(1)B(1)}}$). This term can be computed in the same way when replacing the q_i^{A+} and q_j^{B-} by the fitted TrESP charge from the S0-S1 transition density of the reference monomer structure. The TrESP charge fitting also utilized Multiwfn.

3) **The overlap integral between A and B's HOMO** ($\text{S}_{\text{HOMO}}^{\text{AB}}$). $\text{S}_{\text{HOMO}}^{\text{AB}}$ can be expressed as:

$$\text{S}_{\text{HOMO}}^{\text{AB}} = \sum_k \sum_l c_{\text{HOMO},k} c_{\text{HOMO},l} \langle \chi_k^A | \chi_l^B \rangle \qquad (S4)$$

Here $c_{\text{HOMO},k}$ and $c_{\text{HOMO},l}$ are the coefficients of the kth and lth Gaussian type orbital (GTO) of the HOMO, which is pre-computed at the reference geometry. Then, the reference wave function is translated and rotated to align with monomers A and B. The kth GTO after aligned to A is noted as χ_k^A and lth GTO on monomer B is noted as χ_l^B. The $\langle \chi_k^A | \chi_l^B \rangle$ is the overlap integral between the two GTOs.

Then, we computed the contribution of the ith atom on monomer A as:

$$\text{S}_{\text{HOMO},i}^{\text{AB}} = \sum_{k \text{ on atom } i} \sum_l c_{\text{HOMO},k} c_{\text{HOMO},l} \langle \chi_k^A | \chi_l^B \rangle \qquad (S5)$$

Here, the first summation only goes through the GTOs centered on the ith atom on monomer A. The contribution of the jth atom on monomer B can be computed in the same way when letting the second summation in Eq. (S4) only go through the GTOs centered on the jth atom on monomer B. The calculation of the overlap integrals between GTOs is conducted with our in-house Python code.

4) **The overlap integral between A and B's LUMO** ($\text{S}_{\text{LUMO}}^{\text{AB}}$).
$\text{S}_{\text{LUMO},i}^{\text{AB}}$ can be computed in the same way as $\text{S}_{\text{HOMO},i}^{\text{AB}}$ when replacing $c_{\text{HOMO},k}$ and $c_{\text{HOMO},l}$ by $c_{\text{LUMO},k}$ and $c_{\text{LUMO},l}$ in Eq. (S5).

Table S1. The accuracy of the ML exciton model on different Hamiltonian terms of tetracene dimers. All statistic variables are evaluated over all three datasets.

Term	Mean absolute error (meV)	R^2	Reference value standard deviation (meV)
$\text{E}_{\text{LE}}^{\text{A(1)}}$	6.53	0.993	95.63
$\text{E}_{\text{LE}}^{\text{B(1)}}$	6.52	0.993	95.94

$E_{CT}^{A\to B}$	9.95	1.000	628.65
$E_{CT}^{B\to A}$	10.29	1.000	635.68
$V_{LE-LE}^{A(1),B(1)}$	3.11	0.994	66.46
$V_{LE-CT}^{A(1),A\to B}$	5.04	0.992	94.36
$V_{LE-CT}^{A(1),B\to A}$	5.57	0.995	125.27
$V_{LE-CT}^{B(1),A\to B}$	5.82	0.994	124.02
$V_{LE-CT}^{B(1),B\to A}$	4.97	0.992	92.57

Text S3. The relationship between CT-CT coupling and LE-CT coupling.

Given monomer A, B, and C, we start from the "electron coupling" between two CT state $A \to B$ and $A \to C$ that can be expressed as:

$$V_{CT-CT}^{A\to B,A\to C} \approx \langle \Psi_{CT}^{A\to B} | \hat{H} | \Psi_{CT}^{A\to C} \rangle = f_{l_B l_C} \qquad (S6)$$

Here $\hat{H}$ is the exact Hamiltonian of dimer BC. This coupling belongs to the "electron coupling" regime, which means that when the dimer's electronic state changed from $\Psi_{CT}^{A\to B}$ to $\Psi_{CT}^{A\to C}$, it can be viewed as electron transferred from B to C.

And the corresponding LE-CT coupling between $\Psi_{LE}^{B(1)}$ and $\Psi_{CT}^{B\to C}$ is:

$$\begin{aligned} V_{LE-CT}^{B(1),B\to C} &= \left\langle \Psi_{LE}^{B(1)} \middle| \hat{H} \middle| \Psi_{CT}^{B\to C} \right\rangle \\ &= \textstyle\sum_{i_B a_B} c_{i_B a_B}^{(1)} \, [\delta_{i_B h_B} f_{a_B l_C} + 2(i_B a_B | h_B l_C) \\ &\quad - c_{HF}(i_B h_B | a_B l_C) - (1 - c_{HF})(i_B a_B | f_{xc} | h_B l_C) \end{aligned} \qquad (S7)$$

Here we utilized the molecular orbital basis of molecule B and C. h_B, h_C are the HOMO of B and C, while l_B, l_C are the LUMO of B and C respectively. $c_{i_B a_B}^{(1)}$ is the configuration interaction (CI) coefficient of the $i_B \to a_B$ transition in the first LE state of monomer B. $f_{a_B l_C}$ is the Fock matrix element of dimer AB under the monomer molecular orbital basis. $\delta_{i_B h_B}$ is the delta-function that equals 1 when $i_B = h_B$ and 0 otherwise. c_{HF} is the Hartree-Fock (HF) exchange partition in the DFT functional.

If the 1st singlet excited state is mostly a HOMO-LUMO transition, which means $c_{i_B a_B}^{(1)} \approx \pm 1$ for $i_B = h_B$ and $a_B = l_B$, and $c_{i_B a_B}^{(1)} \approx 0$ otherwise. Therefore, we have:

$$\begin{aligned} V_{LE-CT}^{B(1),B\to C} &= f_{h_B l_C} + 2(h_B l_B | h_B l_C) \\ &\quad - c_{HF}(h_B h_B | l_B l_C) - (1 - c_{HF})(h_B l_B | f_{xc} | h_B l_C) \end{aligned} \qquad (S8)$$

According to the exciton model article[1], the leading contribution of this term comes from the single electron term, which is the term involving $f_{h_B l_C}$. Therefore, we can approximate the LE-CT coupling as the CT-CT coupling:

$$V_{LE-CT}^{B(1),B\to C} \approx f_{l_B l_C} \approx V_{CT-CT}^{A\to B,A\to C} \qquad (S9)$$

The "hole coupling" $V_{CT-CT}^{C\to A,B\to A}$ between two CT state $B \to A$ and $C \to A$ can be computed as:

$$V_{CT-CT}^{C\to A,B\to A} = \langle \Psi_{CT}^{B\to A} | \hat{H} | \Psi_{CT}^{C\to A} \rangle \approx -f_{h_B h_C} \qquad (S10)$$

Here the "hole coupling" means when the electronic state changes from $\Psi_{CT}^{B\to A}$ to $\Psi_{CT}^{C\to A}$, it can be recognized as a hole transferred from B to C. The corresponding LE-CT coupling $V_{LE-CT}^{B(1),C\to B}$ can be computed as:

$$V_{LE-CT}^{B(1),C\to B} = \left\langle \Psi_{LE}^{B(1)} \middle| \hat{H} \middle| \Psi_{CT}^{C\to B} \right\rangle$$
$$= \sum_{i_B a_B} c_{i_B a_B}^{(1)} \left[-\delta_{a_B l_B} f_{i_B h_C} + 2(i_B a_B | h_C l_B) \right.$$
$$\left. - c_{HF}(i_B h_C | a_B l_B) - (1 - c_{HF})(i_B a_B | f_{xc} | h_C l_B) \right] \qquad (S11)$$

Using the same assumption with the electron coupling, we can easily get:

$$V_{LE-CT}^{B(1),C\to B} \approx -f_{h_B h_C} \approx V_{CT-CT}^{C\to A,B\to A} \qquad (S12)$$

This shows the "hole coupling" can still be approximated in the same way.

Testing of 100 perylene trimers (not appearing in the training set and test set) showed that there is a very strong linear correlation between the CT-CT coupling and the corresponding LE-CT coupling (R^2>0.99), but they differ by coefficient.

According to Figure S5 and **Figure S6**, for all "electron couplings" ($V_{CT-CT}^{A\to B,A\to C}$, $V_{CT-CT}^{B\to A,B\to C}$, $V_{CT-CT}^{C\to A,C\to B}$), they can be accurately approximated by multiplying 0.75 to the corresponding LE-CT couplings ($V_{LE-CT}^{B(1),B\to C}$, $V_{LE-CT}^{A(1),A\to C}$, $V_{LE-CT}^{C(1),C\to B}$, respectively). For all "hole couplings" ($V_{CT-CT}^{B\to A,C\to A}$, $V_{CT-CT}^{A\to B,C\to B}$, $V_{CT-CT}^{A\to C,B\to C}$), they can be accurately approximated by multiplying 1.20 by the corresponding LE-CT couplings ($V_{LE-CT}^{B(1),C\to B}$, $V_{LE-CT}^{A(1),C\to A}$, $V_{LE-CT}^{A(1),A\to B}$, respectively). The discussion about the factor can be seen in **Text S4**.

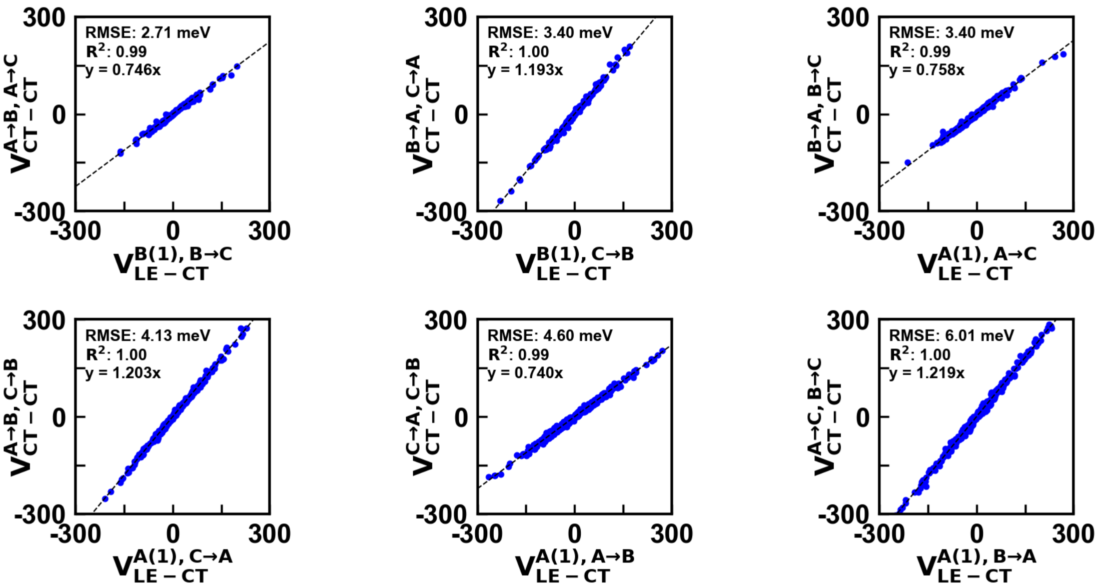

Figure S5. LE-CT couplings versus CT-CT couplings of perylene trimers. The accuracy is tested for 100 perylene trimers that are not included in the test set.

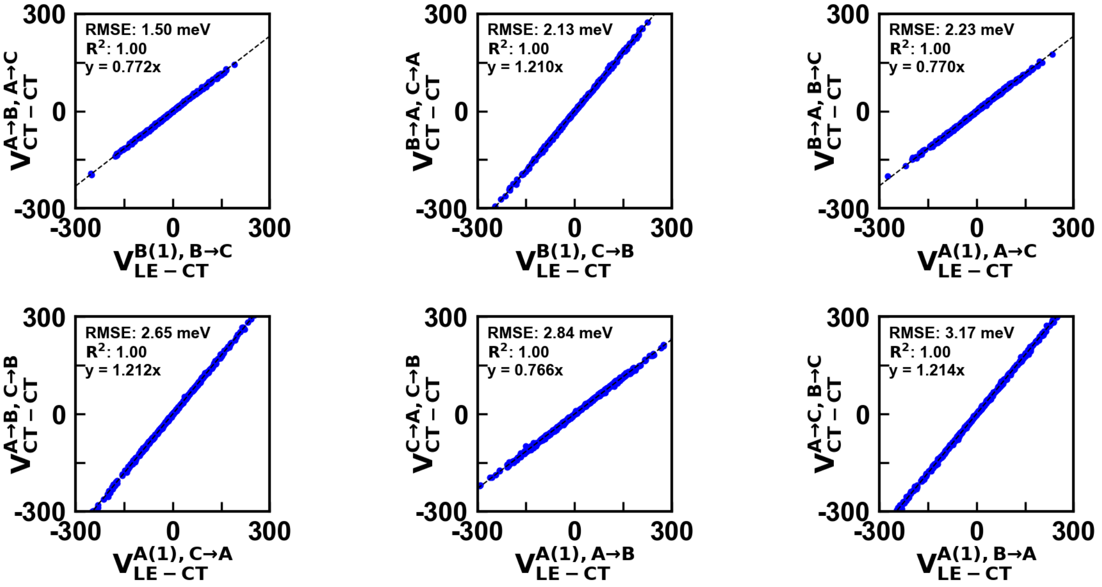

Figure S6. LE-CT couplings versus CT-CT couplings of tetracene trimers. The accuracy is tested for 100 tetracene trimers that are not included in the test set.

Text S4. Investigation of the correlation between LE-CT and CT-CT couplings.

Although strongly correlated, there still exists a factor between the two types of couplings, namely c_e and c_h. Then, the approximation should be expressed as:

$$V_{CT-CT}^{A\to B,A\to C} \approx c_e V_{LE-CT}^{B(1),B\to C}$$
$$V_{CT-CT}^{C\to A,B\to A} \approx c_h V_{LE-CT}^{B(1),C\to B} \qquad (S13)$$

This discrepancy can be only caused by the non-zero two-electron terms for the electron LE-CT coupling:

$$V_{LE-CT,2e}^{B(1),B\to C} = 2(h_B l_B|h_B l_C) - c_{HF}(h_B h_B|l_B l_C) - (1-c_{HF})(h_B l_B|f_{xc}|h_B l_C) \qquad (S14)$$

To investigate the origin of this factor, we changed the HF exchange factor c_{HF} in the RSH functional wB97X-D3. The default c_{HF} for its short-range (SR) and long-range (LR) parts are 0.2 and 0.8, respectively. We started from completely disabling the range-separation by setting the range-separation parameter ω to zero, followed by adjusting the SR c_{HF} from 0.0 to 1.0. $c_e(c_h)$ are obtained by extracting all electron (hole) LE-CT couplings together in trimer Hamiltonians, then fitting them with all electron (hole) CT-CT couplings in the same Hamiltonian. The same 100 perylene trimers were used to estimate the factor. As shown in **Figure S7**, c_e decreased from 1.07 to 0.72 and c_h increased from 1.00 to 1.21, indicating their sensitivity against the c_{HF}.

We also investigated the impact of the range-separated factor, w, on c_e and c_h. Here the SR and LR c_{HF} are set to their default value, but ω varies from 0.0 a.u.$^{-1}$ to 0.5 a.u.$^{-1}$. (**Figure S7**). Surprisingly, both c_e and c_h equals to 1.00 at the beginning, but gradually approaches that obtained when $c_{HF} = 1.0$, equivalent to the HF theory. This is because the interaction between PAH monomers is governed by long-range interactions, as when $\omega = 0.25$ a.u.$^{-1}$, the default ω in wB97X-D3, the LR exchange partition erf (ωr) is over 0.95 when 3.6 Å $< r <$ 4.0 Å, the typical intermolecular distance between face-to-face stacked PAH molecules.

To check whether this factor depends on the DFT functional f_{xc}, we evaluate the couplings with various DFT functionals (**Table S2**). The correlations are very strong ($R^2 \approx 1.00$) for all DFT functionals, while the deviation of c_e and c_h from 1 becomes serious when the functional has more HF exchange partition, but c_e and c_h does not rely on the specific DFT exchange and correlation functional itself. It is worth mentioning that c_e and c_h are very close to 1 for commonly used hybrid functionals without range separation, such as B3LYP and PBE0. Therefore, we believe that the existence of this parameter is most likely due to the implementation of the TeraChem Exciton Model itself. Since TeraChem's exciton model can predict the absorption spectra of large aggregates, we chose to accept this fact in the current work and include this parameter in our machine learning pipeline. In some other exciton model theoretical frameworks,[2] LE-CT and CT-CT couplings are defined to be strictly equal. This parameter may be different or not required for our machine learning method in these theoretical frameworks.

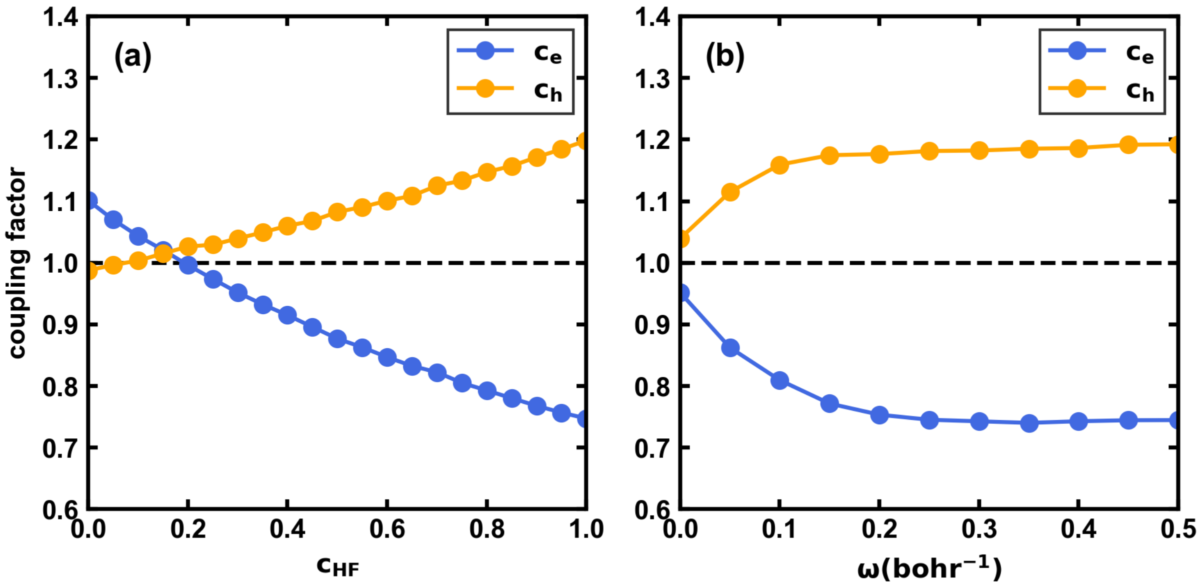

Figure S7. c_e and c_h with different c_{HF} and ω. (a) c_e and c_h evaluated at different SR c_{HF} with $\omega = 0$ bohr^{-1}; (b) c_e and c_h evaluated at different ω with SR $c_{HF} = 0.20$ and LR $c_{HF} = 0.80$.

Table S2. Correlation between LE-CT and CT-CT couplings for various DFT functionals and HF. Entries with "--" means this property does not apply to this functional. [a] "GGA" refers to "general gradient approximation functional", "Hybrid" refers to "Hybrid GGA functional", and "RSH" refers to "range separated hybrid functional".

functional	type [a]	SR c_{HF}	LR c_{HF}	ω (bohr^{-1})	c_e	c_h	R^2(electron)	R^2(hole)
BLYP	GGA	0.00	--	--	1.11	0.99	1.00	1.00
PBE	GGA	0.00	--	--	1.11	0.99	1.00	1.00
B3LYP	Hybrid	0.20	--	--	1.00	1.02	1.00	1.00
B97	Hybrid	0.20	--	--	1.00	1.02	1.00	1.00
PBE0	Hybrid	0.25	--	--	0.97	1.03	1.00	1.00
BHandHLYP	Hybrid	0.50	--	--	0.88	1.08	1.00	1.00
wB97	RSH	0.00	1.00	0.40	0.75	1.20	1.00	1.00

wB97X	RSH	0.16	0.84	0.30	0.75	1.20	1.00	1.00
CAM-B3LYP	RSH	0.19	0.46	0.33	0.84	1.11	1.00	1.00
wPBEh	RSH	0.20	0.80	0.20	0.75	1.20	1.00	1.00
wB97X-D3	RSH	0.20	0.80	0.25	0.75	1.20	1.00	1.00
HF	HF	1.00	--	--	0.72	1.22	1.00	1.00

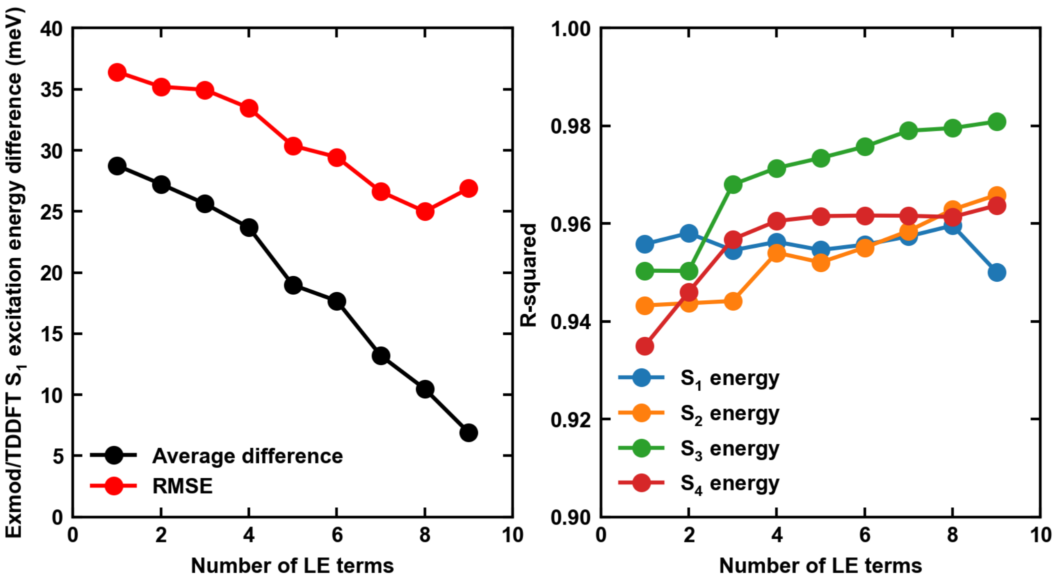

Figure S8. The impact of LE state numbers on the exciton model's accuracy. (left) The difference in the average of the S_1 energies of 500 dimers between the exciton model and TDDFT (black line and marker) and the average RMSE between individual entries (red line and marker). (right) R^2 between the first four excitation energies computed by the exciton model with different LE states and the all-electron TDDFT.

Text S5. Comparison of the ML-Hamiltonian with TrESP and AOM.

The following discussion focused on a homogeneous aggregate with N monomers. TrESP or AOM approaches require a converged excited state wavefunction for each monomer to accurately evaluate them, resulting in N QM calculations. As TeraChem's exciton model can evaluate all couplings after getting the wave function for all monomers, these approaches would have no significant efficiency advantages compared to the ab initio exciton model.

A simplified way is assuming the wave function of the monomer does not change with its configuration, resulting in using a set of frozen TrESP charges and wave function on each monomer to evaluate the couplings. These approaches avoided unnecessary QM calculations and have been applied in long-time non-adiabatic dynamics simulations.[3] However, such approaches are not yet available for estimating the LE and CT state energies and may introduce errors for contact systems such as PAH assemblies.[4] This results in additional errors without improving efficiency, as we will explain as follows.

When evaluating the Hamiltonian of an aggregate with N monomers one needs to consider $N(N-1)/2$ dimer pairs. If only the first LE states are considered, a single NN iteration with a given dimer pair will yield 2 LE energies, 2 CT energies, 1 LE-LE coupling, 2 hole couplings, and 2 electron couplings at once. These values except LE energy are unique. Therefore,

traversing all dimer pairs with $N(N-1)/2$ NN propagations can predict all the N LE energies, $N(N-1)$ CT state energies, $N(N-1)/2$ LE-LE couplings, $N(N-1)$ hole couplings, $N(N-1)$ electron couplings. Many of the $N(N-1)(N-2)$ CT-CT couplings are identical and can be accurately approximated by corresponding LE-CT couplings, resulting in negligible computational cost no matter which method is used. As predicting $N(N-1)$ CT state energies still require $N(N-1)/2$ NN propagations, using TrESP or AOM does not reduce the number of NN propagations, thus having identical efficiency with complete ML Hamiltonian.

We also evaluated the accuracy of analytically estimated couplings on the OOS datasets. The TrESP charge and AOM approach based on a frozen wave function coincided with the analytic approximations we used in training our model, where a reference wave function is utilized to compute these quantities. Therefore, inspired by the AOM approach,[5] we evaluate the off-diagonal elements E^Y based on their approximations $\tilde{E}^Y$:

$$E^Y = C\tilde{E}^Y \qquad (S15)$$

where C is a constant that needs to be learned from the training set. The diagonal elements are still evaluated by the corresponding ML-model to reduce the computational overhead. To reduce computational overhead as much as possible, *NN modules for evaluating couplings are disabled*. The accuracy of this combined method is illustrated in **Figure S9**, where the MAE of all OOS-datasets is increased by around 5 meV. In addition, the time for evaluating these results does not change significantly, (**Table S3**), indicating the ML model has achieved a very good balance between accuracy and efficiency in our case.

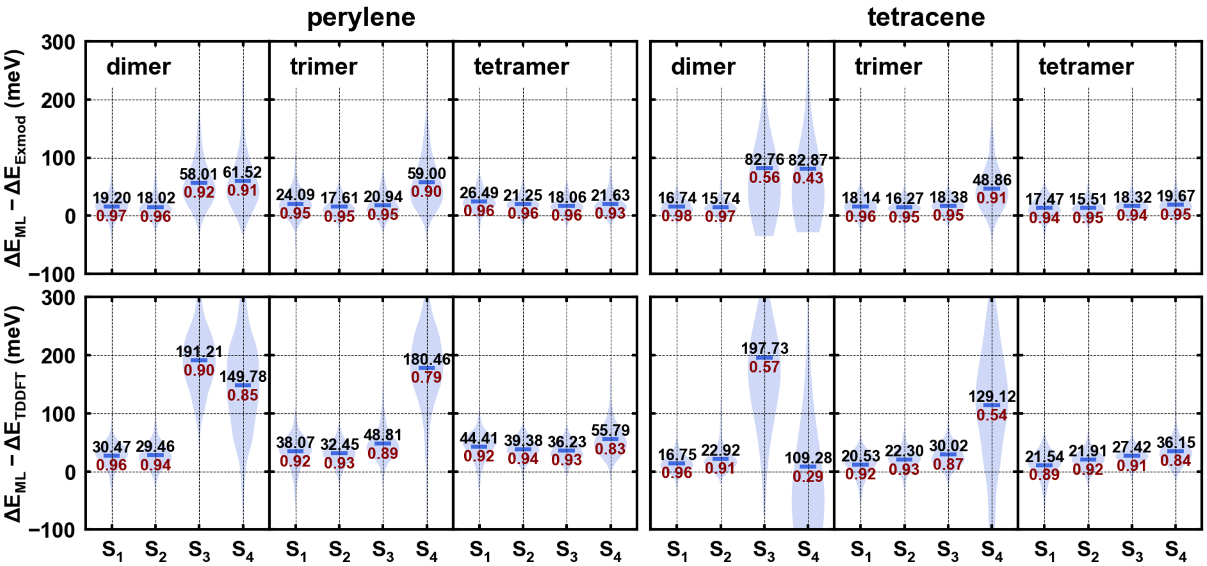

Figure S9. The OOS test set error of the ML exciton model with couplings evaluated by analytical methods. See the caption of **Figure 4** for a detailed explanation.

Table S3. Timings for the ML-exciton model for different OOS datasets with coupling evaluated by TrESP and overlap integrals. Each OOS dataset contains 500 aggregate structures. The ML timings are measured with *an* Intel(R) Xeon(R) Silver 4210R CPU and one Nvidia RTX A4000 GPU, while the timing for QM calculations *is* the total GPU hours used with the same hardware.

Dataset	Number of monomers	TrESP & RESP charge interaction time (s)	overlap integral time (s)	NN propagation time (s)	Total time (s)
oPrDim	2	0.05	6.45	0.63	7.13
oPrTri	3	0.11	9.26	1.03	10.40
oPrTet	4	0.24	14.05	2.02	16.31
oTtDim	2	0.04	5.88	0.82	6.74
oTtTri	3	0.11	8.00	1.03	9.14
oTtTet	4	0.23	11.82	2.04	14.09

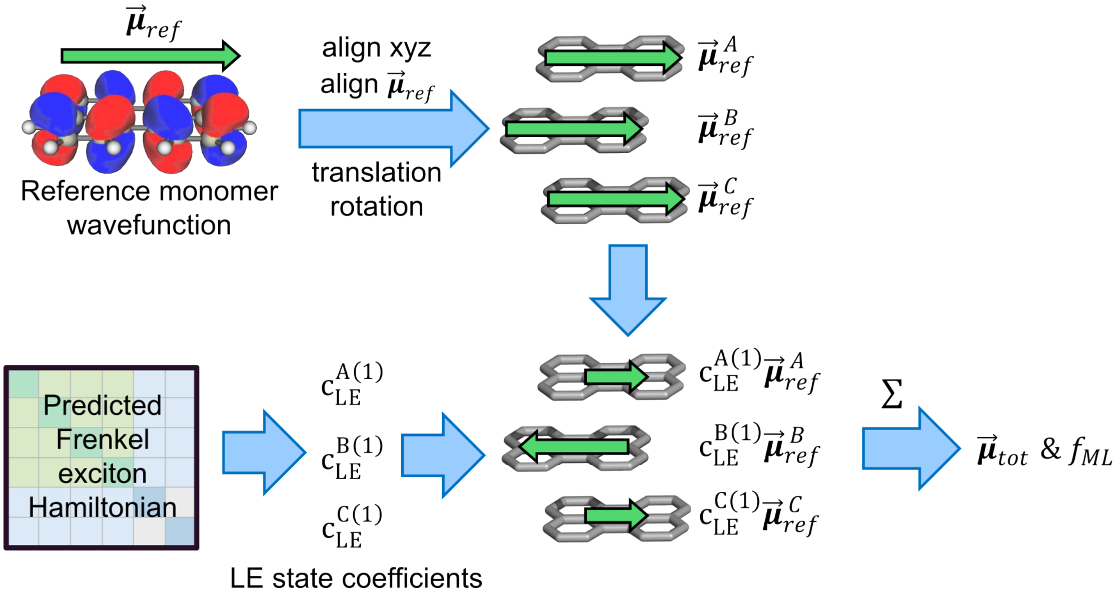

Figure S10. The workflow for evaluating the perylene oscillator strength.

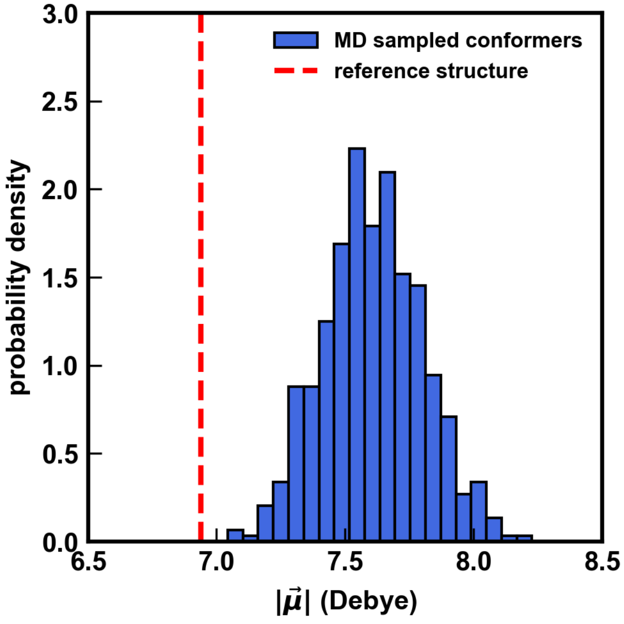

Figure S11. Distribution of MD sampled perylene monomer S_0-S_1 transition dipole moment (TDM) magnitude with the reference TDM magnitude.

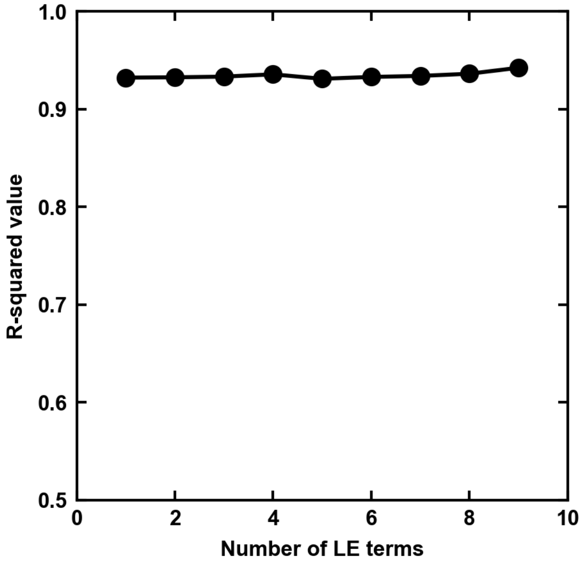

Figure S12. Impact of LE state numbers on oscillator strength discrepancy. The correlation between the S_1 oscillator strength evaluated by the exciton model and TDDFT over 500 perylene trimers.

Text S6. The procedure of MD simulation and conformation sampling for gas-phase perylene nanoaggregates.

1. **Initial structure generation.** The perylene monomer's forcefield is generated in the same way for the amorphous perylene system. When generating the initial conformation of

nanoaggregates containing 1 monomer to 50 monomers, we use the Packmol program to pack the corresponding number of perylenes inside a confined cube. The cube's size is determined by making the overall density equal to 90% of the density of α-perylene crystals. Then the AMBER topology and coordinate file are generated by tleap as well.

2. **MD trajectory generation.** All MD simulations use the time step 2fs, with a nonbonded cutoff of 8.0 Å. No periodic boundary conditions are used. We first performed 1000 steps of energy minimization with an energy tolerance 1 kcal/mol. Then performed 2ps heating simulation to heat the system to 300K using Langevin dynamics with the friction parameter γ=2.0, followed by a 2ps NVE equilibrium simulation. The 5ns production run is performed under the NVT ensemble under 300K with the snapshot interval of 5000 steps (10ps), resulting in 500 conformers. No further de-duplication is completed.

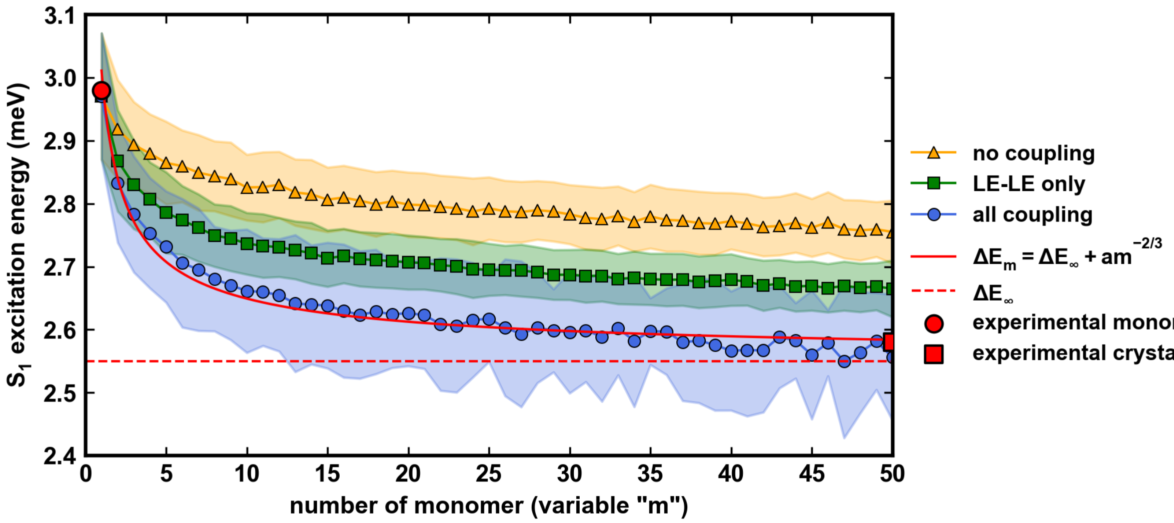

Figure S13. Aggregate optical gap with empirically adjusted LE energy. The color-coding is identical to **Figure 6** except the experimental absorption maximum of perylene monomer and α-crystal are marked as round and square red dots, respectively.

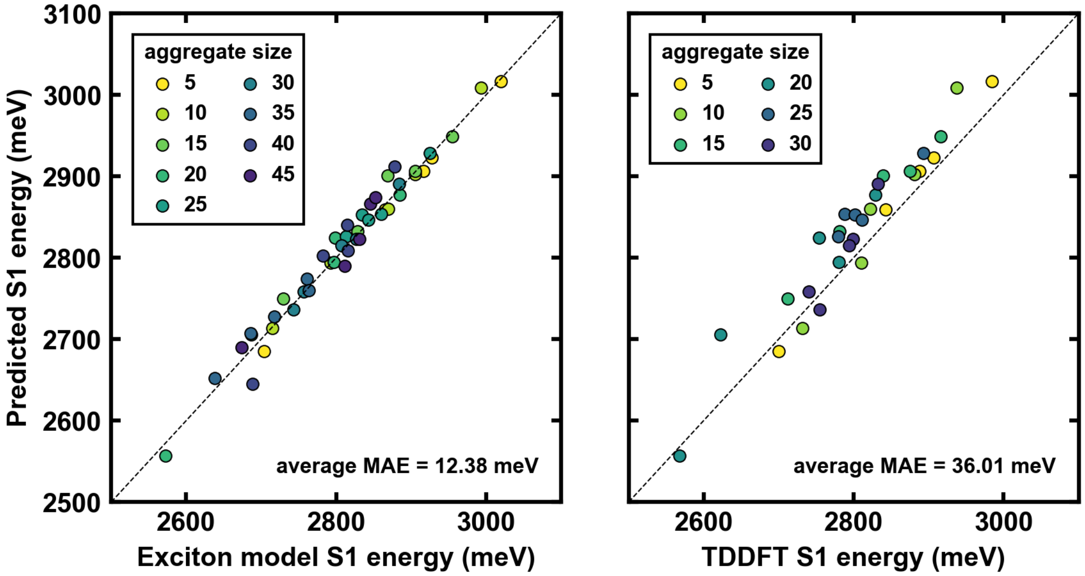

Figure S14. Accuracy of the model on larger perylene aggregates. The all-electron TDDFT calculation of nanoaggregates over 30 monomers can't be conducted due to memory overflow.

Text S7. Explanation of the trend of the optical gap without any coupling.

For an aggregate with m monomers, if all couplings are ignored, its Frenkel Hamiltonian matrix degenerates into a diagonal matrix. For the ML-predicted Hamiltonian that only includes the first LE state of each monomer, it can be expressed as:

$$\mathbf{H}_{\text{no coupling}} = \begin{bmatrix} E_{\text{LE}}^{1(1)} & 0 & \cdots & 0 \\ 0 & E_{\text{LE}}^{2(1)} & \cdots & 0 \\ \vdots & \vdots & \ddots & \vdots \\ 0 & 0 & \cdots & E_{\text{LE}}^{m(1)} \end{bmatrix} \quad (S16)$$

Here $E_{LE}^{1(1)}, E_{LE}^{2(1)}, \cdots E_{LE}^{m(1)}$ is the LE state energy of each monomer. As the eigenvalues of a diagonal matrix are its diagonal elements themselves. When getting the optical gap of the no-coupling aggregate as the smallest eigenvalue of $\mathbf{H}_{no\ coupling}$, we just need to get the smallest diagonal element over m LE state energies.

$$\Delta E(m) = \min(E_{\text{LE}}^{1(1)}, E_{\text{LE}}^{2(1)}, \cdots E_{\text{LE}}^{m(1)}) \quad (S17)$$

When all couplings are neglected, the M monomers are infinitely separated without any intermolecular interaction. So conformational distribution will not affect each other. Therefore, the first excited state energy of m monomers (the first LE state energy), can be considered as m independent and identically distributed (i.i.d.) random variables and the average optical gap can be computed by the expectation of the minimum value of m i.i.d. random variables.

$$\Delta\bar{E}(m) = \langle \min\left(E_{\mathrm{LE}}^{1(1)}, E_{\mathrm{LE}}^{2(1)}, \cdots E_{\mathrm{LE}}^{m(1)}\right)\rangle \quad (S18)$$

Here we confirm the trend caused by statistical principles by doing a numerical experiment. We examined the distribution of the S_0-S_1 excitation energy (first LE state energy) from 32000 perylene monomer conformations from the 16000 entries in the NST5A and COM4A datasets. After fitting with a Gaussian function, we found that the probability density distribution of its S_0-S_1 excitation energy perfectly conforms to a normal distribution centered at 3.181 eV with a standard deviation of 0.096 eV (Figure S15).

Then, for each m ranged from 1 to 50, we generated 500 arrays with each number sampled from $N(\mu, \sigma^2)$ with $\mu = 3.181$ and $\sigma = 0.096$, as m simulated LE state energies. Then the minimum value of each array was calculated as the simulated optical gap. The average value and standard deviation were calculated based on the 500 simulated optical gaps and compared with the ML-predicted optical gap when ignoring all couplings.

As shown in **Figure S16**, the simulated result nearly perfectly reproduced both the average value and standard deviation, indicating that the observed trend is mainly caused by statistical reasons. The small difference between them may be due to the impact of other monomers on the conformation and energy distribution of individual monomers.

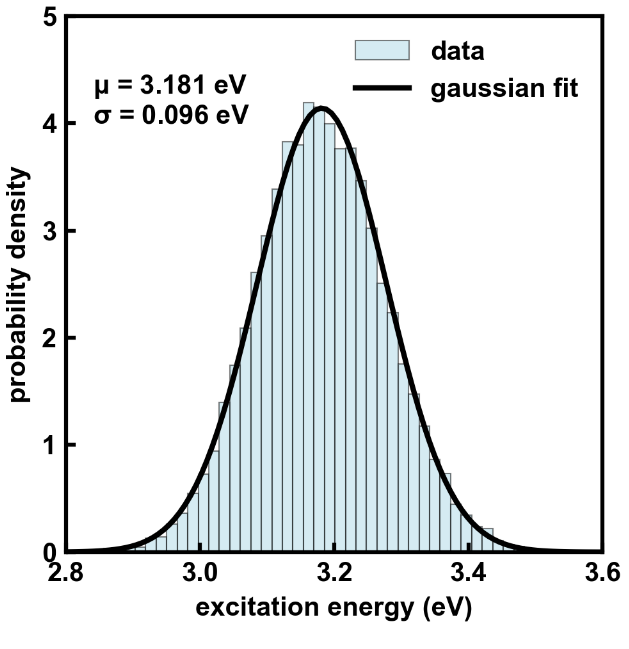

Figure S15. Perylene monomer's S_0-S_1 excitation energy distribution. Evaluated from all 32000 monomer conformations from 16000 entries in the NST5A and COM4A datasets. The fitted average (μ) and standard deviation (σ) are shown in the upper left.

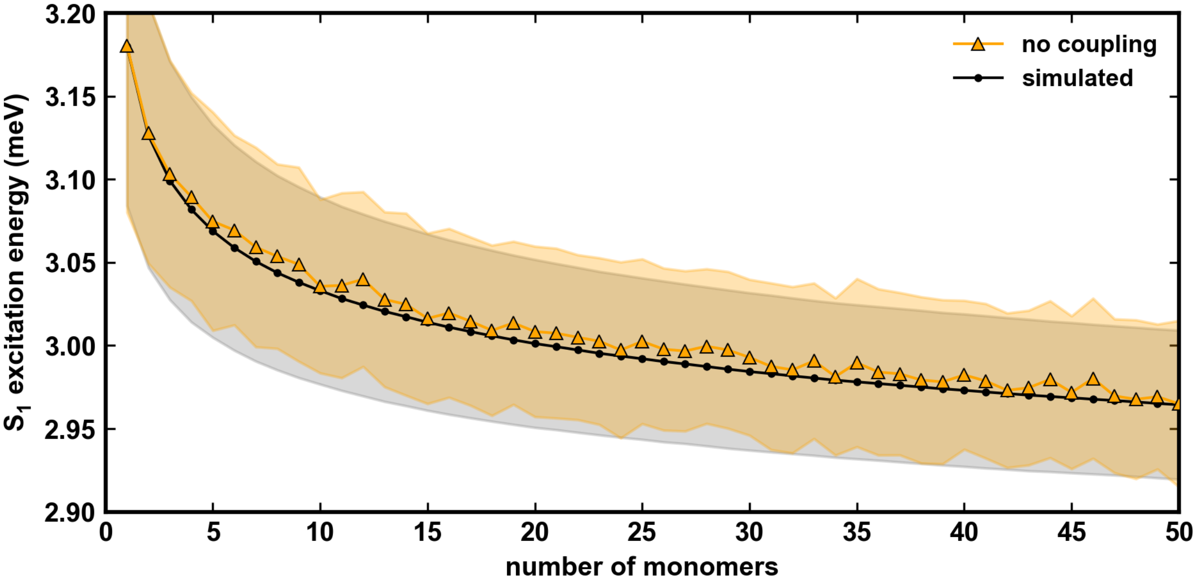

Figure S16. Simulated and model-predicted optical gaps when ignoring all couplings. Each point is the average value over 500 conformers with their one standard deviation depicted by colored area. The black line and grey area: from numerical simulation; Orange line and orange area: model predicted.

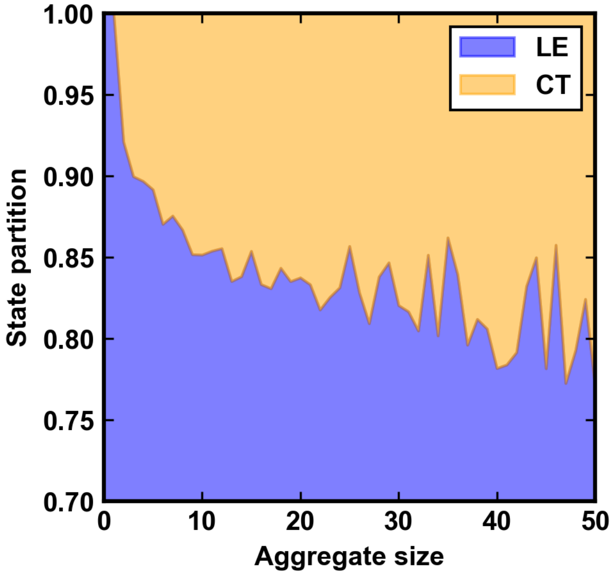

Figure S17. LE and CT state components of different aggregate's S_1 state.

Text S8. Detailed procedure of geniting the COM4A and NST5A subsets in the PrDim and TtDim datasets.

1. **Initial structure generation.** The forcefield of perylene monomer is generated by Antechamber,[6] using General Amber Force Field (GAFF) parameters[7] and AM1-BCC[8] to assign the atomic partial charges. Then, a 50×50 Å box containing 400 perylene molecules

is constructed by the Packmol[9] program based on the density of perylene α-crystal 1.35g/cm³. The final AMBER topology file is generated by the tleap program in the AmberTools.[10] The initial structure of amorphus tetracene system was generated with the same procedure and parameters except the reference density for Packmol was set to 1.24 g/cm³.[11]

2. **MD trajectory generation.** All MD simulations use the time step 2fs, with a nonbonded cutoff of 8.0 Å and periodic boundary conditions. We first performed 1000 steps of energy minimization with an energy tolerance of 1 kcal/mol. Then performed 2ps heating simulation to heat the system to 300K using Langevin dynamics with the friction parameter γ=2.0, followed by a 2ps NVE equilibrium simulation. The 50ns production run is performed under the NPT ensemble under 1 bar pressure and 300K, with the snapshot interval of 10000 steps (20ps).
3. **Dimer sampling and de-duplication.** The dimer sampling is completed by our in-house Python code using the package MDTraj.[12] We discarded the first 10 ns trajectory to ensure constant volume, then for the rest of 2000 frames, we applied the criteria of NST5A and COM4A datasets to sample all possible dimers. Since this will result in too many conformers (more than 100,000), we applied the furthest point sampling (FPS) algorithm based on the root mean square deviation (RMSD) between two dimer conformations to sample 8000 conformations from the sampled dataset.

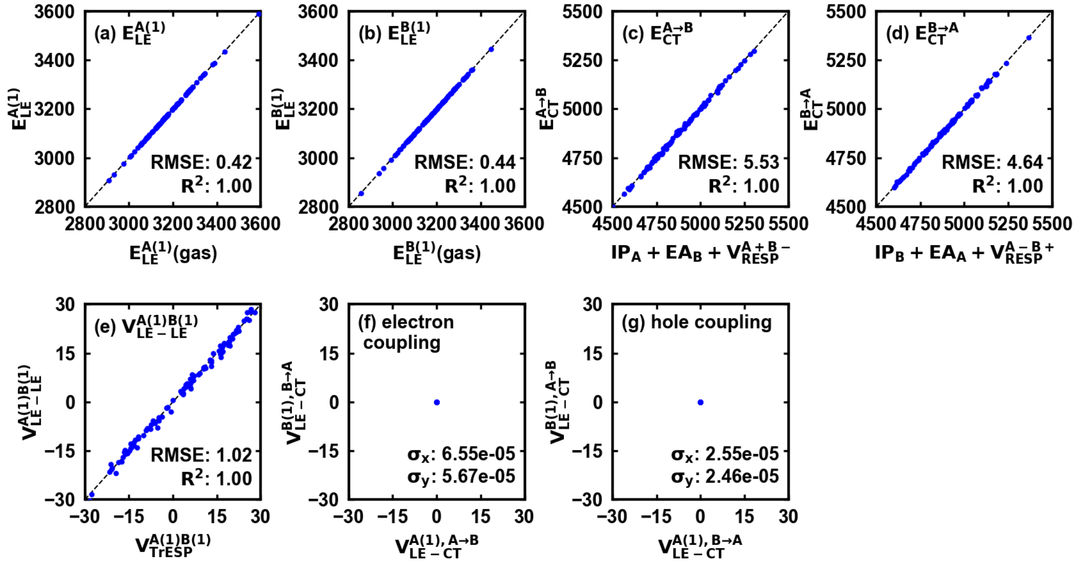

Figure S18. The approximation quality for the tested 100 separate perylene dimers. All numbers on the axes are in meV. (a) $E_{\mathrm{LE(gas)}}^{\mathrm{A(1)}}$ versus $E_{\mathrm{LE}}^{\mathrm{A(1)}}$. (b) $E_{\mathrm{LE(gas)}}^{\mathrm{B(1)}}$ versus $E_{\mathrm{LE}}^{\mathrm{B(1)}}$. (c) Reference $E_{\mathrm{CT}}^{\mathrm{A\to B}}$ versus Eq. (3)'s approximation. (d) Reference $E_{CT}^{B\to A}$ versus Eq. (3)'s approximation. (e) $V_{\mathrm{LE-LE}}^{\mathrm{A(1),B(1)}}$ versus the TrESP approximation; (f) The distribution of the two electron couplings ($V_{\mathrm{LE-CT}}^{\mathrm{A(1),A\to B}}$ and $V_{\mathrm{LE-CT}}^{\mathrm{B(1),B\to A}}$); (g) The distribution of the two hole couplings ($V_{\mathrm{LE-CT}}^{\mathrm{A(1),B\to A}}$ and $V_{\mathrm{LE-CT}}^{\mathrm{B(1),A\to B}}$). For subplot (a) to (e), the RMSE in meV and the R^2 are shown to prove accurate. For subplots (f) and (g), the standard deviation (in meV) of the two quantities labeled on the x-axis and y-axis are shown in order to show these couplings are negligible.

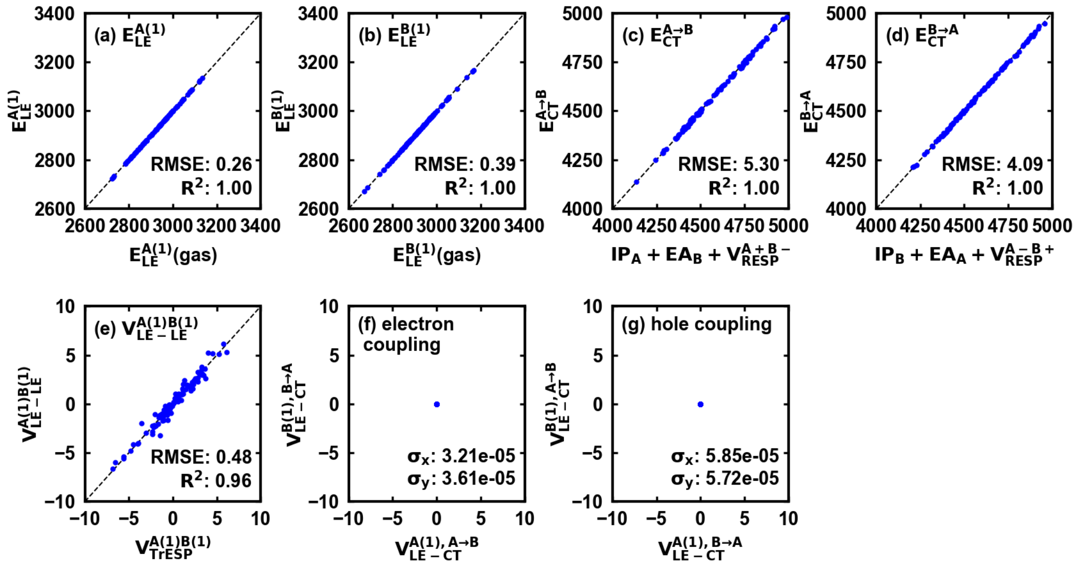

Figure S19. The approximation quality for the tested 100 separated tetracene dimers. All numbers on the axes are in meV. The meaning of each subplot is the same with **Figure S18**

Text S9. Detailed generation procedure for the out-of-sample datasets.

1. **Initial structure generation.** The perylene and tetracene monomer's forcefield is generated in the same way as the amorphous perylene or tetracene system. When generating the initial conformation of nanoaggregates containing 2 (oPrDim & oTtDim), 3 (oPrTri & oTtTri), and 4 (oPrTet & oTtTet) monomers, we use the Packmol program to pack the corresponding number of monomers inside a 10×10 Å box. Then the AMBER topology and coordinate file are generated by tleap as well.
2. **MD trajectory generation.** All MD simulations use the time step 2fs, with a nonbonded cutoff of 8.0 Å. No periodic boundary conditions are used. We first performed 1000 steps of energy minimization with an energy tolerance of 1 kcal/mol, in order to let the monomers aggregate via nonbonded forces. Then performed 2ps heating simulation to heat the system to 300K using Langevin dynamics with the friction parameter γ=2.0, followed by 2ps NVE equilibrium simulation. The 5ns production run is performed under the NVT ensemble under 300K with the snapshot interval of 5000 steps (10ps), resulting in 500 conformers. No further deduplication was performed.

.

Table S4. The hyperparameters of torchani.AEVComputer. Refer to the TorchANI's documentation for the exact meaning of these parameters.

Hyperparameter	Data types	Value
Rcr	float	7.5
Rca	float	3.5

EtaR	torch.tensor	16
ShfR	torch.tensor	0.9, 1.3125, 1.725, 2.1375, 2.55, 2.9625, 3.375, 3.7875, 4.2, 4.6125, 5.025, 5.4375, 5.85, 6.2625, 6.675, 7.0875
EtaA	torch.tensor	8
Zeta	torch.tensor	8
ShfA	torch.tensor	0.9, 1.55, 2.2, 2.85
ShfZ	torch.tensor	0.19634954, 0.58904862, 0.9817477, 1.37444679,1.76714587, 2.15984495, 2.55254403, 2.94524311

References

1. Li, X.; Parrish, R. M.; Liu, F.; Kokkila Schumacher, S. I. L.; Martínez, T. J., An Ab Initio Exciton Model Including Charge-Transfer Excited States. *Journal of Chemical Theory and Computation* **2017,** *13* (8), 3493-3504.
2. Canola, S.; Bagnara, G.; Dai, Y.; Ricci, G.; Calzolari, A.; Negri, F., Addressing the Frenkel and charge transfer character of exciton states with a model Hamiltonian based on dimer calculations: Application to large aggregates of perylene bisimide. *The Journal of Chemical Physics* **2021,** *154* (12), 124101.
3. Giannini, S.; Peng, W.-T.; Cupellini, L.; Padula, D.; Carof, A.; Blumberger, J., Exciton transport in molecular organic semiconductors boosted by transient quantum delocalization. *Nature Communications* **2022,** *13* (1), 2755.
4. Maj, M.; Jeon, J.; Góra, R. W.; Cho, M., Induced Optical Activity of DNA-Templated Cyanine Dye Aggregates: Exciton Coupling Theory and TD-DFT Studies. *The Journal of Physical Chemistry A* **2013,** *117* (29), 5909-5918.
5. Gajdos, F.; Valner, S.; Hoffmann, F.; Spencer, J.; Breuer, M.; Kubas, A.; Dupuis, M.; Blumberger, J., Ultrafast Estimation of Electronic Couplings for Electron Transfer between π-Conjugated Organic Molecules. *Journal of Chemical Theory and Computation* **2014,** *10* (10), 4653-4660.
6. Wang, J.; Wang, W.; Kollman, P. A.; Case, D. A., Antechamber: an accessory software package for molecular mechanical calculations. *J. Am. Chem. Soc* **2001,** *222* (1).
7. Wang, J.; Wolf, R. M.; Caldwell, J. W.; Kollman, P. A.; Case, D. A., Development and testing of a general amber force field. *Journal of Computational Chemistry* **2004,** *25* (9), 1157-1174.
8. Jakalian, A.; Jack, D. B.; Bayly, C. I., Fast, efficient generation of high-quality atomic charges. AM1-BCC model: II. Parameterization and validation. *Journal of computational chemistry* **2002,** *23* (16), 1623-1641.
9. Martínez, L.; Andrade, R.; Birgin, E. G.; Martínez, J. M., PACKMOL: A package for building initial configurations for molecular dynamics simulations. *Journal of Computational Chemistry* **2009,** *30* (13), 2157-2164.
10. Case, D. A.; Aktulga, H. M.; Belfon, K.; Cerutti, D. S.; Cisneros, G. A.; Cruzeiro, V. W. D.; Forouzesh, N.; Giese, T. J.; Götz, A. W.; Gohlke, H., AmberTools. *Journal of chemical information and modeling* **2023,** *63* (20), 6183-6191.
11. Robertson, J. M.; Sinclair, V. C.; Trotter, J., The crystal and molecular structure of tetracene. *Acta Crystallographica* **1961,** *14* (7), 697-704.

12. McGibbon, R. T.; Beauchamp, K. A.; Harrigan, M. P.; Klein, C.; Swails, J. M.; Hernández, C. X.; Schwantes, C. R.; Wang, L.-P.; Lane, T. J.; Pande, V. S., MDTraj: a modern open library for the analysis of molecular dynamics trajectories. *Biophysical journal* **2015,** *109* (8), 1528-1532.
