# Supplementary material for: Size-Transferable Prediction of Excited State Properties for Molecular Assemblies with a Machine Learning Exciton Model
Source: J Phys Chem Lett. 2025 Mar 3;16(10):2541–52. doi: 10.1021/acs.jpclett.4c03548 (PMC11912531; doi:10.1021/acs.jpclett.4c03548)
Supplement: Supplementary file 2 — jz4c03548_si_002.pdf [file jz4c03548_si_002.pdf]

jz-2024-035488.R1

Name: Peer Review Information for "Size-transferable prediction of excited state properties for molecular assemblies with machine-learned exciton model"

First Round of Reviewer Comments

Reviewer: 1

Comments to the Author

The manuscript by Liu and co-workers developed machine-learning (ML) models for ab initio extended Frenkel exciton model which includes charge-transfer (CT) states, and showed that their ML models trained against dimer data can be applied to larger aggregates, suggesting their good transferability. Their models could be useful for the exciton dynamical simulations of large systems, and the paper is well written. I support its publication in JPCL given its novelty in ML models and thoroughness. However, I hope my comments below, mostly concerning their discussions about previous works, could improve their manuscript:

1. The authors mentioned previous “ML models are not transferrable, insufficient to investigate nanosized molecule assemblies exhibiting size heterogeneity.” I disagree with this statement as no evidence is provided. Many previous ML models learned site energies and couplings, sometimes even in the presence of MM environment, based on quantum calculations on monomers or dimers extracted from aggregates. Although they may not be explicitly trained against dimer configurations from dimer MD simulations, they effectively took single or two chromophores from the MD simulations of larger systems to perform quantum calculations for ML model training. In principle, they may be also transferrable, and can be applied to larger systems (in some cases, they were indeed applied to larger systems).

2. The authors mentioned “Furthermore, the Frenkel Hamiltonians predicted by previous studies30-31 usually ignore the CT state energies and the LE-CT couplings despite their significant impact on the excited state energy of PAH dimers.” This statement may be potentially misleading. There are many recent works that include CT states in their exciton models. Just list a few (not exhaustive): JPCL 9 (2018) 6892, Photosynthesis Research 137 (2018) 215, JPCL 8 (2017) 1442, etc. I guess the authors mean there are no ML models trained for exciton models that include CT states. I suggest the authors cite previous works that include CT states in their exciton models, and rephrase this sentence to avoid potential misunderstanding.

3. The authors mentioned “Such a phase problem was mentioned in previous studies about excited state ML47-48 but no systematic solution was available.” This is not true: this phase factor issue is well-known in the communities, and systematic solutions are available (although some may not consider CT states). Just list a few (not exhaustive): SI of PCCP 26 (2024) 1023, Appendix of JCP 149 (2018) 094110, Section 5.3 of Chem Rev 121 (2021) 9873, and PCCP 26 (2024) 4306, etc. The author should revise the texts related to this point (including the abstract).

4. The explanations of the ML models need to be improved. What are those “approximations” for different models? They were mentioned, but were not clearly explained, and Fig. S3 is not very helpful (e.g., how to do decomposition?) How do they compute the reference monomer wave function, and how do they align reference wave function? Some schematics might be helpful.

5. It seems that their ML models still rely on some quantum chemical calculations, e.g., in order to get reference wave function, transition charges, RESP charges, etc. These input features may have improved the performance of their ML models, but would they substantially increase the cost of their ML models (i.e., generating input takes much longer than usual geometry-based input?). The authors may discuss the tradeoff between the accuracy and efficiency in their models.

6. Refs. 16 and 42 are duplicate.

Reviewer: 2

Comments to the Author

The authors report an ML model for excitons in large molecular assemblies. They include both, LE and CT states, the approach is interesting, the topic interesting, and the paper is definitely worth publishing. I am not sure, however, whether JPCletters is the right format, because it is a rather basic technical work, and not of utmost importance for a general audience. I have some minor remarks, which however question also the novelty a little:

The authors mention scalability as a major issue: I guess, training a Frenkel Hamiltonian should naturally solve that issue. Clearly, the data set is a critical point, but I guess some of the ML model cited are trained on dimers and can be applied to pretty large complexes. The authors may want to be more specific here.

The phase problem has been addressed in other papers than Refs 47/48 as mentioned by the authors. The problem has been address in CT simulations (J. Phys. Chem. A 2019, 123, 7792. J. Chem. Theory Comput. 2023, 19, 3825).

3) it is well known, that vertical excitation energies are not very accurate using DFT: why not scale the site energies to match higher level calculations or experiment?

The overall work, however, is very solid and especially the inclusion of CT states is notable.

But why not shoot to JPC A/B/C with the reviewer comments

Reviewer: 3

Comments to the Author

This work by Ren et al. presents the development and validation of a protocol for constructing excitonic Hamiltonians (including local and charge transfer excitations) based on calculations performed on dimer geometries sampled along MD trajectories. The study is both innovative and well-executed, though in some cases I believe a better comparison with existing methods would be needed and would strengthen the significance of the work. I would recommend the paper for publication, provided the following questions and comments are carefully addressed.

1) On page 6, the authors mention that the SEP5A test set was generated using approximations to reduce computational cost. However, it was not immediately clear what they meant by this until I referred to the Supporting Information (SI). I believe it is important to provide a more detailed discussion of their models for COM4A, NST5A, SEP5A, and the related approximations directly in the main text. In particular, the authors should clearly explain how they utilize TrESP charges and orbital overlap within the main text.

2) About the computational cost, would be useful to add somewhere in the text or SI a table summarizing Regarding the computational cost, it would be useful to include a table, either in the main text or the SI, summarizing the timings of the various computations (e.g., full TDDFT, the excitonic model, and the ML excitonic model).

3) In this context, the authors should also provide a more detailed discussion of the advantages of using ML models in terms of computational cost and accuracy compared to other existing fast analytic methods for evaluating excitonic and electronic couplings, such as the Coulomb interaction between transition charges and the Analytic Overlap method between orbitals). (https://onlinelibrary.wiley.com/doi/abs/10.1002/wcms.1392, https://pubs.acs.org/doi/10.1021/acs.jctc.3c00184, https://doi.org/10.1063/5.0076555). Indeed, excitonic Hamiltonians have already been constructed and employed to determine the optical properties of extended aggregates using these simplified methods, which account for the correct coupling-sign relationships. The authors should acknowledge and discuss this existing body of

literature: https://linkinghub.elsevier.com/retrieve/pii/S1369702124002116, https://xlink.rsc.org/?DOI=D4TC01716A).

4) It's not quite clear whether the ML protocol they have developed can deal with excitations that not completely HOMO-LUMO (as for instance those in DNA nucleo basis). In general, I believe the authors should discuss more clearly the limitations of their model.

5) The authors should explicitly mention and discuss the differences between their approach to obtaining reference data, as implemented in TeraChem, and other existing schemes for constructing excitonic Hamiltonians in a localized (diabatic) basis. For example, diabatization schemes such as the multi-state fragment excitation energy difference-fragment charge difference method (MS-FED-FCD) : https://doi.org/10.1063/5.002267, https://www.nature.com/articles/s41467-022-30308-5 and similar schemes https://pubs.acs.org/doi/10.1021/acs.jctc.1c00416 should be acknowledged and discussed. These approaches incorporate multiple adiabatic states in the diabatization process and enable the calculation of accurate diabatic couplings, even in cases where many adiabatic states are strongly mixed. A discussion in comparison with such methods would provide valuable context for evaluating the advantages and limitations of the authors' approach.

6) The author should comment on whether they could train their model against the previous diabatization schemes to build Hamiltonian in localized (diabatic basis). In principle, I assume this might allow the authors to go beyond the need to include only HOMO-LUMO transition.

7) It would be helpful if the authors could comment on the possibility of obtaining the gradients of their excitonic Hamiltonian. This capability could facilitate incorporating the model into simulations for excited-state dynamics.

8) Additionally, in the Supporting Information (SI), the authors mention the use of different coefficients for FE-CT and CT-CT couplings in hole and electron couplings. While this is an interesting observation, I believe the authors should provide further reasoning and discussion to explain why this discrepancy arises.

9) Please check SI pag S14: (Error! Reference source not found.)

Author's Response to Peer Review Comments:

Fang Liu
Assistant Professor
1515 Dickey Drive
Atlanta, GA 30322
Tel: (404)-727-6731
Email: fang.liu@emory.edu

February 15, 2025

Dear Reviewers,

Thanks for your comments. We have made some changes to improve the clarity of the manuscript. We detail these and responses to the reviewers' questions below. We have also uploaded a version of the manuscript with highlighted text to facilitate the reviewing process. The new text is colored blue with an underline, and the deleted text is colored ~~red and denoted with a strikethrough~~. At the end of this letter, we also summarize our responses to your comments in the decision letter.

Reviewer #1:

1) The manuscript by Liu and co-workers developed machine-learning (ML) models for ab initio extended Frenkel exciton model which includes charge-transfer (CT) states, and showed that their ML models trained against dimer data can be applied to larger aggregates, suggesting their good transferability. Their models could be useful for the exciton dynamical simulations of large systems, and the paper is well written. I support its publication in JPCL given its novelty in ML models and thoroughness

We thank the reviewer for recognizing the significance of our work and recommendation for publication.

2) The authors mentioned previous "ML models are not transferrable, insufficient to investigate nanosized molecule assemblies exhibiting size heterogeneity." I disagree with this statement as no evidence is provided. Many previous ML models learned site energies and couplings, sometimes even in the presence of MM environment, based on quantum calculations on monomers or dimers extracted from aggregates. Although they may not be explicitly trained against dimer configurations from dimer MD simulations, they effectively took single or two chromophores from the MD simulations of larger systems to perform quantum calculations for ML model training. In principle, they may be also transferrable, and can be applied to larger systems (in some cases, they were indeed applied to larger systems).

We thank the reviewer for pointing this out and agree with this comment. Indeed, some models trained from previous works may have the potential of size-transferability. This is because they used subsystems of monomer and dimer to train their models and predicted the Hamiltonian of larger molecular assemblies such as light-harvesting complexes. However, for closely packed PAH assemblies, the CT energies, together with LE-CT couplings play a significant role in their excited state properties, which were not readily available in previous work. We acknowledged these studies in our main text and highlighted the specific differences between our model and previous work.

Page 4 (PDF with markup):

Despite its success in small PAH aggregates,[27-29] the fragment-based Frenkel exciton model still requires significant computing resources for modeling the excited states for nanosized aggregates.

Fang Liu
Assistant Professor
1515 Dickey Drive
Atlanta, GA 30322
Tel: (404)-727-3721
Email: fang.liu@emory.edu

Since an aggregate's observed properties (e.g., absorption/fluorescence spectrum) are the statistical average over multiple conformations,[10, 30-31] accurately modeling these properties necessitates adequate sampling of the conformational space. However, the conformational space expands exponentially with the number of monomers, making this task computationally challenging. In recent years, many studies have tried to overcome this obstacle via machine-learned (ML) exciton models,[32-41] which predict the Frenkel Hamiltonian matrix elements for aggregates of a fixed size based on their conformation. The training data are reference Frenkel Hamiltonians for molecular dynamics (MD) sampled aggregate conformations of the fixed size, calculated by ~~density functional theory (DFT)~~DFT or semi-empirical methods. These ML exciton models have been applied to molecular spectrum prediction, [38] excited state dynamics, .[32, 35, 42] and exciton and charge transfer simulation in organic semiconductors[43-45] and light-harvesting complexes.[46] However, these models require expensive reference Hamiltonians for large aggregates and cannot be directly applied to molecular assemblies exhibiting size-heterogeneity, ~~However, these ML models are not size-transferrable, insufficient to investigate nanosized molecular assemblies exhibiting size heterogeneity,~~ such as asphaltene aggregates[47] and carbon dots.[6] In contrast, some recent ML exciton models for biomolecules exhibit size-transferability,[35, 38, 46, 48-49] wherein a multi-chromophore system's Frenkel Hamiltonian is predicted by a model trained on QM calculations of subsystems containing one or two chromophores. Nevertheless, these models do not yet incorporate CT states and related couplings, which are essential for accurately predicting the optical properties of PAH aggregates.[50-52]

3) The authors mentioned "Furthermore, the Frenkel Hamiltonians predicted by previous studies30-31 usually ignore the CT state energies and the LE-CT couplings despite their significant impact on the excited state energy of PAH dimers." This statement may be potentially misleading. There are many recent works that include CT states in their exciton models. Just list a few (not exhaustive): JPCL 9 (2018) 6892, Photosynthesis Research 137 (2018) 215, JPCL 8 (2017) 1442, etc. I guess the authors mean there are no ML models trained for exciton models that include CT states. I suggest the authors cite previous works that include CT states in their exciton models, and rephrase this sentence to avoid potential misunderstanding.

We apologize for this misleading statement and appreciate the reviewer for the suggestion. We indeed meant there are no ML models trained for exciton models that include CT states. We rephrased the related paragraph to avoid this misunderstanding.

Page 5 (PDF with markup):

However, these models require expensive reference Hamiltonians for large aggregates and cannot be directly applied to molecular assemblies exhibiting size-heterogeneity, ~~However, these ML models are not size-transferrable, insufficient to investigate nanosized molecular assemblies exhibiting size heterogeneity,~~ such as asphaltene aggregates[47] and carbon dots.[6] In contrast, some recent ML exciton models for biomolecules exhibit size-transferability,[35, 38, 46, 48-49] wherein a multi-chromophore system's Frenkel Hamiltonian is predicted by a model trained on QM calculations of subsystems containing one or two chromophores. Nevertheless, these models do not yet incorporate CT states and

Fang Liu
Assistant Professor
1515 Dickey Drive
Atlanta, GA 30322
Tel: (404)-727-3721
Email: fang.liu@emory.edu

related couplings, which are essential for accurately predicting the optical properties of PAH aggregates.[50-52]

In addition, as the reviewer commented, we found that it's very important to acknowledge the previous work of constructing Frenkel Hamiltonians with CT states and compare them with TeraChem's exciton model, the theoretical framework our study based on. We roughly classified the ways of constructing Frenkel Hamiltonians with QM calculations into the eigenstate-based and fragment-based methods based on how the diabatic states are constructed. The eigenstate-based methods transform adiabatic excited states into diabatic states by maximizing a "localization function", followed by constructing the excitonic Hamiltonian under such basis, represented by the FED & FCD method and their combined approach with multiple LE states (MS-FED-FCD). The fragment-based methods started from individual QM calculations on monomers to obtain localized diabatic states, followed by computing their energies and couplings to construct the Hamiltonian, represented by the ab-initio Frenkel exciton model implemented in TeraChem. In principle, both class of methods can include both LE and CT states in their diabatic basis. We added a detailed discussion of previous works and cited them in our introduction part.

Page 3 (PDF with markup):

Here, the $|\Psi_n\rangle$ denotes a diabatic state basis function of an LE or CT state, E_n is the corresponding diabatic state energy, V_{mn} is the coupling between these two states, and the summation runs over all LE and CT states. The excited state characteristics can be derived from the eigenvectors of $\hat{\mathrm{H}}$. Various methods have been proposed to evaluate the matrix elements under the LE and CT state representation, which can be roughly classified into the eigenstate-based and fragment-based methods based on how the diabatic states are constructed. The eigenstate-based methods transform adiabatic excited states into diabatic states by maximizing a "localization function", followed by constructing the excitonic Hamiltonian under such basis. Typical methods include the Boys localization,[19] Fragment Charge Difference (FCD),[14] Fragment Excitation Difference (FED),[20] and the multistate FED-FCD approach (MS-FED-FCD).[12, 21] These methods require an excited state QM calculation of the whole system normally scales as $O(N^3)$ where N is the number of monomers. The fragment-based methods started from individual QM calculations on monomers to obtain localized diabatic states, followed by computing their energies and couplings to construct the Hamiltonian. The ab-initio Frenkel Exciton model implemented in Q-Chem[22-23] and TeraChem,[24-25] ~~,~~ together with the recent subsystem TDDFT-based MS-FED-FCD belongs to this class.[26] They reduce the scaling to below $O(N^2)$ with an energy difference smaller than 0.1 eV from full-electron methods.[22-25] ~~When evaluating the $\hat{\mathrm{H}}$ matrix elements under the diabatic state representation, one can either use approximated expressions (e.g., estimate LE-LE coupling by atomic transition charges and LE-CT, CT-CT couplings by orbital overlap integral) to balance between accuracy and computational cost.or compute them with ab initio QM methods to achieve higher accuracy. Compared to full-electron methods, an efficiently implemented ab initio Frenkel exciton model reduces the time scaling from $O(N^3)$ to below $O(N^2)$, with an energy difference smaller than 0.1 eV from full-electron methods.~~

Fang Liu
Assistant Professor
1515 Dickey Drive
Atlanta, GA 30322
Tel: (404)-727-3721
Email: fang.liu@emory.edu

4) The authors mentioned "Such a phase problem was mentioned in previous studies about excited state ML47-48 but no systematic solution was available." This is not true: this phase factor issue is well-known in the communities, and systematic solutions are available (although some may not consider CT states). Just list a few (not exhaustive): SI of PCCP 26 (2024) 1023, Appendix of JCP 149 (2018) 094110, Section 5.3 of Chem Rev 121 (2021) 9873, and PCCP 26 (2024) 4306, etc. The author should revise the texts related to this point (including the abstract).

We thank the reviewer for pointing this out and agree with the reviewer's comment. The phase problems are indeed mentioned in previous studies and addressed in various ways. However, these methods are not very suitable for our planar PAH system with high symmetry, especially for predicting the Hamiltonian of larger aggregates. That is the reason why we proposed a new approach for phase-correction. We adjusted the text in the abstract and acknowledged these approaches in our main text, then discussed why our approach is necessary.

Page 1 (PDF with markup):

We also ~~systematically addressed~~proposed a new method to address the phase-correction problem by introducing coupling terms' approximations.

Page 13 (PDF with markup):

Such a phase problem was mentioned in previous studies about excited state ML[65] and several solutions have been proposed. ~~but no systematic solution was available~~. This includes (1) aligning the wave function's phase or CI coefficient between neighboring MD snapshots[66-67] and (2) correcting the phase based on the molecule's geometry.[48] However, approach (1) cannot be applied to randomly sampled dimer conformations, while approach (2) may be unavailable in highly symmetric planar PAH systems. Another alternative, the phase-free training approach,[36, 68] allows the model to learn the phase combination for a given system's Hamiltonian without prior phase correction, but such learned combination cannot be transferred to larger aggregates. Here, we find a solution for planar PAH aggregates of any size. When evaluating the Frenkel Hamiltonian of large aggregates, we align Ψ_{ref} ~~the previously computed reference monomer wave function~~ to each monomer in the aggregates. Then, we computed the approximations $\tilde{E}^{Y}$ between each dimer pair by our approach described in previous paragraphs, but the sign of MO coefficients and TrESP charges are not modified. These approximations allow the model to infer the signs of the coupling terms and yield a physically valid Hamiltonian matrix and thus predict the correct eigenvalues (excitation energies) and eigenvectors (aggregate wave function).

5) The explanations of the ML models need to be improved. What are those "approximations" for different models? They were mentioned, but were not clearly explained, and Fig. S3 is not very helpful (e.g., how to do decomposition?) How do they compute the reference monomer wave function, and how do they align reference wave function? Some schematics might be helpful.

Fang Liu
Assistant Professor
1515 Dickey Drive
Atlanta, GA 30322
Tel: (404)-727-3721
Email: fang.liu@emory.edu

We thank the reviewer for the comment. Previously, we included the description about the approximations in the Computational detail section. The way of doing decomposition is also described in the SI. As the reviewer commented, we found our previous content arrangement is not straightforward enough for the readers to understand. However, due to the article length (~5000 words) of JPCL, the technical details cannot be fully included in the main text. Therefore, we first moved the description of doing the decomposition to **Text S2,** just after **Figure S3 & S4**, for a better explanation of **Figure S3**. Then, we moved the description of what the approximations are and how to compute them into our main text and added a schematic figure (**Figure S4**) in our SI.

Page 9 (PDF with markup):

The second difference is the augmented AEV,

$$\vec{G}_i^{Y,\mathrm{aug}} = \{\vec{G}_i^X, b_i, \tilde{E}_i^Y\}, \tag{6}$$

where $\vec{G}_i^X$ is the original TorchANI AEV for the i-th atom; b_i is a Boolean "belonging label" to indicate whether the atom belongs to monomer A or B; and $\tilde{E}_i^Y$ is the atomic decomposition of the approximation $\tilde{E}^Y$. ~~two augmentation entries, b_i and $\tilde{E}_i^Y$, are added to enhance our model performance. Specifically, the~~ The label b_i enables a single NN (e.g., the LE network) to predict two different matrix elements under the same category (e.g., $E_{\mathrm{LE}}^{\mathrm{A(1)}}$ and $E_{\mathrm{LE}}^{\mathrm{B(1)}}$) by reversing each atom's label. For perylene and tetracene, we train each model separately. Additional details about model architecture are illustrated in **Figure S3** and explained in **Text S1.**

The $\tilde{E}_i^Y$ term enhances the model's data efficiency because of the strong correlation between the model output (E^Y) and its approximation ($\tilde{E}^Y$). As shown in **Figure S4**, computing $\tilde{E}_i^Y$ starts from calculating a reference neutral, cation, and anion wave function (Ψ_{ref}, Ψ_{ref}^+, Ψ_{ref}^-) based on a DFT-optimized monomer geometry $\{\vec{R}_i^{\mathrm{ref}}\}$, followed by fitting the atomic charge for Ψ_{ref}^+ and Ψ_{ref}^- with the restrained electrostatic potential (RESP) approach[60] (denoted as $\{q_i^{\mathrm{ref},+}\}$, $\{q_i^{\mathrm{ref},-}\}$ respectively). The atomic transition charges $\{q_i^{\mathrm{ref,tr}}\}$ for the monomer's $S_0 - S_1$ excitation are also fitted by the TrESP approach[61-62] based on a TDDFT calculation. Given monomer A's geometry $\{\vec{R}_i^A\}$, in a certain dimer AB, we first compute the translation vector and rotation matrix that optimally aligns $\{\vec{R}_i^{\mathrm{ref}}\}$, with $\{\vec{R}_i^A\}$. Then, an approximate monomer wave function $\Psi_{\mathrm{A}}^{\mathrm{apx}}$ was constructed by moving the atomic orbital (AO) basis function centered at $\vec{R}_i^{\mathrm{ref}}$ to $\vec{R}_i^A$, followed by adjusting molecular orbital (MO) coefficients within the same AO shell based on the rotation matrix. The $\{q_i^{\mathrm{ref},+}\}$, $\{q_i^{\mathrm{ref},-}\}$ and $\{q_i^{\mathrm{ref,tr}}\}$ are assigned to $\{\vec{R}_i^A\}$ without changing their magnitude. Finally, we adjust the sign of HOMO and LUMO coefficients in $\Psi_{\mathrm{apx}}^{\mathrm{A}}$ to align with the wave function evaluated by TeraChem's exciton model and modify $\{q_i^{\mathrm{ref,tr}}\}$'s based on the transition dipole moments of the reference LE state. $\tilde{E}^Y$ for CT energies $E_{\mathrm{CT}}^{\mathrm{A\to B}}$($E_{\mathrm{CT}}^{\mathrm{B\to A}}$) is the Coulombic interaction between $\{q_i^{\mathrm{ref},+}\}$ on A (B) and $\{q_i^{\mathrm{ref},-}\}$ on B (A), while that of $V_{\mathrm{LE-LE}}^{\mathrm{A(1)B(1)}}$ is the interaction between $\{q_i^{\mathrm{ref,tr}}\}$ placed on A and B. $\tilde{E}^Y$ for hole (electron) couplings is the overlap integral between the HOMO (LUMO) of $\Psi_{\mathrm{A}}^{\mathrm{apx}}$ and $\Psi_{\mathrm{B}}^{\mathrm{apx}}$, denoted by $S_{\mathrm{HOMO}}^{\mathrm{AB}}$ ($S_{\mathrm{LUMO}}^{\mathrm{AB}}$). Given that PAHs' frontier orbitals are predominantly composed of p-orbital character,[63-64] to speed up the calculation without losing much accuracy, $S_{\mathrm{HOMO}}^{\mathrm{AB}}$($S_{\mathrm{LUMO}}^{\mathrm{AB}}$) are computed as the overlap between p-type basis functions multiplied by their HOMO (LUMO) coefficients. Finally, all $\tilde{E}^Y$ are

Fang Liu
Assistant Professor
1515 Dickey Drive
Atlanta, GA 30322
Tel: (404)-727-3721
Email: fang.liu@emory.edu

decomposed into $\tilde{E}_i^Y$ to augment the AEV (see **Text S2** for details). For perylene and tetracene, we train each model separately. Additional details about model architecture are illustrated in **Figure S3** and explained in **Text S2.**

6) It seems that their ML models still rely on some quantum chemical calculations, e.g., in order to get reference wave function, transition charges, RESP charges, etc. These input features may have improved the performance of their ML models, but would they substantially increase the cost of their ML models (i.e., generating input takes much longer than usual geometry-based input?). The authors may discuss the tradeoff between the accuracy and efficiency in their models.

We thank the reviewer for the suggestion. Indeed, the efficiency of ML methods is important. The reference wave function, together with TrESP and RESP charge fitting, only needed to be computed once at a reference geometry. Therefore, we evaluated the cost of feature generation and neutral network (NN) propagation for our out-of-sample test sets. The result shows that the ML model was over three magnitudes faster than QM methods and the RESP and TrESP charge interaction were cheap. Computing overlap integrals does take a significant amount of time. However, the overlap integral is crucial to indicate the phase of LE-CT coupling, so we cannot discard it without losing too much accuracy. We added a table to summarize the timing for feature generation and NN propagation, then compared them with the ab-initio exciton model and all-electron TDDFT.

Page 15 (PDF with markup):

Table 2. Timings for the ML-exciton model for different OOS datasets. Each OOS dataset contains 500 aggregate structures. The ML timings are measured with an Intel(R) Xeon(R) Silver 4210R CPU and one Nvidia RTX A4000 GPU, while the timing for QM calculations is the total GPU hours used with the same hardware set.

Dataset	Number of monomers	TrESP & RESP charge interaction time (s)	overlap integral time (s)	NN propagatio n time (s)	Total time (s)	ab-initio exciton model (h)	all-electron TDDFT (h)
oPrDim	2	0.05	6.41	0.79	7.25	31.88	23.59
oPrTri	3	0.11	9.11	1.46	10.68	49.55	45.68
oPrTet	4	0.24	14.01	2.80	17.05	77.02	78.29
oTtDim	2	0.04	5.92	0.92	6.88	21.26	15.25
oTtTri	3	0.11	7.96	1.52	9.59	40.45	31.98
oTtTet	4	0.23	11.83	2.88	14.91	60.30	56.71

To demonstrate the balance between the accuracy and efficiency of our model, we summarized the timing for predicting the OOS datasets and compared them with TeraChem's exciton model

Fang Liu
Assistant Professor
1515 Dickey Drive
Atlanta, GA 30322
Tel: (404)-727-3721
Email: fang.liu@emory.edu

and all-electron TDDFT. (**Table 2)** The ML Hamiltonian is over three orders of magnitude faster than QM methods~~d~~. The most time-consuming step for constructing the ML Hamiltonian is calculating the overlap integrals, a necessary approximation for reproducing the phase of LE-CT couplings.

7) Refs. 16 and 42 are duplicate.

We thank the reviewer for perceptively pointing this out. We examined the references and removed all duplicated ones.

Reviewer #2:

1) The authors report an ML model for excitons in large molecular assemblies. They include both, LE and CT states, the approach is interesting, the topic interesting, and the paper is definitely worth publishing. I am not sure, however, whether JPCletters is the right format, because it is a rather basic technical work, and not of utmost importance for a general audience…The overall work, however, is very solid and especially the inclusion of CT states is notable. But why not shoot to JPC A/B/C with the reviewer comments

We thank the reviewer for the recognition of the significance of our work and recommendation for publication. We also understand the reviewer's concerns about the audience in our article. Indeed, our research may be more intriguing to audiences with Frenkel exciton model and machine learning background. However, our approach addresses two critical issues in the current subfield: reference data generation and model transferability. These two issues are not only crucial in excited state machine learning but also have general importance in other machine learning research areas.

In this work, instead of directly predicting vertical excitation energies, we reduced the data generation cost and achieved the size-transferability by predicting Frenkel Hamiltonians calculated by fragment-based methods. Since the Frenkel Hamiltonian can be used for excited state dynamics simulations, this makes the significance of this study not limited to the exciton model community, but also helpful to other physicists or chemists studying molecular excited states. Moreover, our approach may inspire other scientists attempting to incorporate machine learning into their workflows to solve the transferability and data generation problems, especially in the context where ML methods have become mainstream tools in scientific research.

In addition, we utilized ML exciton model to predict the average optical gap of large PAH nanoaggregates and reproduced the quantum confinement effect. We also analyzed the impact of various couplings on the optical band gap of the aggregates. This not only provided valuable insights into the structure-property relationship of PAH assemblies but also shows that our method can be used to study assemblies of interest to experimental chemists such as carbon quantum dots, thereby helping in the design of new photoactive materials. Therefore, we believe that our work is of important value to the broader scientific community. Publishing in JPCL will better demonstrate the value of our research to the scientific community than other field-specific journals.

Fang Liu
Assistant Professor
1515 Dickey Drive
Atlanta, GA 30322
Tel: (404)-727-3721
Email: fang.liu@emory.edu

We added discussion of the necessity of using Frenkel exciton model in both optical property prediction and excited state dynamics at the beginning to point out our study's significance for the excited state dynamics community.

Page 2 (PDF with markup):

Computational modeling of the excited state of PAH assemblies is vital for providing theoretical insights and designing guidance for photoactive systems. However, direct quantum mechanical (QM) calculations, represented by density-functional theory (DFT) and its time-dependent form (TDDFT), are computationally too expensive for the PAH nanoaggregate's large sizes and the resulting delocalized adiabatic states are not well-suited for analyzing their excitation characteristics., while mMethods for solid-state materials, such as band-gap theory, are not applicable to their disordered local structure.[6] Moreover, all-electron QM calculations cannot directly provide localized diabatic states and couplings, the necessary part for modeling the PAH assemblies' intermolecular excitation energy[2, 11] and electron transfer[12-15] processes based on the Marcus Theory.[16]

At the end of the main text, we also specifically pointed out the problems needed to address to extend our method to broader fields, such as excited-state dynamics simulations and application to heteroaggregates such as carbon quantum dots.

Page 21 (PDF with markup):

Currently, our studies are confined to the homogeneous aggregates whose monomers' S_0-S_1 transition is mostly HOMO-LUMO transition. Because the strong correlation between LE-CT couplings and CT-CT couplings only holds for pure HOMO-LUMO excitations.[63] (**Text S3** for explanation) Such limitations hinder our model from predicting the CT-CT couplings from LE-CT couplings involving impure LE states under TeraChem's exciton model framework. Another limitation is the error caused by configuration and orbital delocalization.[29] The limited monomer LE states in the ML Hamiltonians is one of the major factors of this discrepancy, but it cannot be fully eliminated for ML models trained against fragment-based approaches. This may be addressed by predicting the Hamiltonian generated from eigenstate-based approaches such as MS-FED-FCD. Their Hamiltonian matrices are unitarily transformed from the diagonal matrices of the system's adiabatic state energies, so their eigenvalues match the all-electron TDDFT. However, the off-diagonal couplings will rely on the chemical environment around the corresponding dimer so additional environmental embedding is needed. Additionally, as the excited state gradient can also be evaluated utilizing NN's autodifferentiation, it would be possible to conduct ML-based excited state dynamics for large PAH assemblies if the above limitations have been addressed.

In summary, we presented a novel approach combining machine learning techniques with the Frenkel exciton model to enable optical property prediction for different-sized molecular assemblies. Our model was rigorously evaluated on perylene and tetracene aggregates, demonstrating its effective transferability to larger aggregates. By applying this model, we investigated the optical gap of nanosized molecular aggregates and explained their quantum-dot-

Fang Liu
Assistant Professor
1515 Dickey Drive
Atlanta, GA 30322
Tel: (404)-727-3721
Email: fang.liu@emory.edu

like optical gap size dependency. Future research will focus on including more monomer LE states and surrounding environments, together with extending the transferability of the excited state potential energy surface (PES) to different PAH monomers, facilitating the prediction of hetero-aggregates.

2) The authors mention scalability as a major issue: I guess, training a Frenkel Hamiltonian should naturally solve that issue. Clearly, the data set is a critical point, but I guess some of the ML model cited are trained on dimers and can be applied to pretty large complexes. The authors may want to be more specific here.

We thank the reviewer for the suggestion and agree with this comment. As indicated in some previous works, models trained on Frenkel Hamiltonian may also be size-transferable. They also used subsystems of monomer and dimer to train their models and predicted the Hamiltonian of larger molecular assemblies such as light-harvesting complexes. However, for closely packed PAH assemblies, the CT energies, together with LE-CT couplings, play a significant role in their excited state properties, which were not readily available in previous work. We acknowledged these studies in our main text and highlighted the specific differences between our model and previous work.

Page 4 (PDF with markup):

In recent years, many studies have tried to overcome this obstacle via machine-learned (ML) exciton models,[32-41] which predict the Frenkel Hamiltonian matrix elements for aggregates of a fixed size based on their conformation. The training data are reference Frenkel Hamiltonians for molecular dynamics (MD) sampled aggregate conformations of the fixed size, calculated by ~~density functional theory (DFT)~~DFT or semi-empirical methods. These ML exciton models have been applied to molecular spectrum prediction, [38] excited state dynamics, .[32, 35, 42] and exciton and charge transfer simulation in organic semiconductors[43-45] and light-harvesting complexes.[46] However, these models require expensive reference Hamiltonians for large aggregates and cannot be directly applied to molecular assemblies exhibiting size-heterogeneity, ~~However, these ML models are not size-transferrable, insufficient to investigate nanosized molecular assemblies exhibiting size heterogeneity,~~ such as asphaltene aggregates[47] and carbon dots.[6] In contrast, some recent ML exciton models for biomolecules exhibit size-transferability,[35, 38, 46, 48-49] wherein a multi-chromophore system's Frenkel Hamiltonian is predicted by a model trained on QM calculations of subsystems containing one or two chromophores. Nevertheless, these models do not yet incorporate CT states and related couplings, which are essential for accurately predicting the optical properties of PAH aggregates.[50-52]

3) The phase problem has been addressed in other papers than Refs 47/48 as mentioned by the authors. The problem has been address in CT simulations (J. Phys. Chem. A 2019, 123, 7792. J. Chem. Theory Comput. 2023, 19, 3825).

We thank the reviewer for pointing this out. The phase problems are indeed mentioned in previous studies and addressed in various ways. However, these methods are not very suitable for our planar PAH system with high symmetry, especially for predicting the Hamiltonian of aggregates larger than the training set.

Fang Liu
Assistant Professor
1515 Dickey Drive
Atlanta, GA 30322
Tel: (404)-727-3721
Email: fang.liu@emory.edu

That is the reason why we proposed a new approach for phase-correction. We adjusted the text in the abstract and acknowledged these approaches in our main text, then discussed why our approach is necessary.

Page 1 (PDF with markup):

We also ~~systematically addressed~~ proposed a new method to address the phase-correction problem by introducing coupling terms' approximations.

Page 13 (PDF with markup):

Such a phase problem was mentioned in previous studies about excited state ML[65] and several solutions have been proposed. ~~but no systematic solution was available~~. This includes (1) aligning the wave function's phase or CI coefficient between neighboring MD snapshots[66-67] and (2) correcting the phase based on the molecule's geometry.[48] However, approach (1) cannot be applied to randomly sampled dimer conformations, while approach (2) may be unavailable in highly symmetric planar PAH systems. Another alternative, the phase-free training approach,[36, 68] allows the model to learn the phase combination for a given system's Hamiltonian without prior phase correction, but such learned combination cannot be transferred to larger aggregates. Here, we find a solution for planar PAH aggregates of any size. When evaluating the Frenkel Hamiltonian of large aggregates, we align Ψ_{ref} ~~the previously computed reference monomer wave function~~ to each monomer in the aggregates. Then, we computed the approximations $\tilde{E}^{Y}$ between each dimer pair by our approach described in previous paragraphs, but the sign of MO coefficients and TrESP charges are not modified. These approximations allow the model to infer the signs of the coupling terms and yield a physically valid Hamiltonian matrix and thus predict the correct eigenvalues (excitation energies) and eigenvectors (aggregate wave function).

4) It is well known, that vertical excitation energies are not very accurate using DFT: why not scale the site energies to match higher level calculations or experiment?

We appreciate the reviewer for this suggestion. As mentioned in benchmark articles (DOI: 10.1021/ct900298e), the DFT function wB97X-D3 has a systematic error of +0.21 eV in estimating the vertical excitation energy of PAH monomers. When discussing the result of large aggregates' optical gap, we applied the empirical correction to the site energies for all aggregates from 1 to 50 monomers. The optical gap predicted by the adjusted ML Hamiltonians are highly consistent with the experiments. However, manually scaling the site energies (LE energy) is not theoretically rigorous, as the CT energies and coupling elements may also require DFT functional-specific corrections. For example, the systematic error of the RSH functional for intermolecular CT excited states may be different than LE states, and the error of couplings has only been explored in individual studies under different theoretical frameworks. Therefore, our discussion focuses on the uncorrected results. We added the related discussion to the main text and added a figure in SI for illustration.

Page 19 (PDF with markup):

However, our model overestimates the optical gaps for both the monomer (3.18 eV) and the infinite-sized aggregate (2.70 eV) compared to the respective experimental values of 2.98 eV[76]

Fang Liu
Assistant Professor
1515 Dickey Drive
Atlanta, GA 30322
Tel: (404)-727-3721
Email: fang.liu@emory.edu

and 2.58 eV.[77] This overestimation is not due to the accuracy of the ML model, but is likely caused by the errors of the TDDFT method and the approximate density functional used for generating the training data. More specifically, this discrepancy is caused by the systematic error of +0.21 eV of TD-wB97X-D3 in computing the monomer LE state energies,.[78-80] as manually subtracting 0.21 eV from the LE state energies in the Hamiltonian improves the agreement with experiment (2.97 eV for monomer and 2.55 eV for infinite-sized aggregate) (**Figure S13**). However, this is not theoretically rigorous as the CT energies and coupling elements may also require DFT functional-specific corrections, which have only been explored in individual studies under different theoretical frameworks.[81] Therefore, our discussion focuses on the uncorrected results.

Reviewer #3:

1) This work by Ren et al. presents the development and validation of a protocol for constructing excitonic Hamiltonians (including local and charge transfer excitations) based on calculations performed on dimer geometries sampled along MD trajectories. The study is both innovative and well-executed, though in some cases I believe a better comparison with existing methods would be needed and would strengthen the significance of the work. I would recommend the paper for publication, provided the following questions and comments are carefully addressed.

We thank the reviewer for the recognition of the significance of our work and recommendation for publication. We also accepted the reviewer's suggestion about comparison with existing methods and did the suggested modifications.

2) On page 6, the authors mention that the SEP5A test set was generated using approximations to reduce computational cost. However, it was not immediately clear what they meant by this until I referred to the Supporting Information (SI). I believe it is important to provide a more detailed discussion of their models for COM4A, NST5A, SEP5A, and the related approximations directly in the main text. In particular, the authors should clearly explain how they utilize TrESP charges and orbital overlap within the main text.?

We thank the reviewer for the comment. To provide more information about our approaches for generating the SEP5A dataset, we moved the corresponding text to the computational detail section in our article.

Page 22 (PDF with markup):

Data Sets and Calculations For perylene aggregate data set generation, we first generated a 50 ns classical molecular dynamic (MD) simulation trajectory of an amorphous condensed perylene system containing 400 monomers with Amber[85] and AmberTools,[86] with detailed MD simulation and conformation sampling procedure given in **Text S8**. With the MD trajectory, we curated the

Fang Liu
Assistant Professor
1515 Dickey Drive
Atlanta, GA 30322
Tel: (404)-727-3721
Email: fang.liu@emory.edu

COM4A and NST5A subset of the dimer set PrDim. ~~a dimer set, PrDim (containing 24000 dimers), composed of three subsets: COM4A, NST5A, and SEP5A. Here, COM4A and NST5A~~The two subsets were extracted from the trajectory based on different criteria: COM4A selected dimers with all atoms in monomer A to be within 4 Å of monomer B's center of mass (COM),[57] and NST5A selected the dimers with the shortest pairwise distance between the two monomers to be less than 5 Å. COM4A and NST5A were further refined to contain 8000 dimers each by sampling with the furthest point sampling (FPS) algorithm.

The SEP5A subset for PrDim was generated by randomly adding an extra 5-8 Å to the COMs separation of NST5A's dimer structures. Corresponding Frenkel Hamiltonians are built with approximations by reusing the NST5A set's exciton model results due to their identical monomer structures. Specifically, the diagonal LE state energies are approximated as $\mathrm{E}_{\mathrm{LE(gas)}}^{\mathrm{A(1)}}$ for the corresponding entries in the NST5A set, as the $\Delta\hat{\mathrm{H}}_{\mathrm{BA}}$ terms in text Eq. (2) are negligible for distantly separated dimers (**Figure S3**). The diagonal CT state energies were calculated based on

$$\mathrm{E}_{\mathrm{CT}}^{\mathrm{A\to B}} = \mathrm{IP_A} + \mathrm{EA_B} + \mathrm{V}_{\mathrm{RESP}}^{\mathrm{A+B-}} + \sum_{\mathrm{C\neq A,C\neq B}} \langle \Psi_{\mathrm{CT}}^{\mathrm{A\to B}} | \Delta\hat{\mathrm{H}}_{\mathrm{C(AB)}} | \Psi_{\mathrm{CT}}^{\mathrm{A\to B}} \rangle, \quad (4)$$

where $\mathrm{IP_A}$ is monomer A's ionized potential, $\mathrm{EA_B}$ is monomer B's electron affinity, and $\mathrm{V}_{\mathrm{RESP}}^{\mathrm{A+B-}}$ is the intermolecular coulombic interaction calculated by the RESP charges on cationic A and anionic B. All terms in Eq. (8) are readily available in TeraChem's output files in the NST5A set. The LE-LE couplings are estimated by the TrESP approach[61] using Multiwfn [87] based on the electrostatic potentials of each monomer's $\mathrm{S_1}$ transition density, which was obtained from the computed monomer wave function in NST5A. The LE-CT couplings are set to 0 as they are in the order of 10^{-5} meV for separated dimers.[51] The three subset forms the PrDim training set with 24000 dimer structures. Our approximations Hamiltonian terms of SEP5A were proven to be very accurate (R^2>0.99) compared to ab initio exciton model calculations and significantly saved computation time (**Figure S18-S19**). Using the same procedure, we curated the tetracene dimer training set TtDim containing 24,000 dimers.

We also described how the approximations are computed in the main text, including how we utilize the TrESP charge and overlap integrals for feature generation and model training. We also added a schematic figure in the SI for more straightforward understanding.

Page 9 (PDF with markup):

The second difference is the augmented AEV,

$$\vec{G}_i^{Y,\mathrm{aug}} = \{\vec{G}_i^X, b_i, \tilde{E}_i^Y\}, \quad (6)$$

where $\vec{G}_i^X$ is the original TorchANI AEV for the i-th atom; b_i is a Boolean "belonging label" to indicate whether the atom belongs to monomer A or B; and $\tilde{E}_i^Y$ is the atomic decomposition of the approximation $\tilde{E}^Y$. ~~two augmentation entries, b_i and $\tilde{E}_i^Y$, are added to enhance our model performance. Specifically, the~~ The label b_i enables a single NN (e.g., the LE network) to predict two different matrix elements under the same category (e.g., $E_{\mathrm{LE}}^{\mathrm{A(1)}}$ and $E_{\mathrm{LE}}^{\mathrm{B(1)}}$) by reversing each atom's label. For perylene and tetracene, we train each model separately. Additional details about model architecture are illustrated in **Figure S3** and explained in **Text S1.**

Fang Liu
Assistant Professor
1515 Dickey Drive
Atlanta, GA 30322
Tel: (404)-727-3721
Email: fang.liu@emory.edu

The $\tilde{E}_i^Y$ term enhances the model's data efficiency because of the strong correlation between the model output (E^Y) and its approximation ($\tilde{E}^Y$). As shown in **Figure S4**, computing $\tilde{E}_i^Y$ starts from calculating a reference neutral, cation, and anion wave function (Ψ_{ref}, Ψ_{ref}^{+}, Ψ_{ref}^{-}) based on a DFT-optimized monomer geometry $\{\vec{R}_i^{\text{ref}}\}$, followed by fitting the atomic charge for Ψ_{ref}^{+} and Ψ_{ref}^{-} with the restrained electrostatic potential (RESP) approach[60] (denoted as $\{q_{\text{i}}^{\text{ref},+}\}$, $\{q_{\text{i}}^{\text{ref},-}\}$ respectively). The atomic transition charges $\{q_{\text{i}}^{\text{ref,tr}}\}$ for the monomer's $S_0 - S_1$ excitation are also fitted by the TrESP approach[61-62] based on a TDDFT calculation. Given monomer A's geometry $\{\vec{R}_i^{\text{A}}\}$, in a certain dimer AB, we first compute the translation vector and rotation matrix that optimally aligns $\{\vec{R}_i^{\text{ref}}\}$, with $\{\vec{R}_i^{A}\}$. Then, an approximate monomer wave function $\Psi_{\text{A}}^{\text{apx}}$ was constructed by moving the atomic orbital (AO) basis function centered at $\vec{R}_i^{\text{ref}}$ to $\vec{R}_i^{A}$, followed by adjusting molecular orbital (MO) coefficients within the same AO shell based on the rotation matrix. The $\{q_{\text{i}}^{\text{ref},+}\}$, $\{q_{\text{i}}^{\text{ref},-}\}$ and $\{q_{\text{i}}^{\text{ref,tr}}\}$ are assigned to $\{\vec{R}_i^{A}\}$ without changing their magnitude. Finally, we adjust the sign of HOMO and LUMO coefficients in $\Psi_{\text{apx}}^{\text{A}}$ to align with the wave function evaluated by TeraChem's exciton model and modify $\{q_{\text{i}}^{\text{ref,tr}}\}$'s based on the transition dipole moments of the reference LE state. $\tilde{E}^Y$ for CT energies $E_{\text{CT}}^{\text{A}\to\text{B}}$($E_{\text{CT}}^{\text{B}\to\text{A}}$) is the Coulombic interaction between $\{q_{\text{i}}^{\text{ref},+}\}$ on A (B) and $\{q_{\text{i}}^{\text{ref},-}\}$ on B (A), while that of $V_{\text{LE-LE}}^{\text{A(1)B(1)}}$ is the interaction between $\{q_{\text{i}}^{\text{ref,tr}}\}$ placed on A and B. $\tilde{E}^Y$ for hole (electron) couplings is the overlap integral between the HOMO (LUMO) of $\Psi_{\text{A}}^{\text{apx}}$ and $\Psi_{\text{B}}^{\text{apx}}$, denoted by $S_{\text{HOMO}}^{\text{AB}}$ ($S_{\text{LUMO}}^{\text{AB}}$). Given that PAHs' frontier orbitals are predominantly composed of p-orbital character,[63-64] to speed up the calculation without losing much accuracy, $S_{\text{HOMO}}^{\text{AB}}$($S_{\text{LUMO}}^{\text{AB}}$) are computed as the overlap between p-type basis functions multiplied by their HOMO (LUMO) coefficients. Finally, all $\tilde{E}^Y$ are decomposed into $\tilde{E}_i^Y$ to augment the AEV (see **Text S2** for details). ~~For perylene and tetracene, we train each model separately. Additional details about model architecture are illustrated in **Figure S3** and explained in **Text S2.**~~

3) About the computational cost, would be useful to add somewhere in the text or SI a table summarizing Regarding the computational cost, it would be useful to include a table, either in the main text or the SI, summarizing the timings of the various computations (e.g., full TDDFT, the excitonic model, and the ML excitonic model).

We thank the reviewer for the suggestion. We added a table for summarizing the computational cost for the feature generation, neural network propagation, and comparison with the exciton model and all-electron TDDFT.

Page 14 (PDF with markup):

Table 2. Timings for the ML-exciton model for different OOS datasets. Each OOS dataset contains 500 aggregate structures. The ML timings are measured with an Intel(R) Xeon(R) Silver 4210R CPU and one Nvidia RTX A4000 GPU, while the timing for QM calculations is the total GPU hours used with the same hardware set.

Fang Liu
Assistant Professor
1515 Dickey Drive
Atlanta, GA 30322
Tel: (404)-727-3721
Email: fang.liu@emory.edu

Dataset	Number of monomers	TrESP & RESP charge interaction time (s)	overlap integral time (s)	NN propagation time (s)	Total time (s)	ab-initio exciton model (h)	all-electron TDDFT (h)
oPrDim	2	0.05	6.41	0.79	7.25	31.88	23.59
oPrTri	3	0.11	9.11	1.46	10.68	49.55	45.68
oPrTet	4	0.24	14.01	2.80	17.05	77.02	78.29
oTtDim	2	0.04	5.92	0.92	6.88	21.26	15.25
oTtTri	3	0.11	7.96	1.52	9.59	40.45	31.98
oTtTet	4	0.23	11.83	2.88	14.91	60.30	56.71

4) In this context, the authors should also provide a more detailed discussion of the advantages of using ML models in terms of computational cost and accuracy compared to other existing fast analytic methods for evaluating excitonic and electronic couplings, such as the Coulomb interaction between transition charges and the Analytic Overlap method between orbitals)…Indeed, excitonic Hamiltonians have already been constructed and employed to determine the optical properties of extended aggregates using these simplified methods, which account for the correct coupling-sign relationships. The authors should acknowledge and discuss this existing body of literature…

We thank the reviewer for the suggestions. We evaluated the accuracy and timing when replacing our ML model with TrESP and the analytic overlap method (AOM) to compute the couplings. However, we soon discovered that this did not reduce the number of NN forward propagations because the LE and CT state energies still require ML model prediction. The reasons are detailed in **Text S5** and are summarized below:

For a homogeneous aggregate with N monomers. TrESP or AOM approaches require N QM calculations to accurately obtain them, leading to no computational advantages. A simplified way is assuming the wave function of the monomer does not change with its configuration, resulting in using a set of frozen TrESP charges and wave function on each monomer to evaluate the couplings. These approaches avoided unnecessary QM calculations, but they are not yet available for estimating the LE and CT state energies. Therefore, the LE and CT state energies still need to be predicted by the ML model.

When evaluating the Hamiltonian of an aggregate with N monomers, one needs to consider $N(N-1)/2$ dimer pairs. If only the first LE states are considered, given dimer pair, our model will yield 2 LE energies, 2 CT energies, 1 LE-LE coupling, 2 hole couplings, and 2 electron couplings at once. These values except LE energy are unique. Therefore, traversing all dimer pairs with $N(N-1)/2$ NN propagations can predict all the N LE energies, $N(N-1)$ CT state energies, $N(N-1)/2$ LE-LE

Fang Liu
Assistant Professor
1515 Dickey Drive
Atlanta, GA 30322
Tel: (404)-727-3721
Email: fang.liu@emory.edu

couplings, $N(N-1)$ hole couplings, $N(N-1)$ electron couplings. Many of the $N(N-1)(N-2)$ CT-CT couplings are identical and can be accurately approximated by corresponding LE-CT couplings, resulting in negligible computational cost no matter which method is used. As predicting $N(N-1)$ CT state energies still require $N(N-1)/2$ NN propagations, using TrESP or AOM does not reduce the number of NN propagations, thus having identical efficiency with complete ML Hamiltonian.

It's worth noting that, the TrESP charge and AOM approach based on a frozen wave function coincided with the analytic approximations we used in training our model, where a reference wave function is utilized to compute these quantities. To justify our argument, we also disabled the NN module for predicting the couplings to save computational cost as much as possible. Our results are summarized in Figure S9 and Table S3 in our SI. In short, the error for all OOS datasets improved by around 5 meV while the timing does not significantly change. Therefore, our method strikes a balance between accuracy and computational cost. We acknowledge the previous works and add the related discussion after **Table 2** in our main text.

Page 14-15 (PDF with markup):

> To demonstrate the balance between the accuracy and efficiency of our model, we summarized the timing for predicting the OOS datasets and compared them with TeraChem's exciton model and all-electron TDDFT. (**Table 2**) The ML Hamiltonian is over three orders of magnitude faster than QM methods. The most time-consuming step for constructing the ML Hamiltonian is calculating the overlap integrals, a necessary approximation for reproducing the phase of LE-CT couplings. Some fast and analytic methods have been proposed to evaluate couplings, such as the TrESP approach[61-62] for LE-LE couplings and analytic overlap method (AOM) for LE-CT and CT-CT couplings.[63, 69-70] They have been used to construct Frenkel Hamiltonians for optical properties prediction or nonadiabatic dynamics simulation.[15, 71-72] However, in our case, the diagonal elements (LE and CT state energies) still need to be predicted by the ML model. As our model can yield diagonal and off-diagonal elements simultaneously, the incorporation of TrESP and AOM has negligible impact on computational efficiency (see **Text S5** for proof) while increasing the MAE for all OOS datasets for around 5 meV (**Figure S9** and **Table S3**). Therefore, the ML model strikes a balance between accuracy and efficiency.

5) It's not quite clear whether the ML protocol they have developed can deal with excitations that not completely HOMO-LUMO (as for instance those in DNA nuclei basis). In general, I believe the authors should discuss more clearly the limitations of their model.

We thank the reviewer for this suggestion. Because approximating CT-CT coupling with LE-CT coupling requires the transition to be HOMO-LUMO, our model is limited to pure HOMO-LUMO transitions. We added a detailed discussion at the end of our article to clearly point out our limitations, including the issue of the not complete HOMO-LUMO transitions.

Page 19 (PDF with markup):

> Currently, our studies are confined to the homogeneous aggregates whose monomers' S_0-S_1 transition is mostly HOMO-LUMO transition. Because the strong correlation between LE-CT

Fang Liu
Assistant Professor
1515 Dickey Drive
Atlanta, GA 30322
Tel: (404)-727-3721
Email: fang.liu@emory.edu

couplings and CT-CT couplings only holds for pure HOMO-LUMO excitations.[63] **(Text S3** for explanation) Such limitations hinder our model from predicting the CT-CT couplings from LE-CT couplings involving impure LE states under TeraChem's exciton model framework.

6) The authors should explicitly mention and discuss the differences between their approach to obtaining reference data, as implemented in TeraChem, and other existing schemes for constructing excitonic Hamiltonians in a localized (diabatic) basis. For example, diabatization schemes such as the multi-state fragment excitation energy difference-fragment charge difference method (MS-FED-FCD) …… should be acknowledged and discussed. These approaches incorporate multiple adiabatic states in the diabatization process and enable the calculation of accurate diabatic couplings, even in cases where many adiabatic states are strongly mixed. A discussion in comparison with such methods would provide valuable context for evaluating the advantages and limitations of the authors' approach.

We thank the reviewer for this suggestion. As the reviewer commented, we found that it's very important to acknowledge the previous work of constructing Frenkel Hamiltonians and compare them with TeraChem's exciton model, the theoretical framework our study based on. We roughly classified the ways of constructing Frenkel Hamiltonians with QM calculations into the eigenstate-based and fragment-based methods based on how the diabatic states are constructed. The eigenstate-based methods transform adiabatic excited states into diabatic states by maximizing a "localization function", followed by constructing the excitonic Hamiltonian under such basis, represented by the FED & FCD method and their combined approach with multiple LE states (MS-FED-FCD). The fragment-based methods started from individual QM calculations on monomers to obtain localized diabatic states, followed by computing their energies and couplings to construct the Hamiltonian, represented by the ab-initio Frenkel exciton model implemented in TeraChem. In principle, both class of methods can include both LE and CT states in their diabatic basis. We added a detailed discussion of previous works and cited them in our introduction.

Page 3 (PDF with markup):

Here, the $|\Psi_n\rangle$ denotes a diabatic state basis function of an LE or CT state, E_n is the corresponding diabatic state energy, V_{mn} is the coupling between these two states, and the summation runs over all LE and CT states. The excited state characteristics can be derived from the eigenvectors of $\hat{H}$. Various methods have been proposed to evaluate the matrix elements under the LE and CT state representation, which can be roughly classified into the eigenstate-based and fragment-based methods based on how the diabatic states are constructed. The eigenstate-based methods transform adiabatic excited states into diabatic states by maximizing a "localization function", followed by constructing the excitonic Hamiltonian under such basis. Typical methods include the Boys localization,[19] Fragment Charge Difference (FCD),[14] Fragment Excitation Difference (FED),[20] and the multistate FED-FCD approach (MS-FED-FCD).[12, 21] These methods require an excited state QM calculation of the whole system normally scales as $O(N^3)$ where N is the number of monomers. The fragment-based methods started from individual QM calculations on monomers to obtain localized diabatic states, followed by

Fang Liu
Assistant Professor
1515 Dickey Drive
Atlanta, GA 30322
Tel: (404)-727-3721
Email: fang.liu@emory.edu

computing their energies and couplings to construct the Hamiltonian. The ab-initio Frenkel Exciton model implemented in Q-Chem[22-23] and TeraChem,[24-25] —, together with the recent subsystem TDDFT-based MS-FED-FCD belongs to this class.[26] They reduce the scaling to below $O(N^2)$ with an energy difference smaller than 0.1 eV from full-electron methods.[22-25] When evaluating the $\hat{\mathrm{H}}$ matrix elements under the diabatic state representation, one can either use approximated expressions (e.g., estimate LE-LE coupling by atomic transition charges and LE-CT, CT-CT couplings by orbital overlap integral) to balance between accuracy and computational cost.or compute them with ab initio QM methods to achieve higher accuracy. Compared to full-electron methods, an efficiently implemented ab initio Frenkel exciton model reduces the time scaling from $O(N^3)$ to below $O(N^2)$, with an energy difference smaller than 0.1 eV from full-electron methods.

We also discussed the limitation of our approach for reference data generation, i.e., the fraction-based ab-initio exciton model in TeraChem. The results calculated by this fragment-based method are usually different from those of all-electron TDDFT, which is mainly due to the limited monomer LE state basis set and the neglect of polarization effects between monomers, as we discussed in the accuracy over OOS datasets. This may be addressed by predicting the Hamiltonian generated from eigenstate-based approaches such as MS-FED-FCD. Their Hamiltonian matrices are unitarily transformed from the diagonal matrices of the system's adiabatic state energies, so their eigenvalues match the all-electron TDDFT. However, the off-diagonal couplings will rely on the chemical environment around the corresponding dimer so additional environmental embedding is needed. We added the related discussion to our main text.

Page 19 (PDF with markup):

Another limitation is the error caused by configuration and orbital delocalization.[29] The limited monomer LE states in the ML Hamiltonians is one of the major factors of this discrepancy, but it cannot be fully eliminated for ML models trained against fragment-based approaches. This may be addressed by predicting the Hamiltonian generated from eigenstate-based approaches such as MS-FED-FCD. Their Hamiltonian matrices are unitarily transformed from the diagonal matrices of the system's adiabatic state energies, so their eigenvalues match the all-electron TDDFT. However, the off-diagonal couplings will rely on the chemical environment around the corresponding dimer so additional environmental embedding is needed.

7) The author should comment on whether they could train their model against the previous diabatization schemes to build Hamiltonian in localized (diabatic basis). In principle, I assume this might allow the authors to go beyond the need to include only HOMO-LUMO transition.

We thank the reviewer for this suggestion. Indeed, our model can also be trained against other fragment-based approaches with multiple LE and CT states, but TeraChem's GPU-accelerating feature greatly accelerates the dataset generation. Hence, we first focus on building ML models to predict the TeraChem calculated dimer Hamiltonian. We modified the related text as follows:

Fang Liu
Assistant Professor
1515 Dickey Drive
Atlanta, GA 30322
Tel: (404)-727-3721
Email: fang.liu@emory.edu

Page 7 (PDF with markup):

> This allows us to predict larger aggregate Hamiltonian based on a dimer Hamilton dataset that comprehends sufficiently sampled dimer conformations. The reference dimer Hamiltonian can also be constructed by other fragment-based approaches with multiple LE and CT states,[22-23, 54] but TeraChem's GPU-accelerating feature greatly accelerates the dataset generation.[55] Hence, we first focus on building ML models to predict the TeraChem calculated dimer Hamiltonian and then extend our approach for larger aggregates, which will be discussed in later paragraphs.

For the eigenstate-based diabatization schemes, such as MS-FED-FCD, predicting their Hamiltonian would result in exact the same eigenstate energies as all-electron TDDFT. However, the off-diagonal couplings will not only depend on the dimer pair's configuration but also rely on the chemical environment around the corresponding dimer. Therefore, additional environmental embedding is needed. In addition, as we previously mentioned, the limitation in pure HOMO-LUMO transition is mainly because of the CT-CT coupling issue, which, unfortunately, also exists in other diabatization schemes with multiple intermolecular CT states. We added related discussion at the end of the article.

Page 21 (PDF with markup):

> … Another limitation is the error caused by configuration and orbital delocalization,[29] which cannot be fully eliminated for fragment-based exciton models. This may be addressed by predicting the Hamiltonian generated from eigenstate-based approaches such as MS-FED-FCD. Their Hamiltonian matrices are unitarily transformed from the diagonal matrices of the system's adiabatic state energies, so their eigenvalues match the all-electron TDDFT. However, the off-diagonal couplings will rely on the chemical environment around the corresponding dimer so additional environmental embedding is needed. …

8) It would be helpful if the authors could comment on the possibility of obtaining the gradients of their excitonic Hamiltonian. This capability could facilitate incorporating the model into simulations for excited-state dynamics.

We thank the reviewer for this comment. As the excited state gradient can also be evaluated utilizing NN's autodifferentiation, it is possible to conduct ML-based excited state dynamics for large PAH assemblies. This would be helpful for applying our approach to a larger research community. However, one needs to overcome the HOMO-LUMO transition limitation and include more LE states, together with including environment embedding to conduct reliable excited-state dynamic simulation. We added the related discussion to the main text.

Page 21 (PDF with markup):

> Currently, our studies are confined to the homogeneous aggregates whose monomers' S_0-S_1 transition is mostly HOMO-LUMO transition. Because the strong correlation between LE-CT couplings and CT-CT couplings only holds for pure HOMO-LUMO excitations.[63] **(Text S3** for explanation) Such limitations hinder our model from predicting the CT-CT couplings from LE-CT couplings involving impure LE states under TeraChem's exciton model framework. Another

Fang Liu
Assistant Professor
1515 Dickey Drive
Atlanta, GA 30322
Tel: (404)-727-3721
Email: fang.liu@emory.edu

limitation is the error caused by configuration and orbital delocalization.[72] The limited monomer LE states in the ML Hamiltonians is one of the major factors, but this discrepancy cannot be fully eliminated for ML models trained against fragment-based approaches. This may be addressed by predicting the Hamiltonian generated from eigenstate-based approaches such as MS-FED-FCD. Their Hamiltonian matrices are unitarily transformed from the diagonal matrices of the system's adiabatic state energies, so their eigenvalues match the all-electron TDDFT. However, the off-diagonal couplings will rely on the chemical environment around the corresponding dimer so additional environmental embedding is needed. Additionally, as the excited state gradient can also be evaluated utilizing NN's autodifferentiation, it would be possible to conduct ML-based excited state dynamics for large PAH assemblies if the above limitations have been addressed.

9) Additionally, in the Supporting Information (SI), the authors mention the use of different coefficients for FE-CT and CT-CT couplings in hole and electron couplings. While this is an interesting observation, I believe the authors should provide further reasoning and discussion to explain why this discrepancy arises.

We thank the reviewer for the perceptive comment. We did further investigation on this discrepancy and summarized the result in **Text S4**, **Figure S7**, and **Table S2** of our SI. In short, this discrepancy arises from the long-range Hartree-Fock (HF) exchange partition and was not sensitive to the DFT functional used.

We denote the factor between the electron (hole) LE-CT and CT-CT couplings as c_e (c_h). Then, the approximation should be expressed as

$$V_{CT-CT}^{A\to B,A\to C} \approx c_e V_{LE-CT}^{B(1),B\to C}$$
$$V_{CT-CT}^{C\to A,B\to A} \approx c_h V_{LE-CT}^{B(1),C\to B}$$

We found this non-zero factor was caused by the Hartree-Fock exchange partition at the long-range part of the range-separated function wB97X-D3. This is because for pure HOMO-LUMO transition, according to the definition in TeraChem's exciton model, the difference between LE-CT coupling $V_{LE-CT}^{B(1),B\to C}$ and CT-CT coupling $V_{CT-CT}^{A\to B,A\to C}$ arises from the two-electron terms, which can be expressed as:

$$V_{LE-CT,2e}^{B(1),B\to C} = 2(h_B l_B|h_B l_C) - c_{HF}(h_B h_B|l_B l_C) - (1-c_{HF})(h_B l_B|f_{xc}|h_B l_C)$$

To investigate the origin of this factor, we changed the HF exchange factor c_{HF} in the RSH functional wB97X-D3. The default c_{HF} for its short-range (SR) and long-range (LR) parts are 0.2 and 0.8, respectively. We started from completely disable the range-separation by setting the range-separation parameter ω to zero, followed by adjusting the SR c_{HF} from 0.0 to 1.0. $c_e(c_h)$ are obtained by extracting all electron (hole) LE-CT couplings together in trimer Hamiltonians, then fit them with all electron (hole) CT-CT couplings in the same Hamiltonian. c_e decreased from 1.07 to 0.72 and c_h increased from 1.00 to 1.21, indicating their sensitivity against the c_{HF}.

Fang Liu
Assistant Professor
1515 Dickey Drive
Atlanta, GA 30322
Tel: (404)-727-3721
Email: fang.liu@emory.edu

We also investigated the impact of the range-separated factor, w, on c_e and c_h. Here the SR and LR c_{HF} are set to their default value, but ω varies from 0.0 a.u.$^{-1}$ to 0.5 a.u.$^{-1}$. Surprisingly, both c_e and c_h equals to 1.00 at the beginning, but gradually approaches that obtained when $c_{HF} = 1.0$, equivalent to the HF theory. This is because the interaction between PAH monomers is governed by long-range interactions, as when $\omega = 0.25$ a.u.$^{-1}$, the default ω in wB97X-D3, the LR exchange partition erf (ωr) is over 0.95 when 3.6 Å $< r <$ 4.0 Å, the typical intermolecular distance between face-to-face stacked PAH molecules.

To check whether this factor depends on the DFT functional f_{xc}, we evaluate the couplings with various DFT functionals. The correlations are very strong (R^2≈1.00) for all DFT functionals, while the deviation of c_e and c_h from 1 becomes serious when the functional has more HF exchange partition, but c_e and c_h does not rely on the specific DFT exchange and correlation functional itself. It is worth mentioning that c_e and c_h are very close to 1 for commonly used hybrid functionals without range separation, such as B3LYP and PBE0. Therefore, we believe that the origin of this parameter is most likely due to the implementation of the TeraChem exciton model itself, which is beyond the scope of this work. Since TeraChem's exciton model can predict the absorption spectra of large aggregates, we chose to accept this fact in the current work and include this parameter in our machine learning pipeline.

As the length of Letters type articles are limited and this topic is a little beyond the scope of this work, we placed the discussion and related results in the SI, with the main text modified to guide the readers to the related discussions.

Page 14 (PDF with markup):

> Fortunately, based on TeraChem's implementation of the exciton model,[24] such CT-CT couplings can be accurately approximated when the monomer S_0-S_1 transition is mostly a HOMO-LUMO transition. Perylene and tetracene happen to satisfy this condition (CI coefficient > 0.95). In these cases, the CT-CT coupling (e.g., $V_{\mathrm{CT-CT}}^{\mathrm{A\to B,A\to C}}$) can be approximated by multiplying a Hartree-Fock (HF) exchange partition dependent factor with the corresponding LE-CT coupling (e.g., $V_{\mathrm{LE-CT}}^{\mathrm{B(1),B\to C}}$), which are readily available from the ML models trained by the dimers. (see **Text S3** for detailed proof) We conducted test calculations on 100 perylene and tetracene trimers and confirmed the accuracy with MAE < 3 meV. (**Figure S5-S6**) More discussions about the factor between LE-CT and CT-CT couplings are available in **Text S4**, **Figure S7**, and **Table S2** in our supporting information.

10) Please check SI page S14: (Error! Reference source not found.)

We thank the reviewer for the perceptively point it out. We adjusted the link in SI and fixed this error.

Editor:

Fang Liu
Assistant Professor
1515 Dickey Drive
Atlanta, GA 30322
Tel: (404)-727-3721
Email: fang.liu@emory.edu

1) An email address is required for each corresponding author identified on the manuscript file. Please add the corresponding author email(s) to any page of the manuscript.

We have added the corresponding author email to the 1st page of the manuscript.

2) Please provide full contact information for all authors in manuscript file: institution, city, state, postal code, country for each affiliation (states are required for United States addresses only). Postal codes are also required for addresses outside the U.S., for countries that have them. Each separate affiliation requires its own address information; they cannot be combined.

We have added the superscript "1" after each author to indicate that the author address is the same for all the authors, with the address detailed below the author names.

3) The TOC graphic should fit in an area no larger than 3.25 in. × 1.75 in. (approx. 8.25 cm × 4.45 cm) and should have adequate resolution and clarity. Confirm that all text is legible at this size.

We updated the TOC graphic to be 3 in x 1.75 in, 600 dpi.

4) Please include author names, article titles, journal name, publication year, and at least the first page number for each reference citation for the following incomplete journal references: 38, 40, 58, 60, 63.

We updated the references with the required information.

5) Conferences/Reports: Please include author names, paper or chapter title, book or proceedings title, publisher, and year for the following incomplete reference: 68.

We added required information for reference 68 (now reference 91), which is a book chapter for a conference proceeding.

6) Please include author names, title of article, name of repository (section name, if provided), submission date, DOI or URL (accessed YYYY-MM-DD) for the following incomplete preprint references: 69.

We added the required information for the preprint 69 (now reference 92).

We believe we responded to all points the reviewers made and made appropriate changes to the paper and SI. We hope the manuscript is now suitable for publication. If there are any questions, please do not hesitate to contact me.

Fang Liu
Assistant Professor
1515 Dickey Drive
Atlanta, GA 30322
Tel: (404)-727-3721
Email: fang.liu@emory.edu

Sincerely,

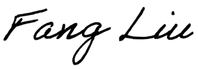

Fang Liu, Ph.D.
Assistant Professor
